# Four 14(13 → 12)-Abeolanostane Triterpenoids with 6/6/5/6-Fused Ring System from the Roots of *Kadsura coccinea*

**DOI:** 10.1007/s13659-019-0203-4

**Published:** 2019-04-11

**Authors:** Hou-Chao Xu, Kun Hu, Han-Dong Sun, Pema-Tenzin Puno

**Affiliations:** 1 State Key Laboratory of Phytochemistry and Plant Resources in West China, Kunming Institute of Botany, Chinese Academy of Sciences, and Yunnan Key Laboratory of Natural Medicinal Chemistry, Kunming, 650201 People’s Republic of China; 2 University of Chinese Academy of Sciences, Beijing, 100049 People’s Republic of China

**Keywords:** *Kadsura coccinea*, Lanostane triterpenoid, NMR computation, Anti-platelet aggregation, Anticoagulant

## Abstract

Four new rearranged 6/6/5/6-fused lanostane-type triterpenoids, kadcoccitanes A–D (**1**–**4**), were isolated from the roots of *Kadsura coccinea*, and their structures were mainly elucidated by comprehensive analysis of their spectroscopic data. Additionally, the structure of **1** was ambiguously verified by single-crystal X-ray diffraction, while the structure of **2**, which features a novel 8,16-epoxy motif, was validated by quantum chemical calculation of NMR parameters and ECD spectrum. Moreover, **1** and **4** were found to exhibited anticoagulant activity, while **3** and **4** were found to possess anti-platelet aggregation activity.

**Electronic supplementary material:**

The online version of this article (10.1007/s13659-019-0203-4) contains supplementary material, which is available to authorized users.

## Introduction

The plants of family Schisandraceae, including genera *Schisandra* and *Kadsura*, are invaluable sources of triterpenoids [1, 2]. *Kadsura coccinea* (Lem.) Smith is an economical fruit plant as well as a folk medicine usually employed for the treatment of gastropathy, rheumatic arthritis, postpartum abdominalgia with blood stasis, etc. [3]. Phytochemical investigations on the stems of *K. coccinea* collected from Guangxi and Yunnan Provinces have previously been undertaken by our research group, which revealed this species to be abundant with triterpenoids, especially lanostane triterpenoids [4–9]. Up to now, six kinds of unprecedented lanostane-type triterpenoid scaffolds have been isolated from the stems of *K. coccinea* by our group [4–7], namely, 14(13 → 12):13(17 → 16)-diabeolanostanes, 18(13 → 12)-abeo-13,17-secolanostanes, 14(13 → 12)-abeo-13,18-dinorlanostanes, 14(13 → 12)-abeo-12,13-secolanostanes, 14(13 → 12)-abeo-2,3-secolanostanes, and 13,14-secolanostanes. Besides, plenty of new triterpenoids belonging to conventional lanostanes [4–9], cycloartanes [10, 11] and schinortriterpenoids [10] have also been isolated from the stems of this species.

**Fig. 1:**
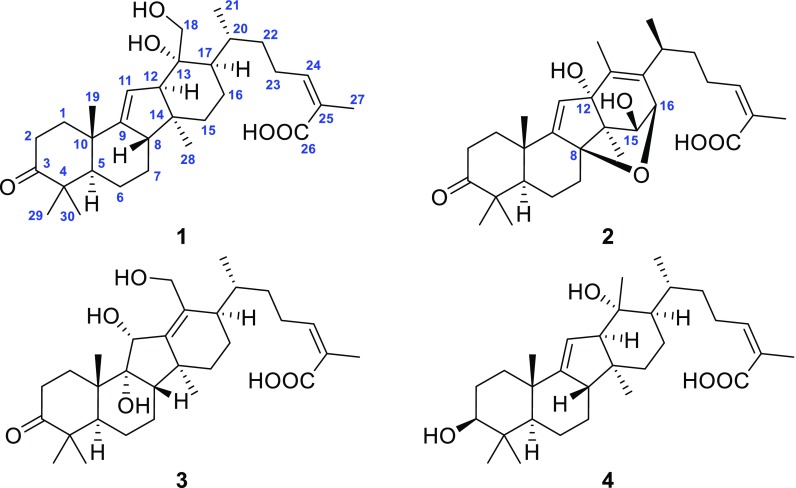
Chemical structures of compounds **1**–**4**

In the aforementioned research, the secondary metabolites of *K. coccinea* collected from different regions manifested significant differences. So, it was interesting to consider whether the secondary metabolites from different parts of the same plant differed or not. Thus, a phytochemical study on the roots of *K. coccinea* cultivated in Jingzhou Miao and Dong Autonomous County in Hunan Province was carried out. As a result, four new 14(13 → 12)-abeolanostane triterpenoids, kadcoccitanes A–D (**1**–**4**) were obtained (Fig. 1). Considering the folk medicinal value of *K. coccinea*, some of the compounds were tested for their anti-platelet aggregation and anticoagulant activities. Herein, the isolation, structure elucidation, and bioactivity screening of these compounds are described.

## Results and Discussion

Kadcoccitane A (**1**) was obtained as colorless acicular crystals and possessed a molecular formula of C_30_H_46_O_5_, which was determined by the HRESIMS ions at *m*/*z* 509.3251 [M + Na]^+^, calcd 509.3237), demonstrating eight degrees of unsaturation. The ^1^H-NMR data of **1** (Table 1) indicated the existence of five singlet methyls (*δ*_H_ 1.06, 1.15, 1.25, 1.39 and 2.14), one doublet methyl (*δ*_H_ 1.16) and two olefinic protons (*δ*_H_ 5.29 and 6.07). Its ^13^C NMR and DEPT spectra revealed 30 carbon resonances (Table 2), including six methyls, nine methylenes (one hydroxymethyl carbon), seven methines (two olefinic carbons), and eight quaternary carbons (one carboxyl, one carbonyl and two olefinic carbons). Subtracting the four degrees of unsaturation generated by carboxyl, carbonyl, and olefinic groups, the remaining four degrees of unsaturation manifested that **1** had a tetracyclic structure.

**Table 1:** ^1^H NMR data for compounds **1**–**4** in pyridine-*d*_5_ at 500 MHz (*δ* in ppm, *J* in Hz)

No.	**1**	**2**	**3**	**4**
1a	1.92 (overlap)	2.00 (overlap)	2.40 (overlap)	1.86 (m)
1b	1.79 (overlap)	1.78 (overlap)	1.59 (overlap)	1.64 (overlap)
2a	2.79 (overlap)	2.65 (m)	2.62 (overlap)	1.97 (overlap)
2b	2.49 (m)	2.42 (m)	2.42 (overlap)	1.97 (overlap)
3				3.49 (m)
5	1.86 (overlap)	1.54 (overlap)	2.61 (overlap)	0.97 (dd, 12.1, 2.0)
6a	1.53 (m)	2.06 (overlap)	1.61 (overlap)	1.75 (overlap)
6b	1.44 (m)	1.56 (overlap)	1.33 (overlap)	1.51 (m)
7a	1.79 (overlap)	2.77 (overlap)	1.97 (dd, 12.2, 4.0)	1.75 (overlap)
7b	1.04 (overlap)	1.80 (overlap)	1.57 (overlap)	1.39 (overlap)
8	2.26 (br d, 12.9)		1.52 (dd, 12.7, 3.4)	2.48(m)
11	5.29 (s)	5.94 (s)	5.14 (s)	5.56 (s)
12	2.98 (s)			2.61 (br s)
15a	1.67 (overlap)	4.33 (s)	1.61 (overlap)	1.77 (overlap)
15b	1.67 (overlap)		1.21 (overlap)	1.43 (overlap)
16a	2.10 (overlap)	4.70 (s)	1.80 (overlap)	1.77 (overlap)
16b	1.57 (overlap)		1.80 (overlap)	1.77 (overlap)
17	2.03 (q, 5.8)		2.57 (br s)	1.64 (overlap)
18a	4.16 (2H, m)	2.09 (3H, s)	5.04 (d, 12.1)	1.35 (3H, s)
18b			4.75 (d, 12.1)	
19	1.25 (3H, s)	1.48 (3H, s)	1.03 (3H, s)	1.20 (3H, s)
20	2.16 (overlap)	2.81 (overlap)	2.30 (br, s)	2.42 (br s)
21	1.16 (3H, d, 7.6)	0.92 (3H, d, 6.8)	1.11 (3H, d, 6.7)	1.19 (3H, d, 7.1)
22a	1.96 (overlap)	1.85 (overlap)	1.70 (m)	1.91 (overlap)
22b	1.24 (overlap)	1.63 (br s)	1.32 (overlap)	1.31 (overlap)
23a	2.80 (overlap)	3.05 (dq, 15.6, 7.9)	2.81 (m)	2.87 (overlap)
23b	2.80 (overlap)	2.81 (overlap)	1.28 (overlap)	2.87 (overlap)
24	6.07 (t, 7.5)	6.15 (t, 6.9)	6.12 (t, 7.5)	6.13 (t, 7.4)
27	2.14 (3H, s)	2.17 (3H, s)	2.12 (3H, s)	2.15 (3H, s)
28	1.15 (3H, s)	1.73 (3H, s)	1.37 (3H, s)	1.10 (3H, s)
29	1.39 (3H, s)	1.15 (3H, s)	1.20 (3H, s)	1.26 (3H, s)
30	1.06 (3H, s)	1.06 (3H, s)	1.03 (3H, s)	1.12 (3H, s)

**Table 2:** ^13^C NMR data for compounds **1**–**4** at 125 MHz in pyridine-*d*_5_ (*δ* in ppm)

No.	**1**	**2**	**3**	**4**	No.	**1**	**2**	**3**	**4**
1	36.0 t	36.3 t	31.4 t	36.2 t	16	19.3 t	80.0 d	18.8 t	27.2 t
2	35.4 t	34.7 t	34.9 t	28.6 t	17	49.7 d	136.8 s	41.1 d	53.4 d
3	215.6 s	215.6 s	216.0 s	78.3 d	18	68.0 t	12.3 q	61.0 t	22.8 q
4	48.3 s	47.7 s	47.9 s	39.7 s	19	18.4 q	21.0 q	21.4 q	21.1 q
5	55.0 d	53.0 d	47.1 d	53.3 d	20	32.1 d	34.2 d	34.9 d	31.4 d
6	23.5 t	20.3 t	22.8 t	21.8 t	21	21.3 q	19.1 q	19.1 q	21.8 q
7	29.7 t	35.2 t	21.2 t	21.4 t	22	34.7 t	35.2 t	32.8 t	34.0 t
8	54.7 d	92.8 s	51.5 d	49.0 d	23	29.3 t	28.5 t	29.0 t	29.0 t
9	154.4 s	153.3 s	80.5 s	156.0 s	24	142.9 d	142.6 d	142.5 d	142.6 d
10	38.1 s	37.5 s	41.9 s	38.3 s	25	129.0 s	128.9 s	128.9 s	128.7 s
11	118.8 d	130.4 d	70.1 d	119.2 d	26	170.9 s	170.7 s	170.5 s	170.7 s
12	58.6 d	88.2 s	148.9 s	66.0 d	27	21.9 q	21.6 q	21.6 q	21.6 q
13	77.0 s	137.7 s	137.7 s	76.7 s	28	26.0 q	13.7 q	21.2 q	29.2 q
14	41.7 s	59.6 s	41.5 s	43.5 s	29	26.5 q	26.6 q	26.3 q	29.1 q
15	36.4 t	78.1 d	35.5 t	35.3 t	30	22.0 q	21.9 q	16.3 q	16.7 q

**Fig. 2:**
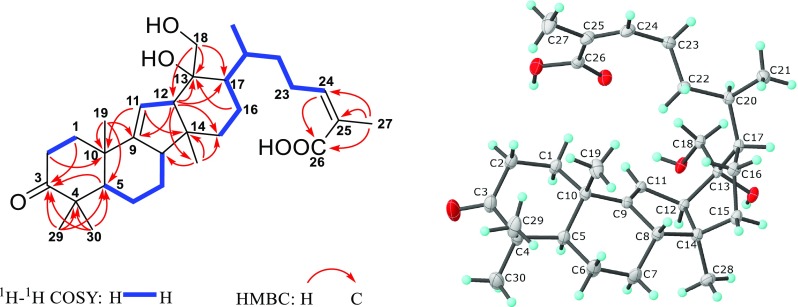
Key ^1^H-^1^H COSY and HMBC correlations and X-ray crystallographic structure of **1**

Detailed analysis of the 1D and 2D NMR spectra of **1** demonstrated its structure to be similar to those of kadcoccine acids A–N [8], which had 14(13 → 12)-abeolanostane scaffolds featuring 6/6/5/6-fused ring systems. The planar structure of **1** could be confirmed by its ^1^H-^1^H COSY and HMBC data (Fig. 2). The HMBC correlations from H_2_-1, H-5, H_3_-29, and H_3_-30 to C-3 (*δ*_C_ 215.6) implied the carbonylation of C-3; The HMBC correlations from H-12 and H_2_-16 to C-13 (*δ*_C_ 77.0), in combination with the HMBC correlations from H_2_-18 (*δ*_H_ 4.16) to C-12, C-13 and C-17 ascertained the substitution of a hydroxyl group at C-13 and C-18, respectively. The HMBC correlations from H_3_-27 to C-24 (*δ*_C_ 142.9), C-25 (*δ*_C_ 129.0) and C-26 (*δ*_C_ 170.9), and from H-24 (*δ*_H_ 6.07) to C-26 implied the existence of the C-24/C-25 double bond and carboxyl group at C-26. The COSY correlation of H-11 (*δ*_H_ 5.29)/H-12 and the HMBC correlations from H-12 and H_3_-19 to C-9 (*δ*_C_ 154.4) suggested the presence of *Δ*^9,11^ double bond. By analyzing its ROESY spectrum, the cross-peak of H-24/H_3_-27 indicated that the *Δ*^24,25^ double bond took *Z*-geometry. However, the configuration of C-13 couldn’t be determined due to the lack of HO-13 signal in the ^1^H NMR spectrum. Fortunately, the crystals of **1** were obtained and subjected to the X-ray diffraction analysis through CuK*α* radiation, which determined its absolute configuration to be 5*R*,8*S*,10*S*,12*R*,13*R*,14*S*,17*R*, and 20*R* (Flack parameter = 0.06(2)) (CCDC:1903695) (Fig. 2).

Kadcoccitane B (**2**) was obtained as white amorphous powder. The molecular formula C_30_H_42_O_6_ was determined by HRESIMS ([M + Na]^+^ *m*/*z* 521.2872, calcd 521.2874), corresponding to ten indices of hydrogen deficiency. The ^1^H-NMR spectrum showed proton signals which could be ascribed to six singlet methyls (*δ*_H_ 1.06, 1.15, 1.48, 1.73, 2.09 and 2.17), one doublet methyl (*δ*_H_ 0.92) and two olefinic protons (*δ*_H_ 5.94 and 6.15) (Table 1). Moreover, 30 carbon resonances (Table 2), including seven methyls, six methylenes, six methines (two olefinic carbons and two oxygen-bearing methines), and eleven quaternary carbons (one carboxyl, one carbonyl and four olefinic carbons) could be observed from its ^13^C NMR and DEPT spectra. All the aforementioned carboxyl, carbonyl and olefinic groups accounted for four out of nine degrees of unsaturation, indicative of a pentacyclic structure for **2**.

**Fig. 3:**
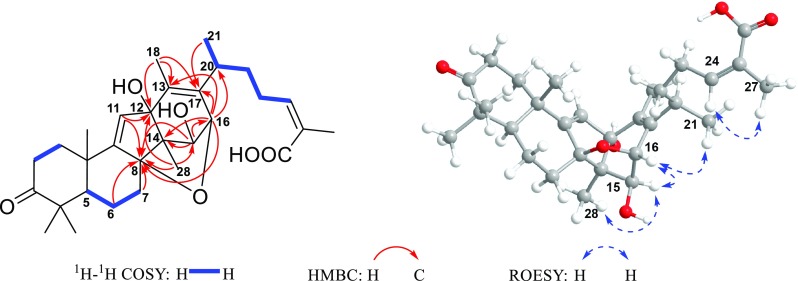
Key ^1^H-^1^H COSY, HMBC and ROESY correlations of **2**

Careful analysis of the HMBC and ^1^H-^1^H COSY spectra (Fig. 3) of **2** demonstrated that it shared the same structure of side chain (C-20–C-27) as that of compound **1,** as well as a similar A/B ring system except that C-8 (*δ*_C_ 92.8) was oxygenated, as revealed by the ^1^H-^1^H COSY correlations of H-5/H_2_-6/H_2_-7, together with the HMBC correlations from H_2_-6 and H_2_-7 to C-8. Additionally, the HMBC correlations from olefinic H-11 (*δ*_H_ 5.94) to C-12 (*δ*_C_ 88.2), from H_3_-28 to C-15 (*δ*_C_ 78.1), and from H-15 (*δ*_H_ 4.33) to C-16 (*δ*_C_ 80.0) demonstrated that C-12, C-15, and C-16 were all oxygenated. Besides, the correlations from H_3_-18 and H-16 (*δ*_H_ 4.70) to C-13 (*δ*_C_ 137.7) and C-17 (*δ*_C_ 136.8) suggested the presence of the C-13/C-17 double bond. Most importantly, the correlation of H-16/C-8, the intensity of which was nearly as strong as that of the H-15/C-8 correlation, was observed in the HMBC spectrum. Hence, in consideration of the 4-bond distance between H-16 and C-8 if following the H-16/C-16/C-15/C-14/C-8 path, an epoxy was tentatively constructed between C-8 and C-16.

**Fig. 4:**
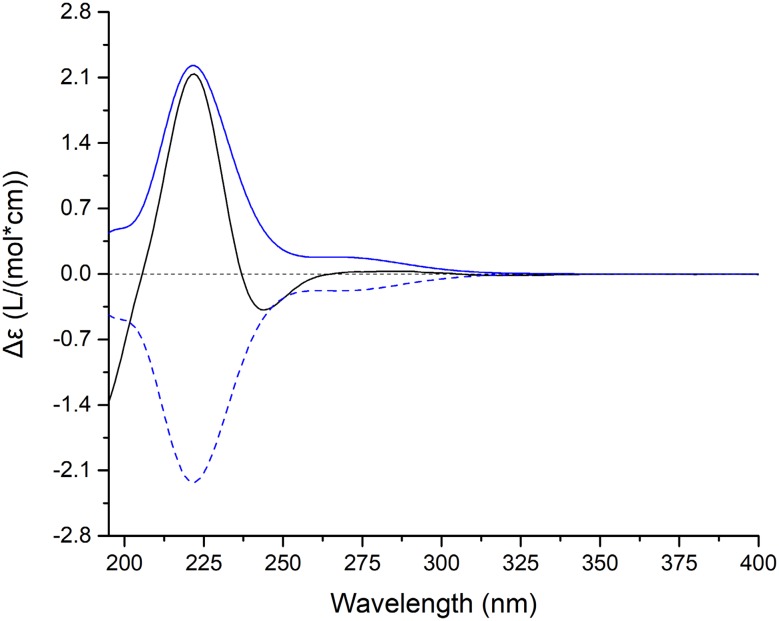
Experimental ECD spectrum of **2** (black); Calculated ECD spectra of (5*R*,8*S*,10*S*,12*R*,14*R*,15*S*,16*R*,20*S*)-**2b** (shift = − 7 nm, blue) and its enantiomer (shift = − 7 nm, blue dash)

As for the stereochemistry of **2**, the H-15/H_3_-28*α* correlation in the ROESY spectrum of **2** (Fig. 3) indicated that H-15 adopted *α*-orientation. Moreover, the fact that H-15 and H-16 both existed as singlets in the ^1^H-NMR spectrum, together with the absence of the ^1^H-^1^H COSY correlation of H-15/H-16, suggested that the dihedral angle of H-15/C-15/C-16/H-16 was around 90°, thus demanding H-16 to be *α*-oriented. Noteworthily, though there existed two possibilities for the orientation of HO-12 theoretically, the rigidity of the C/D ring system denied the existence of the HO-12*β* isomer. Moreover, the stereochemistry of C-20 couldn’t be determined presently since it was located on the flexible side chain. Thus, the two possible C-20 stereoisomers of **2**, (5R*,8S*,10S*,12R*,14R*,15S*,16R*,20R*)-**2** (**2a**) and (5R*,8S*,10S*,12R*,14R*,15S*,16R*,20S*)-**2** (**2b**) (Fig. S71) were subjected to quantum calculations of NMR chemical shifts at MPW1PW91-SCRF/6-31 + G(d,p)//M06-2X-D3/def2-SVP level of theory in chloroform with IEFPCM solvent model (Tables S2 and S3). As a result, the calculated NMR shifts of **2b** were found to be in better agreement with their experimental counterparts, as indicated by parameters including R^2^, MAE, CMAE, as well as the DP4 + probability (Table 3). Then, quantum chemical calculation of spin–spin coupling constants (SSCC) of **2b** was run at B97-2/pcJ-1 level with IEFPCM solvent model in chloroform (Table S5), the theoretical SSCC of ^3^*J*_H-15/H-16_ (0.5 Hz) could clearly explain the aforementioned splitting pattern of H-15 and H-16 in the ^1^H NMR spectrum, while the predicted SSCCs of ^3^*J*_H-16/C-8_ (7.0) could well account for the strong H-16/C-8 correlation in the HMBC spectrum. Thus, the planar structure as well as the relative configuration of **2** was established as depicted in Fig. 1, and subsequent TDDFT calculation at CAM-B3LYP-SCRF/def2-SVP level of theory in methanol with IEFPCM solvent model succeeded in establishing the absolute configuration of **2** as 5*R*, 8*S*, 10*S*, 12*R*, 14*R*, 15*S*, 16*R* and 20*S* (Fig. 4). Accordingly, kadcoccitane B (**2**) represented the first example of 8,16-epoxy-14(13 → 12)-abeolanostane triterpenoid featuring an unusual 20*S* configuration.

**Table 3:** Comparison of the key parameters of **2a** and **2b** in NMR computation

Parameters	**2a**	**2b**
R^2^ (^13^C)	0.9983	0.9984
MAE (^13^C)	2.4 ppm	2.2 ppm
CMAE (^13^C)	1.7 ppm	1.5 ppm
R^2^ (^1^H)	0.9633	0.9845
MAE (^1^H)	0.23 ppm	0.14 ppm
CMAE (^1^H)	0.21 ppm	0.13 ppm
DP4 + (^1^H data)	0.00%	100.00%
DP4 + (^13^C data)	1.26%	98.74%
DP4 + (all data)	0.00%	100.00%

Kadcoccitane C (**3**), white amorphous powder, possessed a molecular formula of C_30_H_46_O_6_ supported by the HRESIMS ([M – H]^−^ *m/z* 501.3214, calcd 501.3222), with eight indices of hydrogen deficiency. Five singlet methyls (*δ*_H_ 1.03, 1.03, 1.20, 1.37 and 2.12), one doublet methyl (*δ*_H_ 1.11) and one olefinic proton (*δ*_H_ 6.12) were observed from its ^1^H NMR spectrum (Table 1). The ^13^C NMR combined with DEPT spectra displayed 30 carbon signals (Table 2) which comprised six methyls, nine methylenes (one hydroxymethyl carbon), six methines (one olefinic and one oxygen-bearing carbons), and nine quaternary carbons (one carboxyl, one carbonyl and three olefinic carbons). Judging from the total degrees of unsaturation and those which was occupied by carbon–carbon and carbon–oxygen double bonds, a tetracyclic scaffold of **3** was conjectured to exist.

**Fig. 5:**
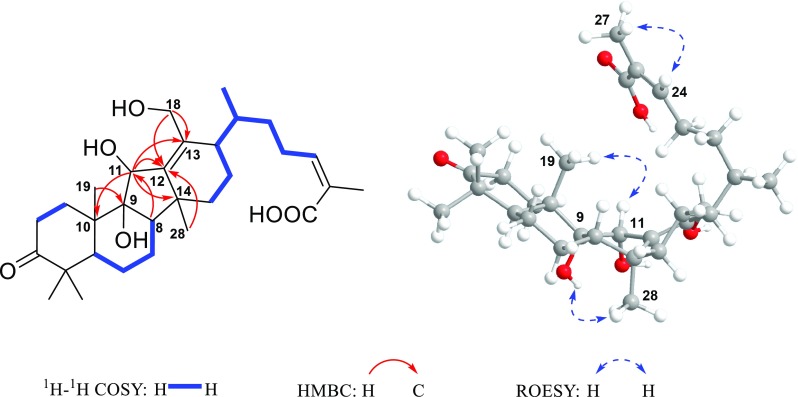
Key ^1^H-^1^H COSY, HMBC and ROESY correlations of compound **3**

In comparison with **1**, two variations of compound **3** could be observed. One was that the double bond of C-9/C-11 of **3** was oxidized to a vicinal diol, which could be determined by the HMBC correlations from H_3_-19 to C-9 (*δ*_C_ 80.5), from H-11 (*δ*_H_ 5.14) to C-10 and C-14, and from H-8 to C-11 (*δ*_C_ 70.1) (Fig. 5). The other was that *Δ*^12,13^ double band was formed by the dehydration at C-13, which could be ascertained by the HMBC correlations from H-11, H_2_-18 and H_3_-28 to C-12 (*δ*_C_ 148.9), and from H-11 and H_2_-18 to C-13 (*δ*_C_ 137.7) (Fig. 5). In the ROESY spectrum (Fig. 5), the cross peaks of HO-9/H_3_-28*α* and H-11/H_3_-19*β* suggested the *α*-orientation of HO-9 and HO-11, respectively.

Kadcoccitane D (**4**) was isolated as white amorphous powder. Its HRESIMS data ([M + Na]^+^ *m/z* 495.3439, calcd 495.3445) indicated that it possessed a molecular formula of C_30_H_48_O_4_, suggesting seven degrees of unsaturation. The ^1^H NMR spectrum showed resonances for six singlet methyls (*δ*_H_ 1.12, 1.10, 1.20, 1.26, 1.35 and 2.15), one doublet methyl (*δ*_H_ 1.19) and two olefinic protons (*δ*_H_ 5.56 and 6.13) (Table 1). The ^13^C NMR and DEPT spectra showed 30 carbon resonances (Table 2) including seven methyls, eight methylenes, eight methines (two olefinic and one oxygen-bearing carbons), and seven quaternary carbons (one carboxyl, and two olefinic carbons). Considering the three degrees of unsaturation generated by carboxyl and olefinic groups, compound **4** was a tetracyclic triterpenoid.

**Fig. 6:**
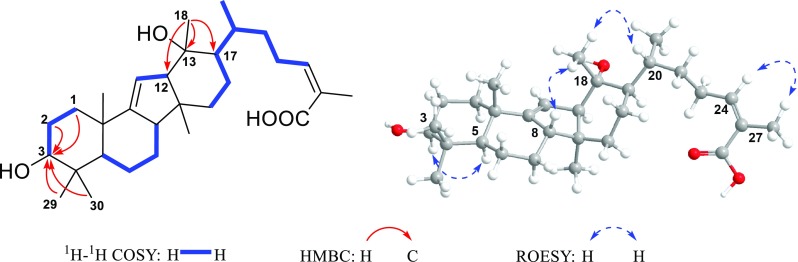
Key ^1^H-^1^H COSY, HMBC and ROESY correlations of compound **4**

The structure of **4** also differed from that of **1** in two aspects. Firstly, the carbonyl at C-3 of compound **4** was reduced to hydroxy, which could be determined by the HMBC correlations from H_2_-1, H_2_-2, H_3_-29 and H_3_-30 to C-3 (*δ*_C_ 78.3) (Fig. 6). Moreover, the HMBC correlations from H_3_-18 to C-12, C-13 (*δ*_C_ 76.7) and C-17 manifested that the hydroxymethyl of C-18 was replaced by a methyl (Fig. 6). The ROESY correlations of H-3/H-5*α* and H-8*β*/H-18/H-20*β* demonstrated the spatial *α*-orientation of H-3 and *β*-orientation of H_3_-18 (Fig. 6).

The phytochemical investigations on *K. coccinea* indicated that the secondary metabolites from the roots generally possess higher degrees of oxidation than those obtained from its stems, which may be mainly attributed to the abundant oxidases existing in the roots. However, to arrive at more definite conclusion, more in-depth research still needs to be undertaken. In addition, compounds **1**, **3** and **4** were screened for the bioactivity against platelet aggregation induced by colloid. The result suggested that the inhibition ratios of **3** and **4** were 12.4 ± 12.5% and 19.4 ± 14.4% (*p *< 0.05), respectively, under the concentration of 100 *µ*M. Compounds **1** and **4** were tested for anticoagulant activity. The result showed that their IC_50_ values were 37.8 and 31.5 *µ*M, respectively.

## Experimental

### General Experimental Procedures

1D and 2D NMR spectra were recorded on Bruker AV III 500 MHz spectrometer using TMS as the internal standard. Chemical shifts (*δ*) are expressed in ppm. HRESIMS was performed on an API QSTAR Pulsar i spectrometer. Melting point was recorded on an RDY-1B micro melting point apparatus. UV spectra were obtained on a Shimadzu UV-2401PC spectrophotometer. Optical rotations were measured in MeOH with a JASCO P-1020 polarimeter or an Autopol VI, Serial #91058. IR spectra were obtained on a Bruker Tensor-27 FT-IR spectrometer using KBr pellets. Column chromatography (CC) was performed with silica gel (80–100 or 100–200 mesh; Qingdao Marine Chemical, Inc., Qingdao, People´s Republic of China). Analytical HPLC was performed on an Agilent 1260 liquid chromatograph with a Zorbax SB-C18 (4.6 mm × 250 mm) column. Semi-preparative HPLC was performed on an Agilent 1200 liquid chromatograph with a Zorbax SB-C18 (9.4 mm × 250 mm) column or a COSMOSIL Cholester (10ID × 250 mm) column. Fractions were monitored by thin layer chromatography, spots were visualized by UV light (254 nm and 365 nm) and by heating silica gel plates sprayed with 10% H_2_SO_4_ in EtOH. All solvents used in column chromatography were distilled.

### Plant Material

The roots of *K. coccinea* (Lem.) A. C. Smith were collected from Jingzhou Miao and Dong Autonomous County in Hunan Province, People’s Republic of China, in June 2016 and identified by Prof. Heng Li at Kunming Institute of Botany. A voucher specimen (KIB 2016062101) has been deposited in State Key Laboratory of Phytochemistry and Plant Resources in West China, Kunming Institute of Botany, Chinese Academy of Sciences.

### Extraction and Isolation

The air-dried and powdered roots (15.0 Kg) of *K. coccinea* were soaked with industrial alcohol (25 L) for four times, 3 days each time at room temperature. The total extracting solution was sequentially condensed to a certain extent under the condition of heating (40 °C) and reduced pressure in vacuo. Then the concentrate was suspended in water (1/1, v/v). The remaining alcohol was continuously evaporated under the same condition. After that, the sample was extracted with EtOAc and n-BuOH (1/2, v/v) for four times, respectively.

The EtOAc extract (1.8 Kg) was subjected to a silica gel column chromatography eluted with CHCl_3_/Me_2_CO gradiently (1:0 to 0:1) to obtain seven fractions A–G Fraction D (68 g) was chromatographed by RP-C_18_ using a gradient mobile phase of MeOH/H_2_O (30:70 to 90:0) to obtain fractions D1–D7.

Fr. D5 (8 g) was chromatographed by silica gel with a gradient elution of PE/Me_2_CO (1:0–0:1) to afford fractions D51–D55. Fr. D53 (1 g) was subjected to Sephadex LH-20 chromatography using MeOH as mobile phase to give fractions D531–D535. Crystals (compound **1**, 50 mg) were separated from the solution of Fr. D532 (180 mg). Fr. D533 (230 mg) underwent preparative HPLC (MeOH/H_2_O, 15 mL/min) and multiple semi-preparative HPLC (MeCN/H_2_O or MeOH/H_2_O, 3 mL/min) to yield compound **2** (9.5 mg).

Fraction D6 (10 g) was chromatographed by silica gel with a gradient elution of PE/Me_2_CO (1:0–0:1) to afford fractions D61–D65. Fr. D64 (1.5 g) was subjected to Sephadex LH-20 chromatography eluted by MeOH and then purified by repeated semi-preparative HPLC (MeCN/H_2_O or MeOH/H_2_O, 3 mL/min) to yield compound **3** (3.3 mg). Fr. D65 (980 mg) was chromatographed by Sephadex LH-20 eluted with MeOH to give fractions D651–D655. Fr. 653 (62 mg) was further purified by repeated semi-preparative HPLC (MeCN/H_2_O or MeOH/H_2_O, 3 mL/min) to yield compound **4** (7.4 mg).

### Characteristic Data of Compounds **1**–**4**

Kadcoccitane A (**1**): Colorless acicular crystals, melting point: 213 °C. $\left[ \alpha \right]_{\text{D}}^{25} - 58.4\,({{c}}\,0.214,\,{\text{MeOH}})$. UV (MeOH) *λ*_max_ (log *ε*) 206 (3.9) nm. IR (KBr) *ν*_max_ 3440, 2936, 1703, 1638, 1421, 1241, 1035, 886, 647 cm^−1^. CD (MeOH): 205 nm (*Δ*ε = − 0.75), 215 nm (*Δ*ε = − 1.03). Positive ESIMS *m*/*z* 509 [M + Na]^+^. ^1^H and ^13^C NMR data in pyridine-*d*_5_, see Tables 1 and 2. ^1^H and ^13^C NMR data in CDCl_3_, see Table S1.

**Crystal data for 1** C_30_H_46_O_5_, *M* = 486.67, *a* = 10.6452(2) Å, *b* = 27.2411(5) Å, *c* = 28.4264(5) Å, *α* = 90°, *β* = 90°, *γ* = 90°, *V* = 8243.3(3) Å^3^, *T* = 100(2) K, space group *P*212121, *Z* = 12, *μ*(CuK*α*) = 0.618 mm^−1^, 49684 reflections measured, 14281 independent reflections (*R*_*int*_ = 0.0301). The final *R*_1_ values were 0.0336 (*I* > 2*σ*(*I*)). The final *wR*(*F*^2^) values were 0.0874 (*I* > 2*σ*(*I*)). The final *R*_1_ values were 0.0338 (all data). The final *wR*(*F*^2^) values were 0.0876 (all data). The goodness of fit on *F*^2^ was 1.087. Flack parameter = 0.06(2).

Kadcoccitane B (**2**): White amorphous powder. $\left[ \alpha \right]_{\text{D}}^{20} \, - \,44.5\,({c}\,0.163,\,{\text{MeOH}})$. UV (MeOH) *λ*_max_ (log *ε*) 216 (4.1) nm, 196 (4.1) nm, 206 (4.0) nm. IR (KBr) *ν*_max_ 3445, 2967, 2934, 1693, 1640, 1458, 1383, 1048, 581 cm^−1^. CD (MeOH): 222 nm (*Δ*ε = 2.14), 244 nm (*Δ*ε = − 0.38). Positive ESIMS *m*/*z* 521 [M + Na]^+^. ^1^H and ^13^C NMR data in pyridine-*d*_5_, see Tables 1 and 2.

Kadcoccitane C (**3**): White amorphous powder. $\left[ \alpha \right]_{\text{D}}^{25} \, - \,37.7\,({c}\,0.107,\,{\text{MeOH}})$. UV (MeOH) *λ*_max_ (log *ε*) 207 (4.0) nm. IR (KBr) *ν*_max_ 3432, 2929, 1701, 1638, 1457, 1383, 1264, 1043, 893, 582 cm^−1^. CD (MeOH): 196 nm (*Δ*ε = − 1.48), 207 nm (*Δ*ε = + 0.25), 225 nm (*Δ*ε = − 0.41). Negative ESIMS *m*/*z* 501 [M–H]^−^. ^1^H and ^13^C NMR data in pyridine-*d*_5_, see Tables 1 and 2.

Kadcoccitane D (**4**): White amorphous powder. $\left[ \alpha \right]_{\text{D}}^{25} \, - \,40.3\,({c}\,0.120,\,{\text{MeOH}})$. UV (MeOH) *λ*_max_ (log *ε*) 208 (4.0) nm. IR (KBr) *ν*_max_ 3433, 2936, 1695, 1638, 1458, 1337, 1252, 1037, 859, 580 cm^−1^. CD (MeOH): 208 nm (*Δ*ε = 0.15), 222 nm (*Δ*ε = − 0.25). Positive ESIMS *m*/*z* 495 [M + Na]^+^. ^1^H and ^13^C NMR data in pyridine-*d*_5_, see Tables 1 and 2.

### Computational Method

_ENREF_1Conformational analysis of **2a** and **2b** was initially performed in Spartan’16 (Wavenfunction, Irvine, CA, USA, 2016) using the Monte Carlo algorithm and Merck molecular force field (MMFF). To avoid losing relevant conformations during the conformational search stage, the “set torsions” function was used to give all rotatable bonds a six fold sampling, as well as to allow the atoms on the aliphatic ring to flip. Maximum 20000 conformers were examined for each diastereoisomer, and those obtained conformers within 20 kcal/mol were kept (1000 ones for each isomer).

These conformers were subjected to semiempirical geometry optimization using the GFN2-xTB method [12] implemented in the XTB code (version 6.1) in order to obtain conformers better correlating with DFT calculations. Subsequently, XTB geometries with a difference of distance geometry within 0.25 were clustered. Then, clustered XTB geometries within an energy window of 8 kcal/mol were subjected to a DFT energy calculation at M06-2X/def2-SVP level of theory with DFT-D3 dispersion correction [13] using Gaussian 09 program [14], and those conformers within an energy window of 5 kcal/mol were kept. The completion of step was hugely aided by the Molclus program [15] (and its “isostat” module).

The above screened conformers were subjected to DFT geometry optimization at M06-2X-D3/def2-SVP level of theory. Frequency analyses of all optimized conformers were undertaken at the same level of theory to ensure they were true local minima on the potential energy surface. Then, energies of all optimized conformers were evaluated at M06-2X-D3/def2-TZVP level of theory. Gibbs free energies of each conformers were calculated by adding “Thermal correction to Gibbs Free Energy” obtained by frequency analysis to electronic energies obtained at M06-2X-D3/def2-TZVP level of theory. Room-temperature (298.15 K) equilibrium populations were calculated according to Boltzmann distribution law:

$$p_{i} \, = \,\frac{{n_{i} }}{{\mathop \sum \nolimits_{j} n_{i} }}\, = \, \frac{{e^{{ - \Delta G_{i} /RT}} }}{{\mathop \sum \nolimits_{j} e^{{ - \Delta G_{j} /RT}} }}\, = \, \frac{{Q_{i(Relative)} }}{{Q_{(Relative)} }}$$

where *P*_*i*_ is the population of the *i*th conformer; *n*_*i*_ the number of molecules in *i*th conformer; Δ*G* is the relative Gibbs free energy (kcal/mol); *T* is the temperature, usually the room temperature (298.15 K); *R* is the ideal gas constant (0.0019858995); *Q* is the partition function. Those conformers accounting for over 98% population were subjected to subsequent calculations.

NMR shielding constants were calculated with the GIAO method at mPW1PW91-SCRF/6-31 + G(d,p) level with IEFPCM solvent model in chloroform solvent. The shielding constants obtained were converted into chemical shifts by referencing to TMS at 0 ppm (*δ*_cal_ = *σ*_TMS_ − *σ*_cal_), where the *σ*_TMS_ was the shielding constant of TMS calculated at the same level. For each possible candidate, the parameters *a* and *b* of the linear regression *δ*_cal_ = *aδ*_exp_ + *b*; the correlation coefficient, *R*^2^; the mean absolute error (MAE) defined as Σ_n_ |*δ*_cal_ − *δ*_exp_|/*n*; the corrected mean absolute error, CMAE, defined as Σ_n_ |*δ*_corr_ − *δ*_exp_|/*n*, where *δ*_corr_ = (*δ*_cal_ − *b*)/*a*, were calculated [16, 17]. The DP4 + probabilities of each possible candidate were calculated with the EXCEL spreadsheet provided by Sarotti et al. [18].

Spin–spin coupling constants were calculated at B97-2/pcJ-1 [19] level with IEFPCM solvent model in chloroform.

TDDFT ECD calculations were run at CAM-B3LYP/def2-SVP level of theory in MeOH with IEFPCM solvent model. For each conformer, 30 excited states were calculated. The calculated ECD curves were generated using Multiwfn 3.6 software [20].

### Activity Screening

#### Anti-platelet Aggregation Induced by Colloid

The experimental blood was extracted from the ears of Japanese white rabbits and conserved in vacuum blood-collection tubes with sodium citrate (whole blood: sodium citrate = 9:1). The blood-collection tubes were turned upside down gently to ensure homogeneous mixing of blood and anticoagulant and then centrifuged (200×*g*, 10 min). The supernatant was thus collected as platelet rich plasma (PRP). The residual blood was sequentially centrifuged (2400×*g*, 20 min) and supernatant was collected as platelet poor plasma (PPP). The platelet count of PRP was adjusted to 500 × 10^9^ L^−1^ based on PPP [21–23]. The test samples were weighed accurately and dissolved with DMSO to 10 mM. The reference substance, Ticagrelor, was prepared to 0.5 mg/mL with DMSO. The colloid and ADP were prepared to 1 mg/mL and 1 mmol/L, respectively, and conserved at specified temperature according to the instruction.

Two cuvettes, one with stir bar and the other without stir bar, were put in the heater of the platelet aggregation apparatus, heated at 37 °C for 10 min. Then 250 *μ*L of PRP and 2.5 *μ*L of test sample were added into the cuvette with stir bar. 250 *μ*L of PPP and 2.5 *μ*L of DMSO were added into the cuvette without stir bar. After 5 min of heating, the two cuvettes were put at the test positions of PRP and PPP, respectively. After adjusting the baseline of recording curve, the inducer (1 *μ*L of colloid) was added into the cuvettes. The curve of platelet aggregation was recorded and the maximum aggregation rate were accordingly calculated. The inhibition rate of the test samples against rabbit platelet aggregation induced by colloid could also be calculated. The formula is as follows: R = (A − B) * 100%/A. R: Inhibition rate, A: The maximum aggregation of solvent, B: The maximum aggregation of test sample (or reference substance).

#### Anticoagulant Activity

The test compounds were diluted with DMSO to 10 mM and then diluted with 0.02 M Tris-HCl (pH 7.4) with 5% Tween 80 to 1 Mm. The positive control was Low Molecular Weight Heparin (LMWH) and the blank control was 0.02 M Tris-HCl (pH 7.4) with 5% Tween 80 and 10% DMSO. The solution of the test sample or reference substance was added into the cuvette preheated at 37 °C and then the control plasma was also added into it. After heating at 37 °C for 2 min, the TT (thrombin time) reagent preheated at 37 °C was also added into it. The clotting time was recorded afterwards.

## Electronic supplementary material

Below is the link to the electronic supplementary material.

Supplementary data associated with this article including 1D and 2D NMR, ESIMS, HRESIMS, UV, IR, CD and OR of **1**–**4**, computational data of **2** are available. Supplementary material 1 (DOCX 13247 kb)
