# Supplementary material for: Four 14(13 → 12)-Abeolanostane Triterpenoids with 6/6/5/6-Fused Ring System from the Roots of Kadsura coccinea
Source: Nat Prod Bioprospect. 2019 Apr 11;9(3):165–73. doi: 10.1007/s13659-019-0203-4 (PMC6538734; doi:10.1007/s13659-019-0203-4)
Supplement: Supplementary file 1 — Supplementary data associated with this article including 1D and 2D NMR, ESIMS, HRESIMS, UV, IR, CD and OR of 1–4, computational data of 2 are available. Supplementary material 1 (DOCX 13247 kb) [file 13659_2019_203_MOESM1_ESM.docx]

**Supporting Information for**

**Four** **14(13→12)-abeolanostane Triterpenoids with 6/6/5/6-Fused Ring System from the Roots of** ***Kadsura coccinea***

Hou-Chao Xu^a,b^ · Kun Hu^a^ · Han-Dong Sun^a^ · Pema-Tenzin Puno*^a^

^a^ State Key Laboratory of Phytochemistry and Plant Resources in West China, Kunming Institute of Botany, Chinese Academy of Sciences, and Yunnan Key Laboratory of Natural Medicinal Chemistry, Kunming 650201, Kunming 650201, People´s Republic of China

^b^ University of Chinese Academy of Sciences, Beijing 100049, People´s Republic of China

*Corresponding author: Tel: 86-0871-65223616 E-mail: [punopematenzin@mail.kib.ac.cn](mailto:punopematenzin@mail.kib.ac.cn)

**Contents**

[**1.** **NMR data of compound 2 in CDCl_3_** 1](#_Toc3839991)

[**2.** **NMR, MS, IR, ECD, OR and UV spectra of compounds 1–4** 2](#_Toc3839992)

[**3.** **Recording curves of influences on the platelet aggregation of rabbits induced by colloid** 67](#_Toc3839993)

[**4.** **Computational data of 2** 70](#_Toc3839994)

[**4.1 General computational data and results of 2** 70](#_Toc3839995)

[**4.2 Computational data of 2a** 73](#_Toc3839996)

[**4.3 Computational data of 2b** 76](#_Toc3839997)

1. **NMR data of compound 2 in CDCl_3_**

**Table S1.** ^1^H and ^13^C NMR spectroscopic data for compound **2** in CDCl_3_(*δ* in ppm, *J* in Hz)

| **NO.** | δ_C_*^b^* | δ_H_*^a^* | **NO.** | δ_C_*^b^* | δ_H_*^a^* |
| --- | --- | --- | --- | --- | --- |
| 1a | 35.9 t | 2.03 (overlap) | 15 | 78.1 d | 3.89 (s) |
| 1b |  | 1.77 (overlap) | 16 | 79.4 d | 4.26 (s) |
| 2a | 34.5 t | 2.65 (ddd, 16.0, 12.4, 7.1) | 17 | 136.5 s |  |
| 2b |  | 2.40 (ddd, 16.0, 6.1, 3.0) | 18 | 11.7 q | 1.70 (3H, s) |
| 3 | 216.8 s |  | 19 | 20.3 q | 1.17 (3H, s) |
| 4 | 47.7 s |  | 20 | 34.0 d | 2.55 (overlap) |
| 5 | 52.6 d | 1.35 (m) | 21 | 18.9 q | 0.95 (3H, d, 6.8) |
| 6a | 19.7 t | 1.81 (overlap) | 22a | 34.3 t | 1.53 (overlap) |
| 6b |  | 1.54 (overlap) | 22b |  | 1.41 (br s) |
| 7a | 36.6 t | 2.06 (overlap) | 23a | 28.2 t | 2.52 (overlap) |
| 7b |  | 1.54 (overlap) | 23b |  | 2.31 (br s) |
| 8 | 92.3 s |  | 24 | 145.9 d | 5.98 (t, 6.8) |
| 9 | 153.7 s |  | 25 | 126.7 s |  |
| 10 | 37.3 s |  | 26 | 172.6 s |  |
| 11 | 129.3 d | 5.56 (s) | 27 | 20.8 q | 1.89 (3H, s) |
| 12 | 88.3 s |  | 28 | 12.2 q | 1.15 (3H, s) |
| 13 | 136.1 s |  | 29 | 22.0 q | 1.06 (3H, s) |
| 14 | 58.6 s |  | 30 | 26.5 q | 1.08 (3H, s) |

*^a^*Recorded at 600 MHz, *^b^*Recorded at 150 MHz.

1. **NMR, MS, IR, ECD, OR and UV spectra of compounds 1–4**


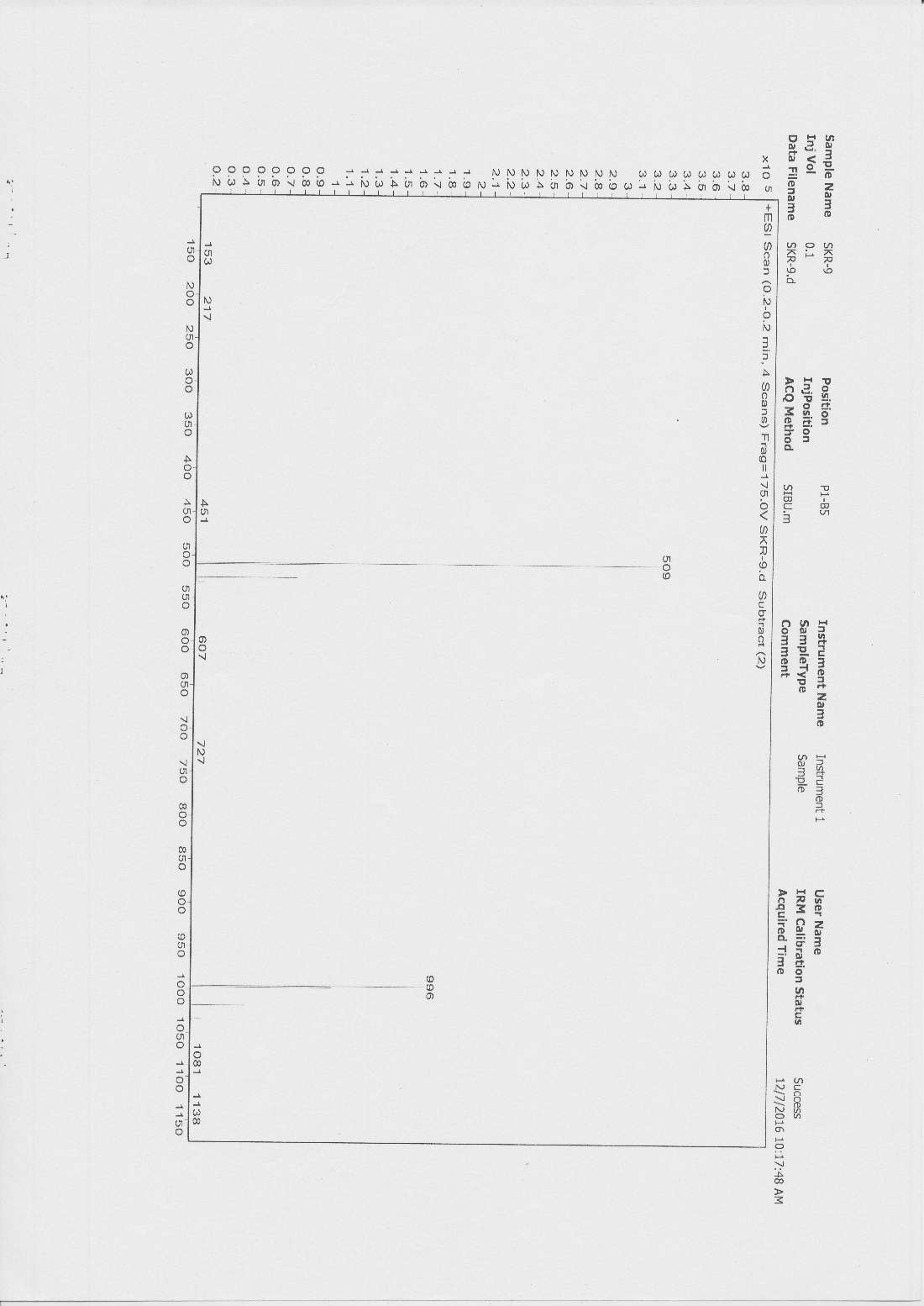


**Figure S1.** ESIMS spectrum of Kadcoccitane A (**1**).

**Figure S2.** HRESIMS spectrum of Kadcoccitane A (**1**)


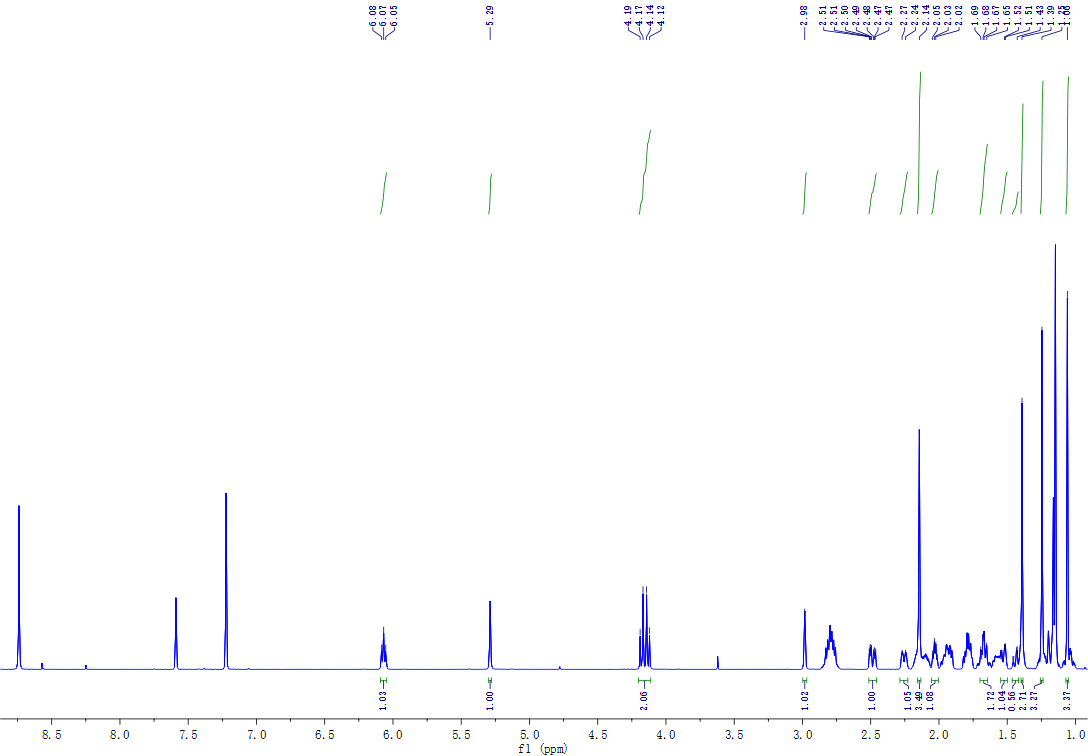


**Figure S3**. ^1^H spectrum of Kadcoccitane A (**1**) in pyridine-*d*_5_.


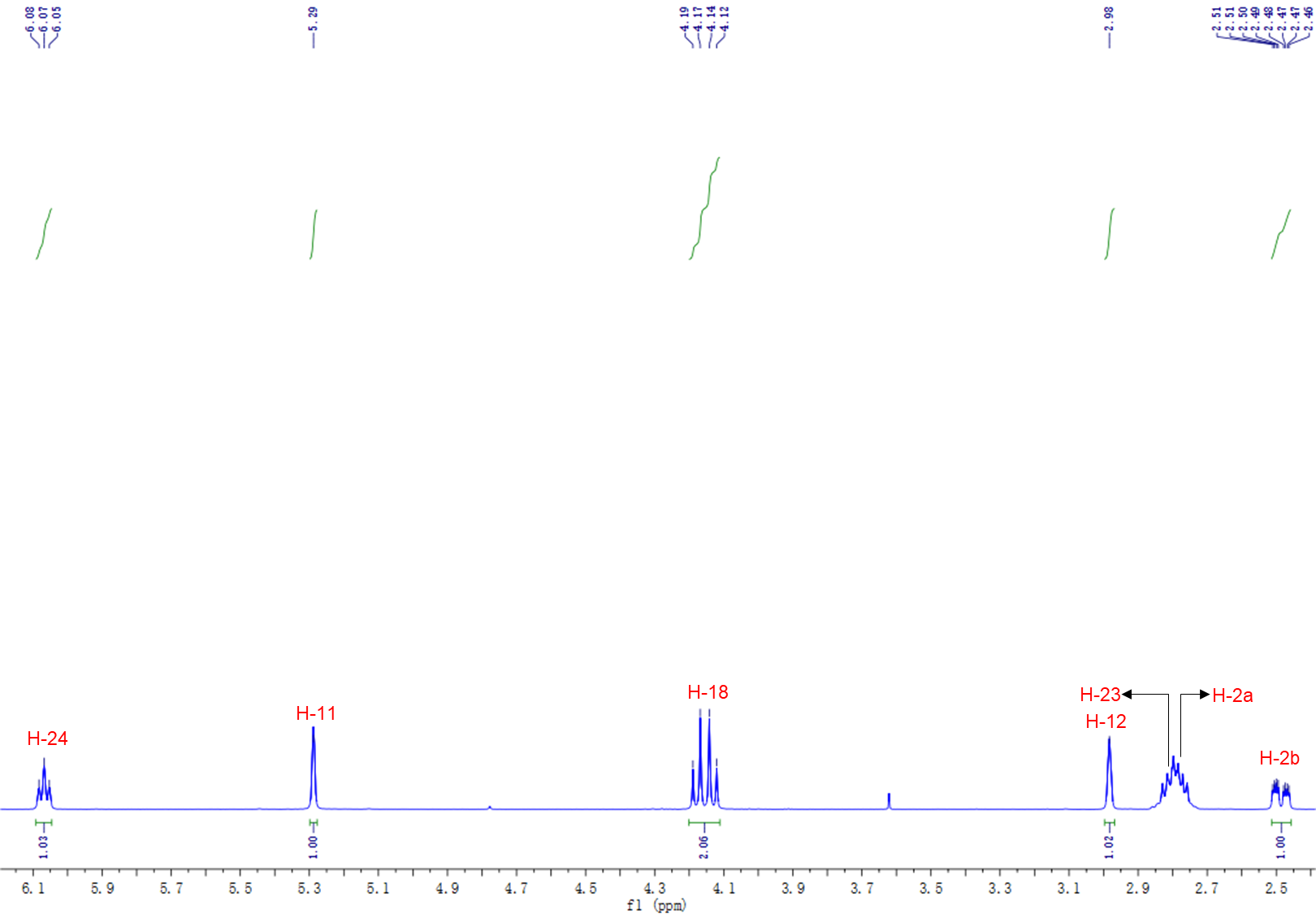


**Figure S4**. Enlarged ^1^H spectrum of Kadcoccitane A (**1**) in pyridine-*d*_5_.


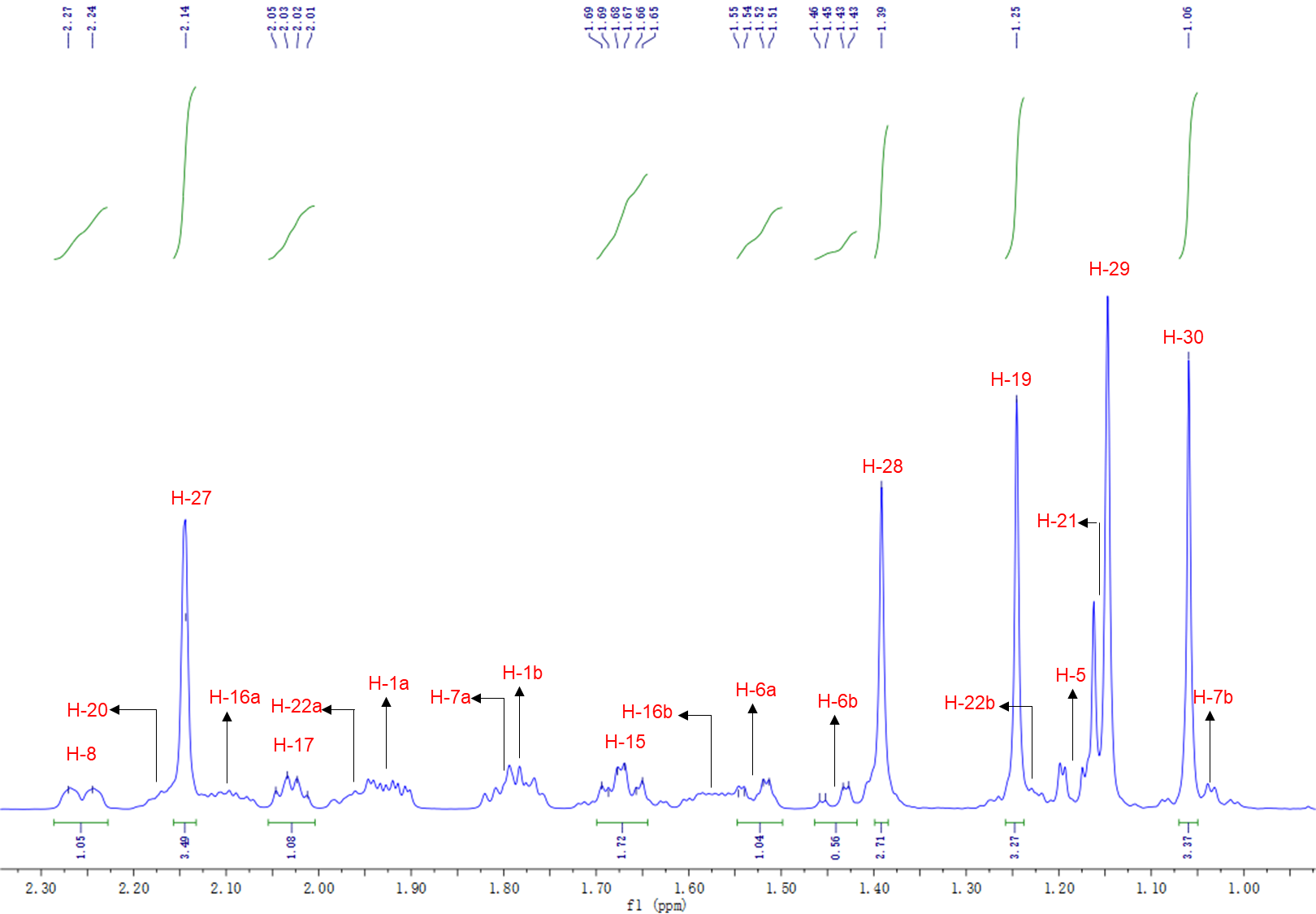


**Figure S5**. Enlarged ^1^H spectrum of Kadcoccitane A (**1**) in pyridine-*d*_5_.


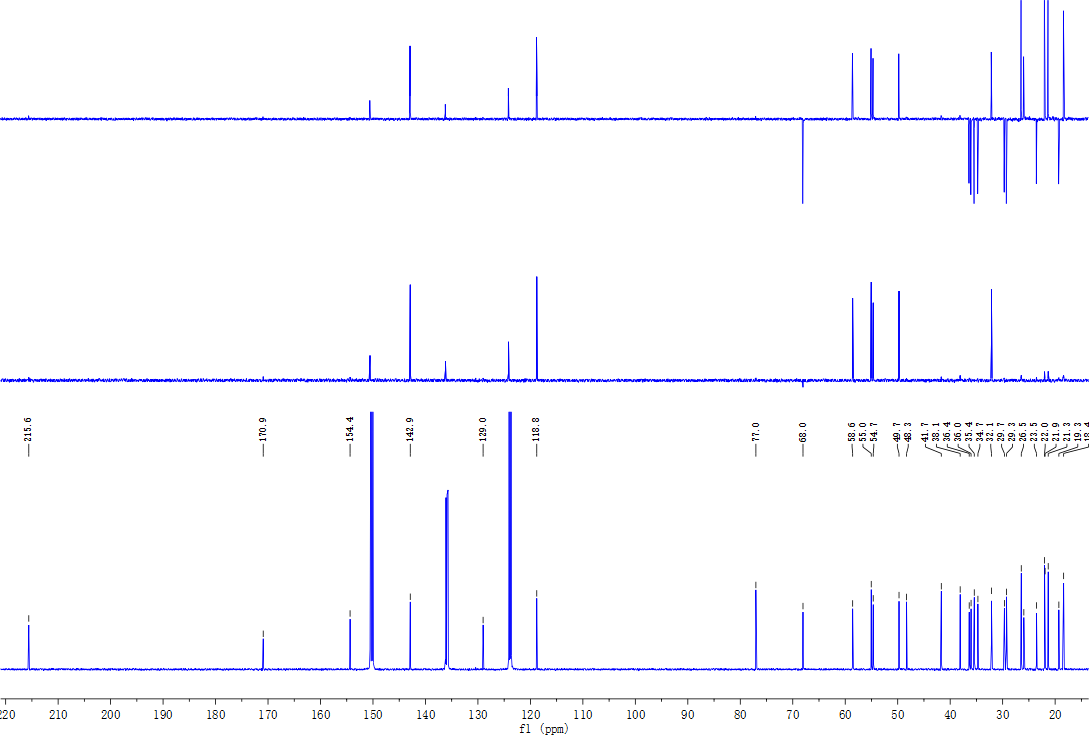


**Figure S6**. ^13^C and DEPT spectra of Kadcoccitane A (**1**) in pyridine-*d*_5_.


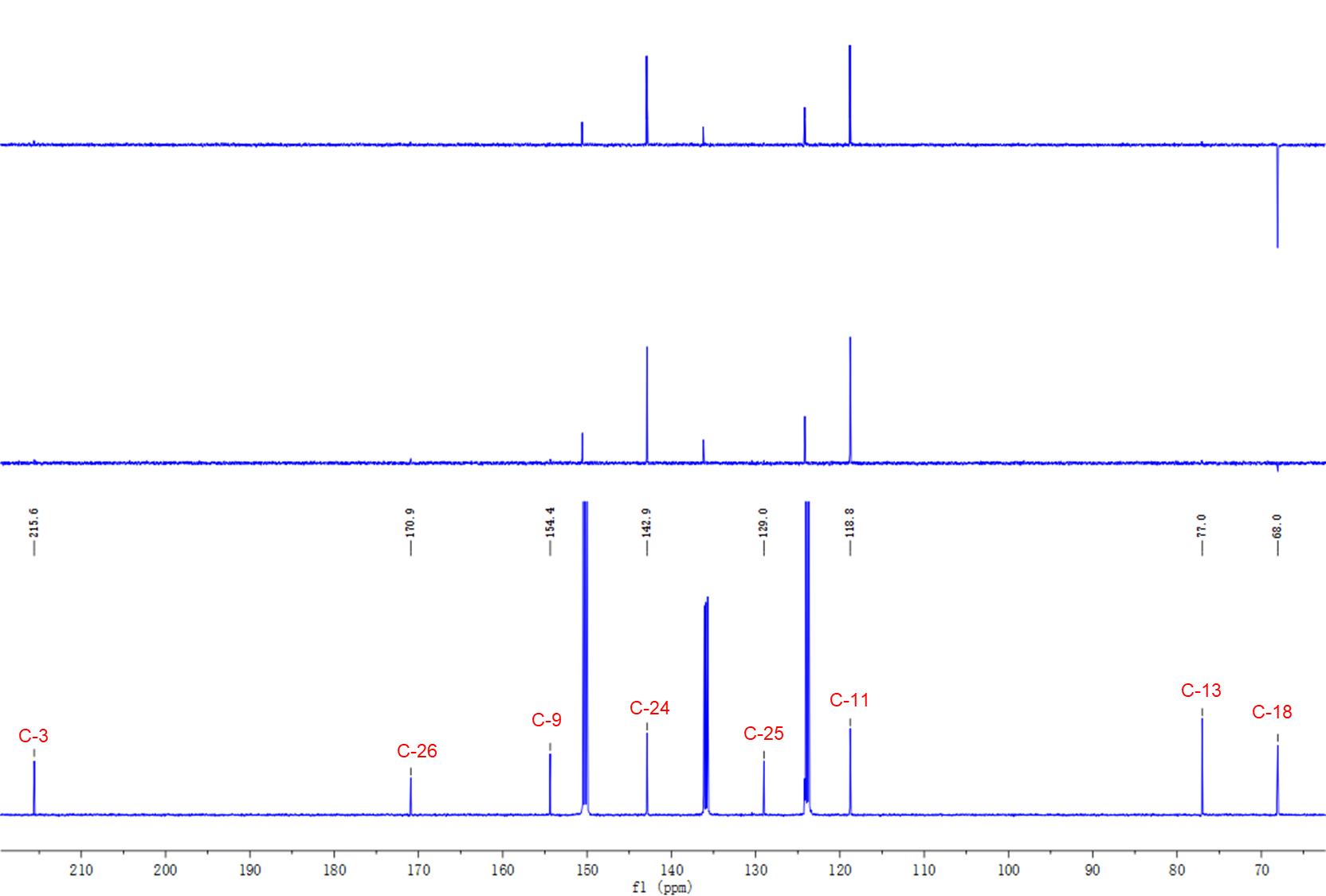


**Figure S7**. Enlarged ^13^C and DEPT spectra of Kadcoccitane A (**1**) in pyridine-*d*_5_.


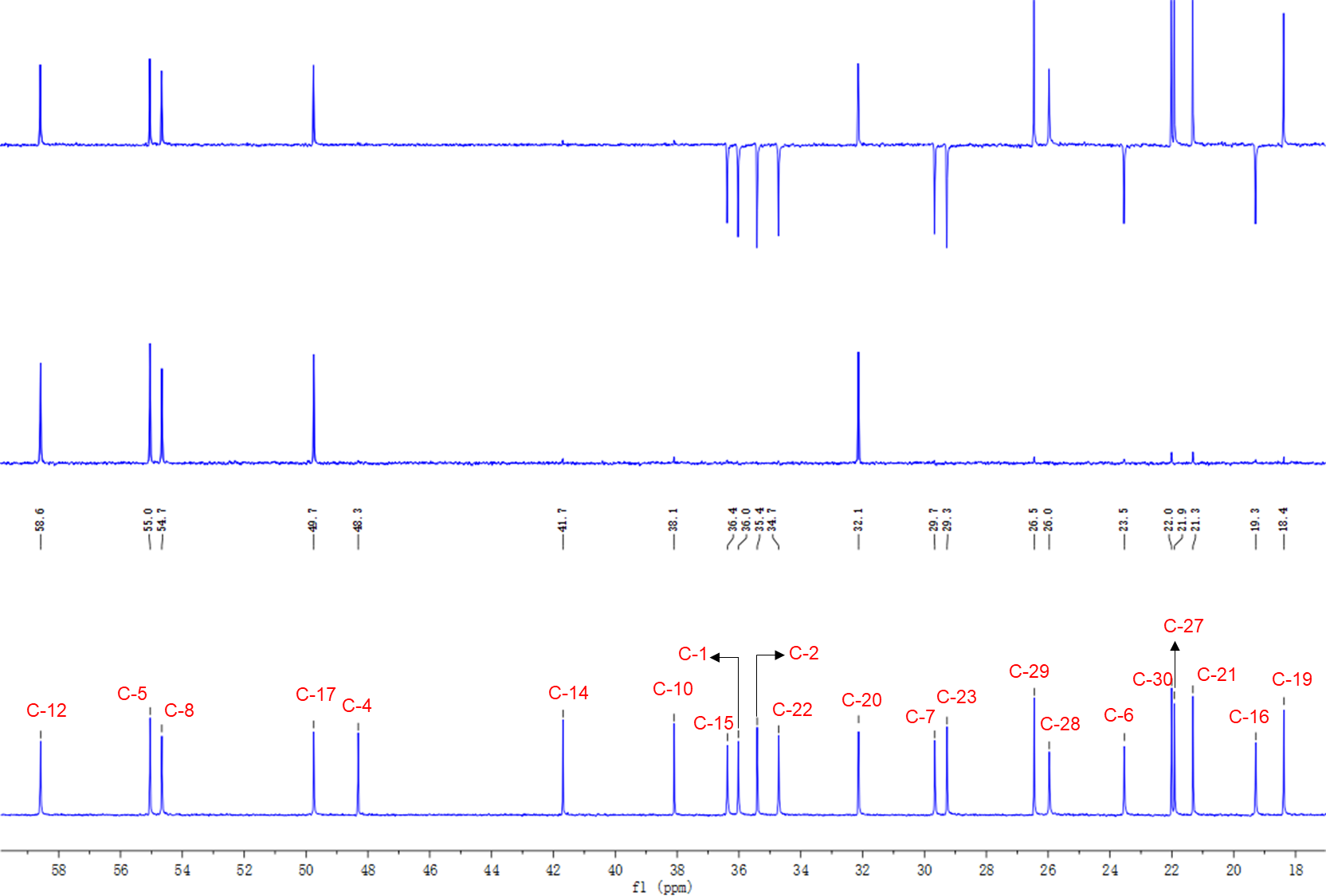


**Figure S8**. Enlarged ^13^C and DEPT spectra of Kadcoccitane A (**1**) in pyridine-*d*_5_.


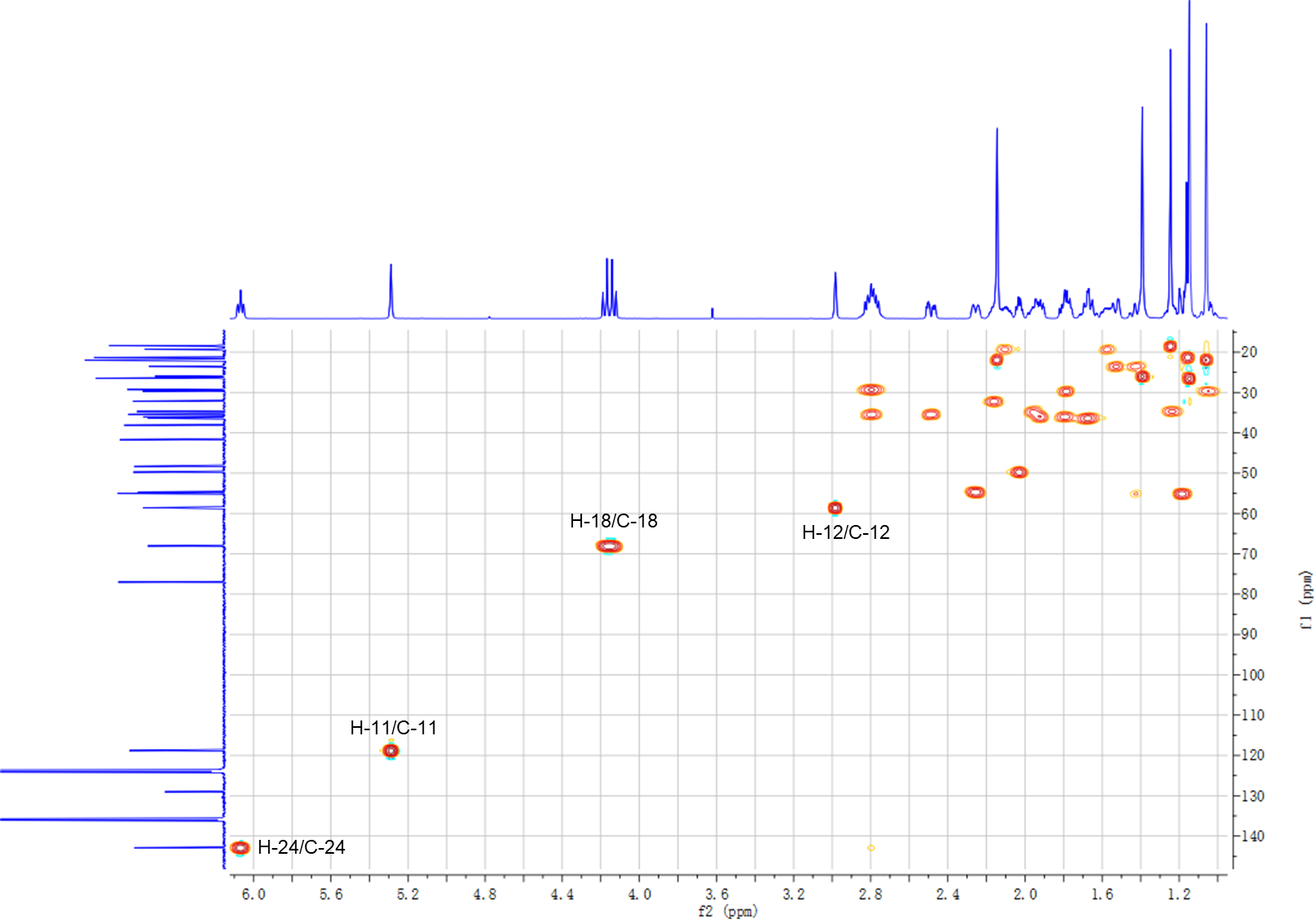


**Figure S9**. HSQC spectrum of Kadcoccitane A (**1**) in pyridine-*d*_5_.


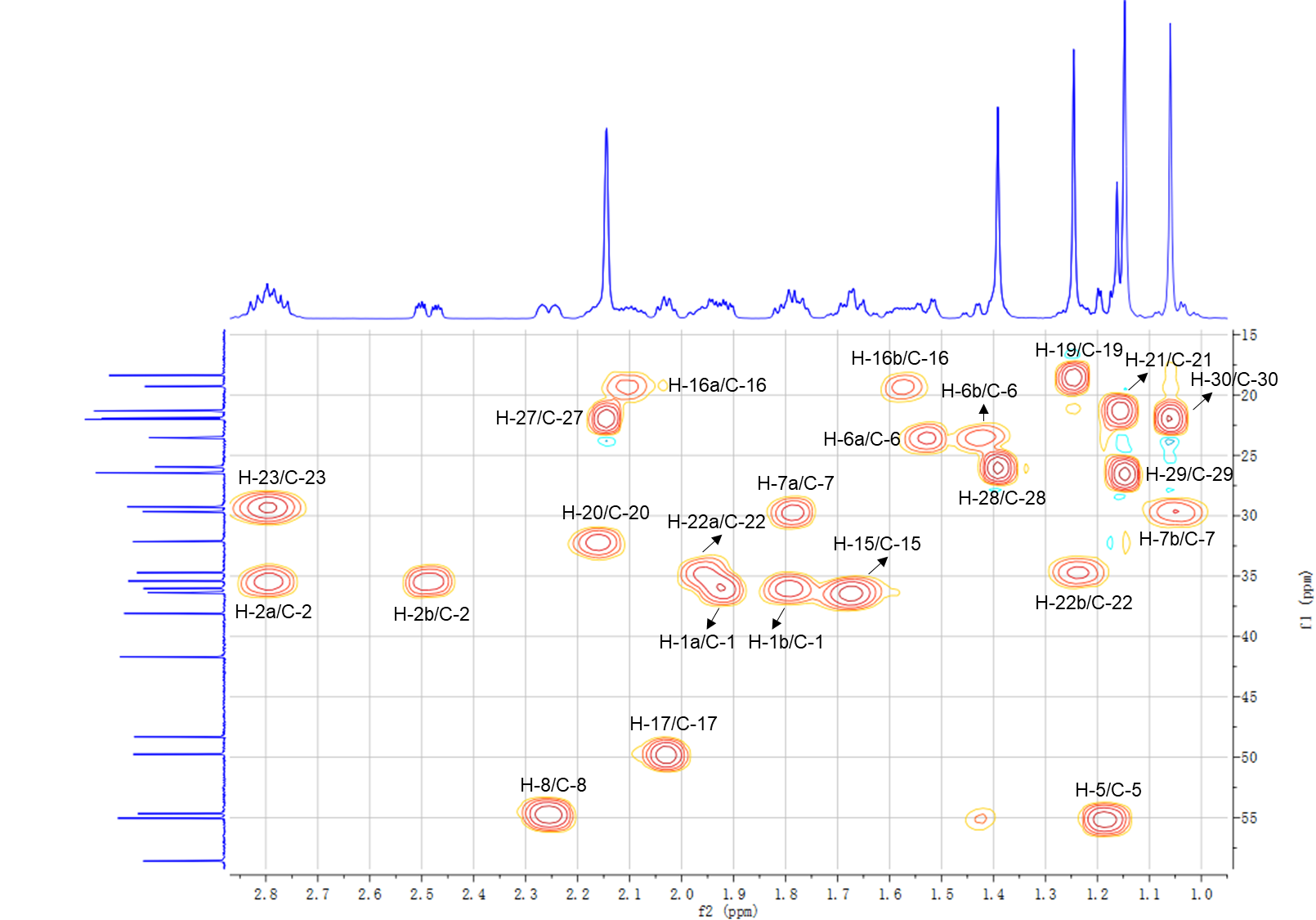


**Figure S10**. Enlarged HSQC spectrum of Kadcoccitane A (**1**) in pyridine-*d*_5_.


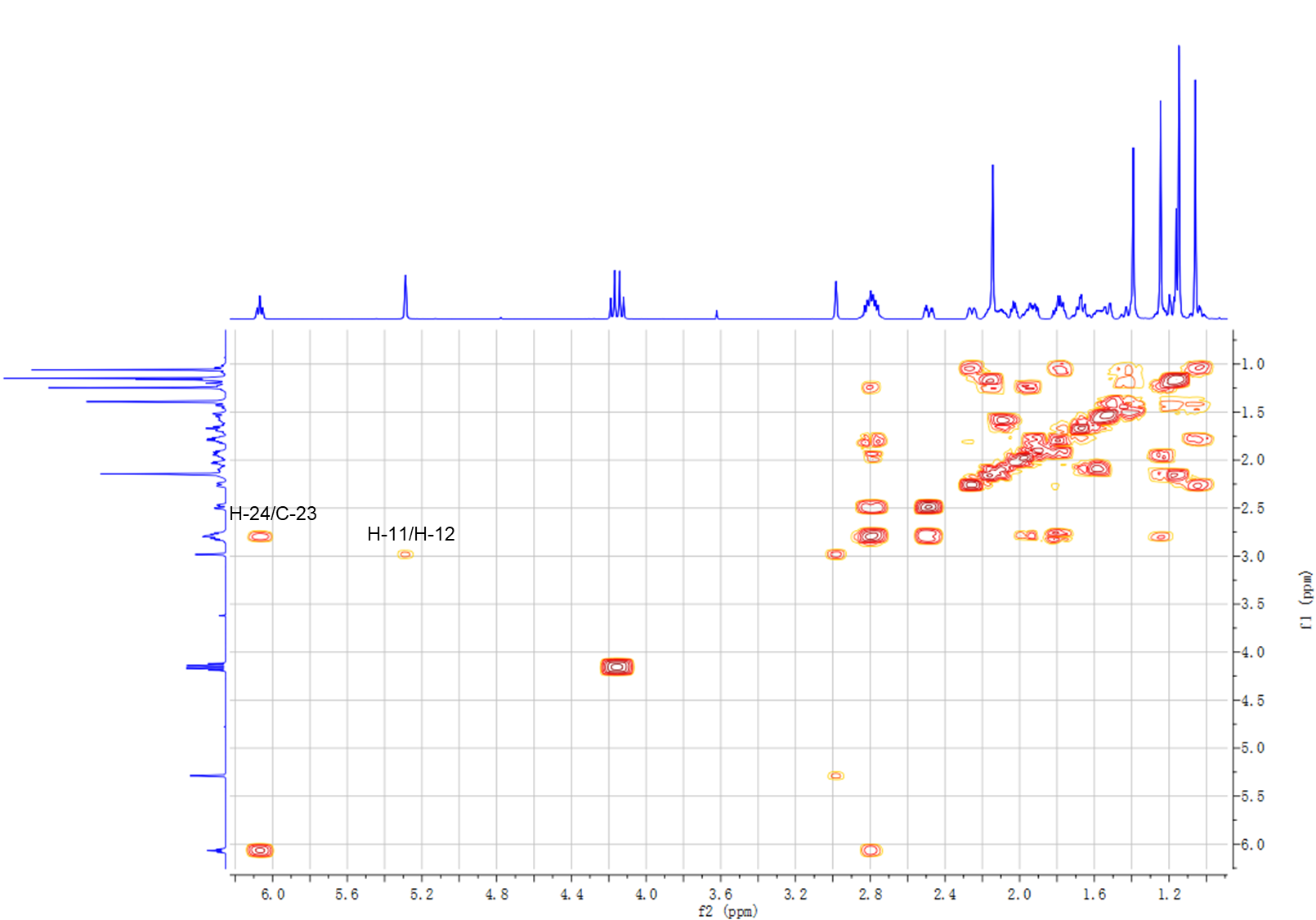


**Figure S11**. ^1^H-^1^H COSY spectrum of Kadcoccitane A (**1**) in pyridine-*d*_5_.


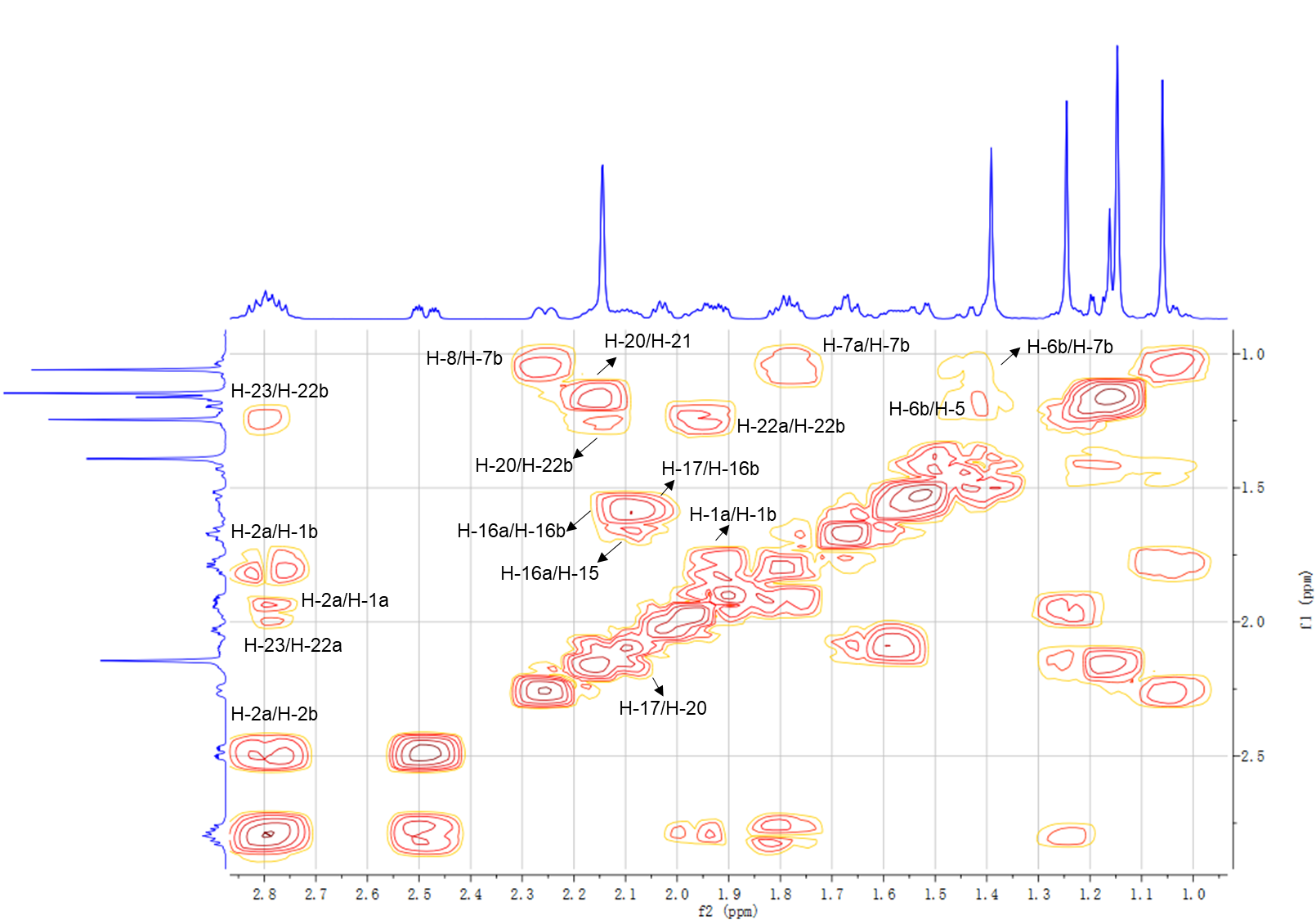


**Figure S12**. Enlarged ^1^H-^1^H COSY spectrum of Kadcoccitane A (**1**) in pyridine-*d*_5_.


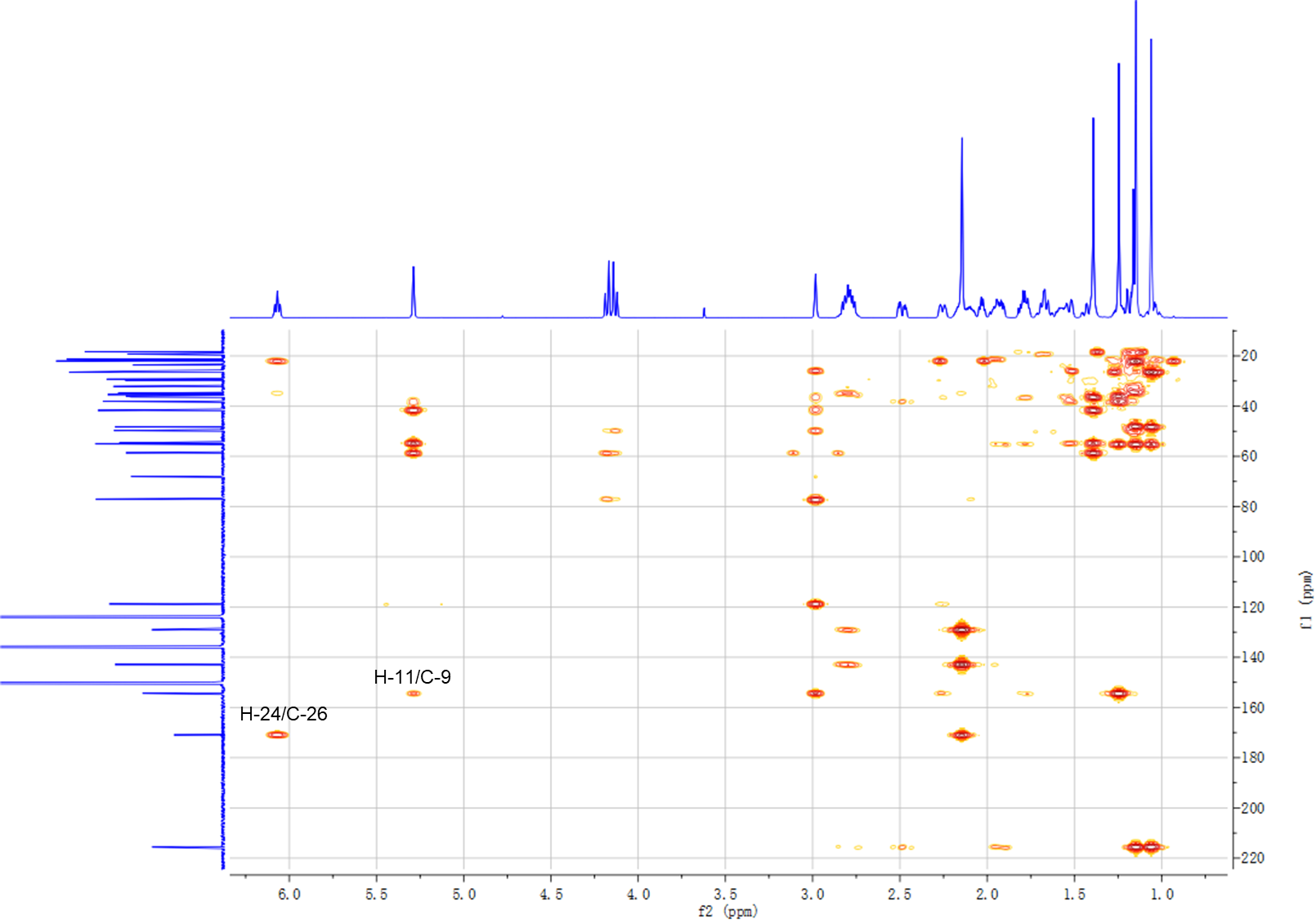


**Figure S13.** HMBC spectrum of Kadcoccitane A (**1**) in pyridine-*d*_5_.


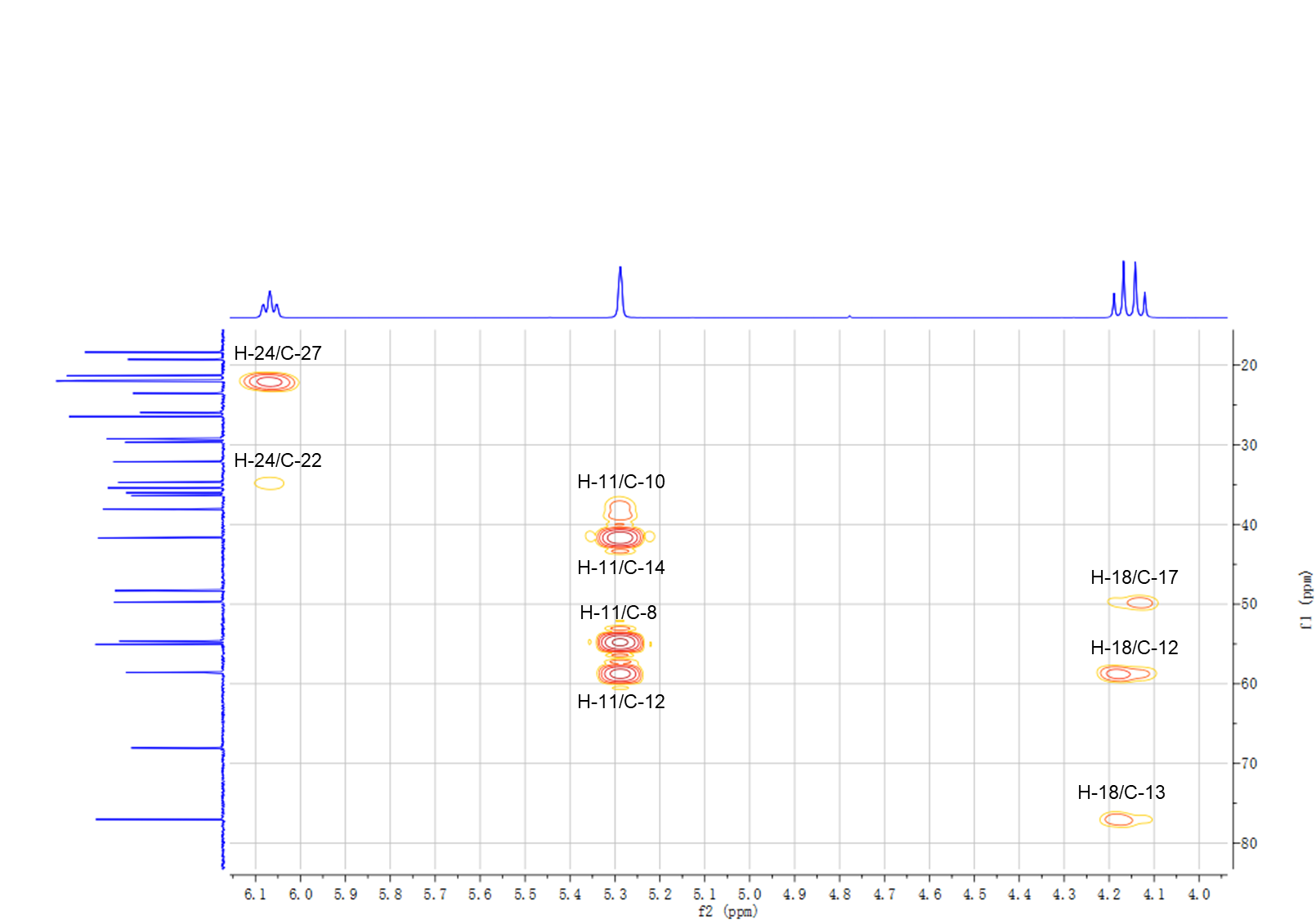


**Figure S14**. Enlarged HMBC spectrum of Kadcoccitane A (**1**) in pyridine-*d*_5_.


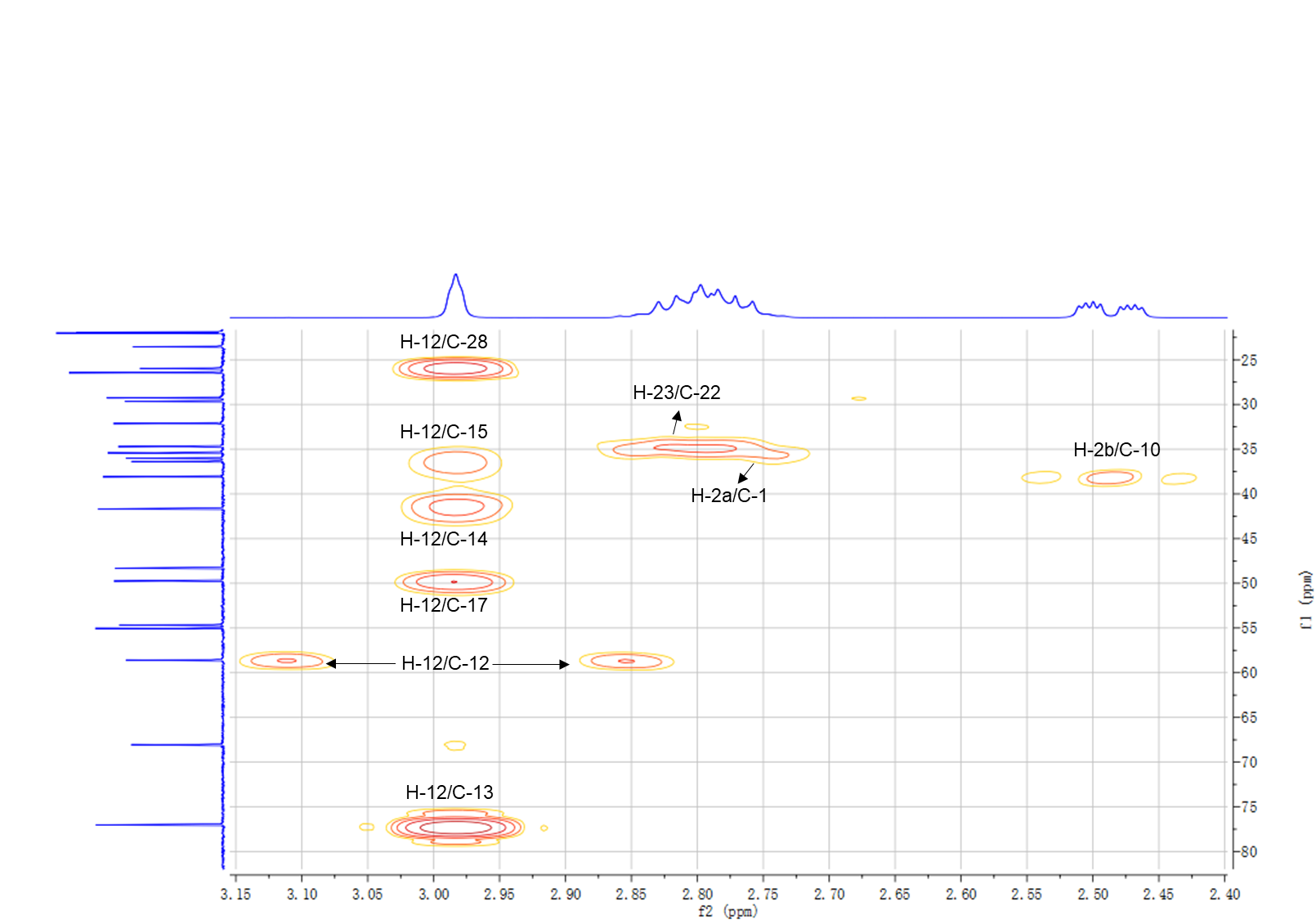


**Figure S15.** Enlarged HMBC spectrum of Kadcoccitane A (**1**) in pyridine-*d*_5_.


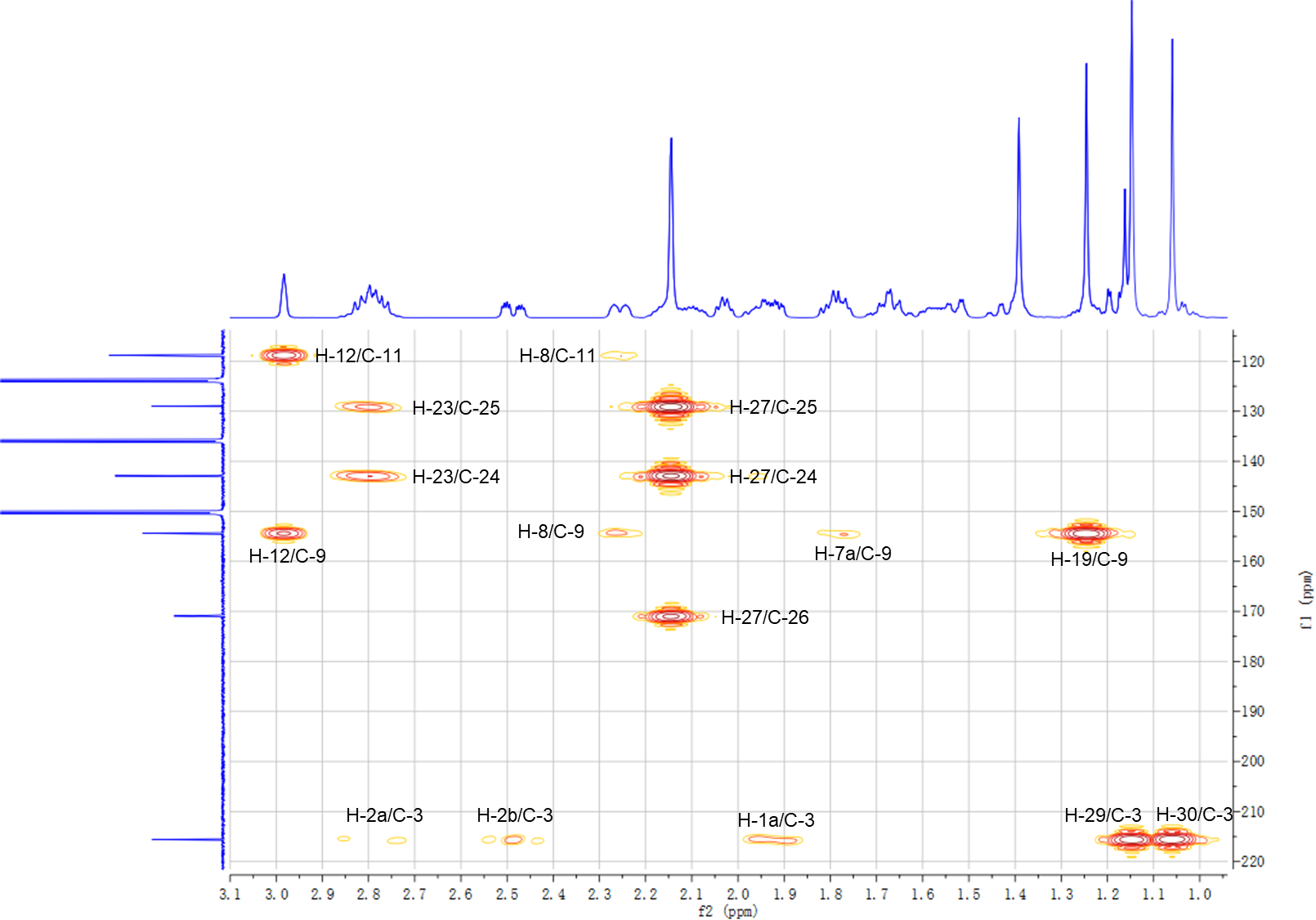


**Figure S16.** Enlarged HMBC spectrum of Kadcoccitane A (**1**) in pyridine-*d*_5_.


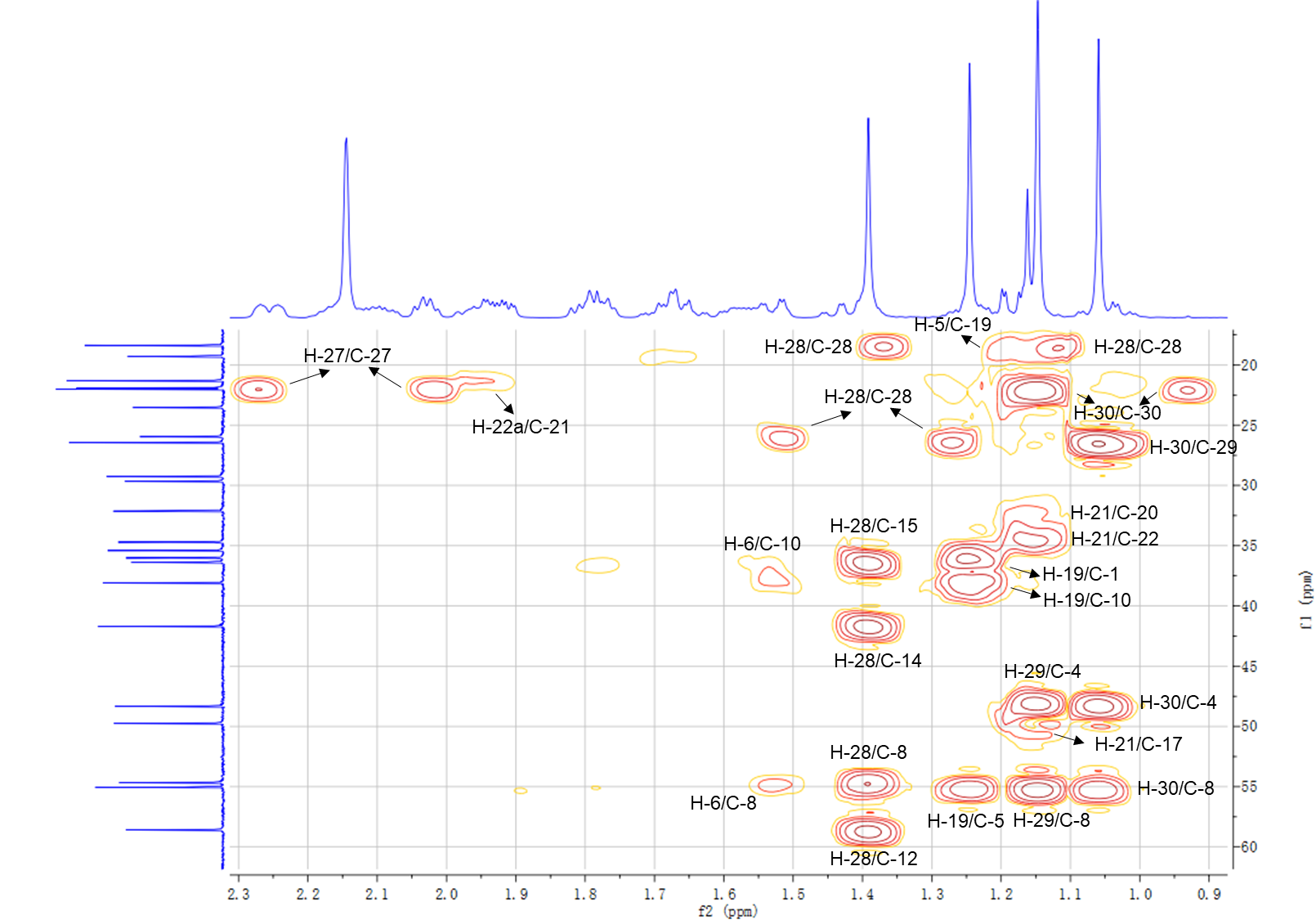


**Figure S17**. Enlarged HMBC spectrum of Kadcoccitane A (**1**) in pyridine-*d*_5_.


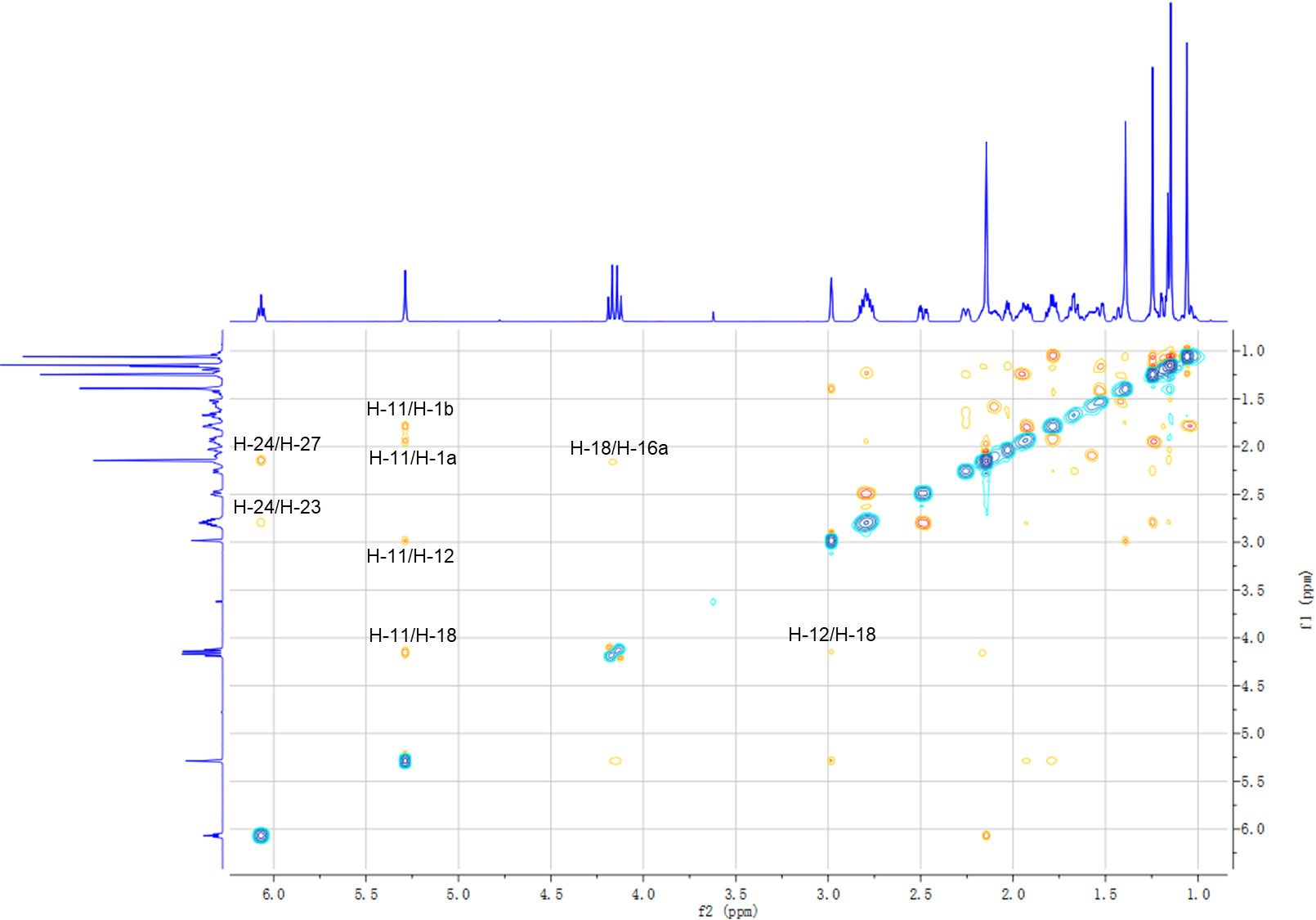


**Figure S18**. ROESY spectrum of Kadcoccitane A (**1**) in pyridine-*d*_5_.


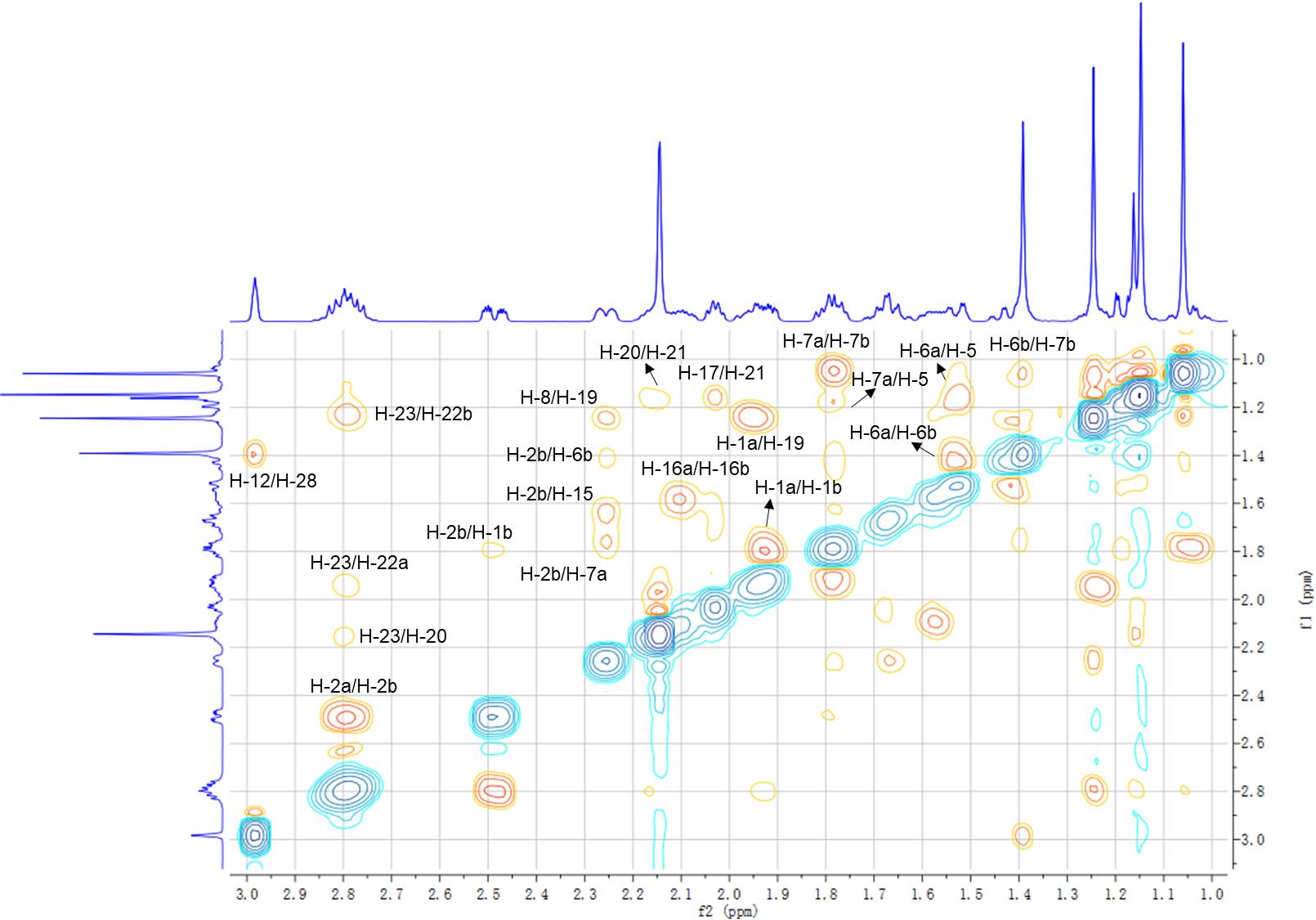


**Figure S19**. Enlarged ROESY spectrum of Kadcoccitane A (**1**) in pyridine-*d*_5_.


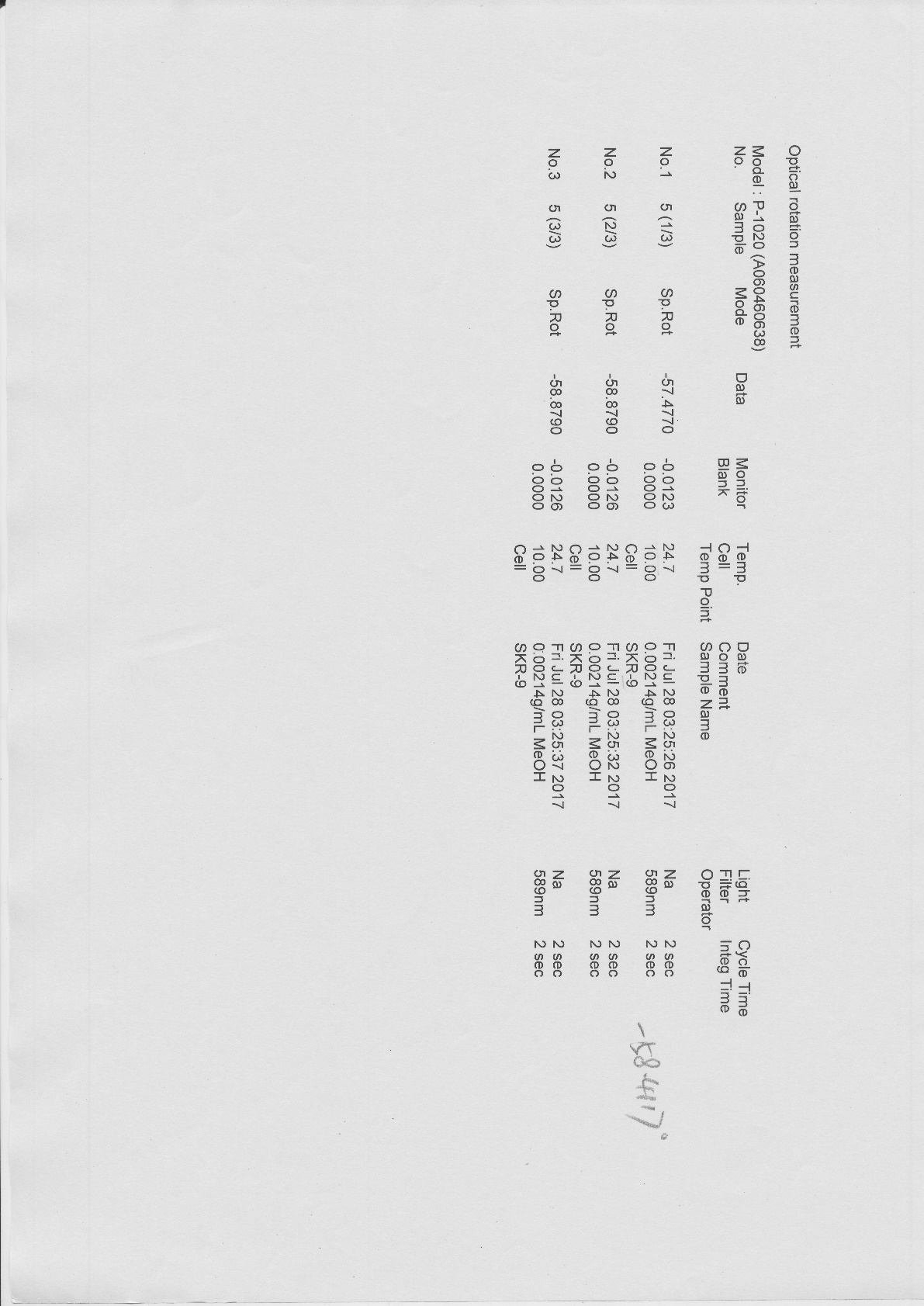


**Figure S20**. Optical rotation spectrum of Kadcoccitane A (**1**).


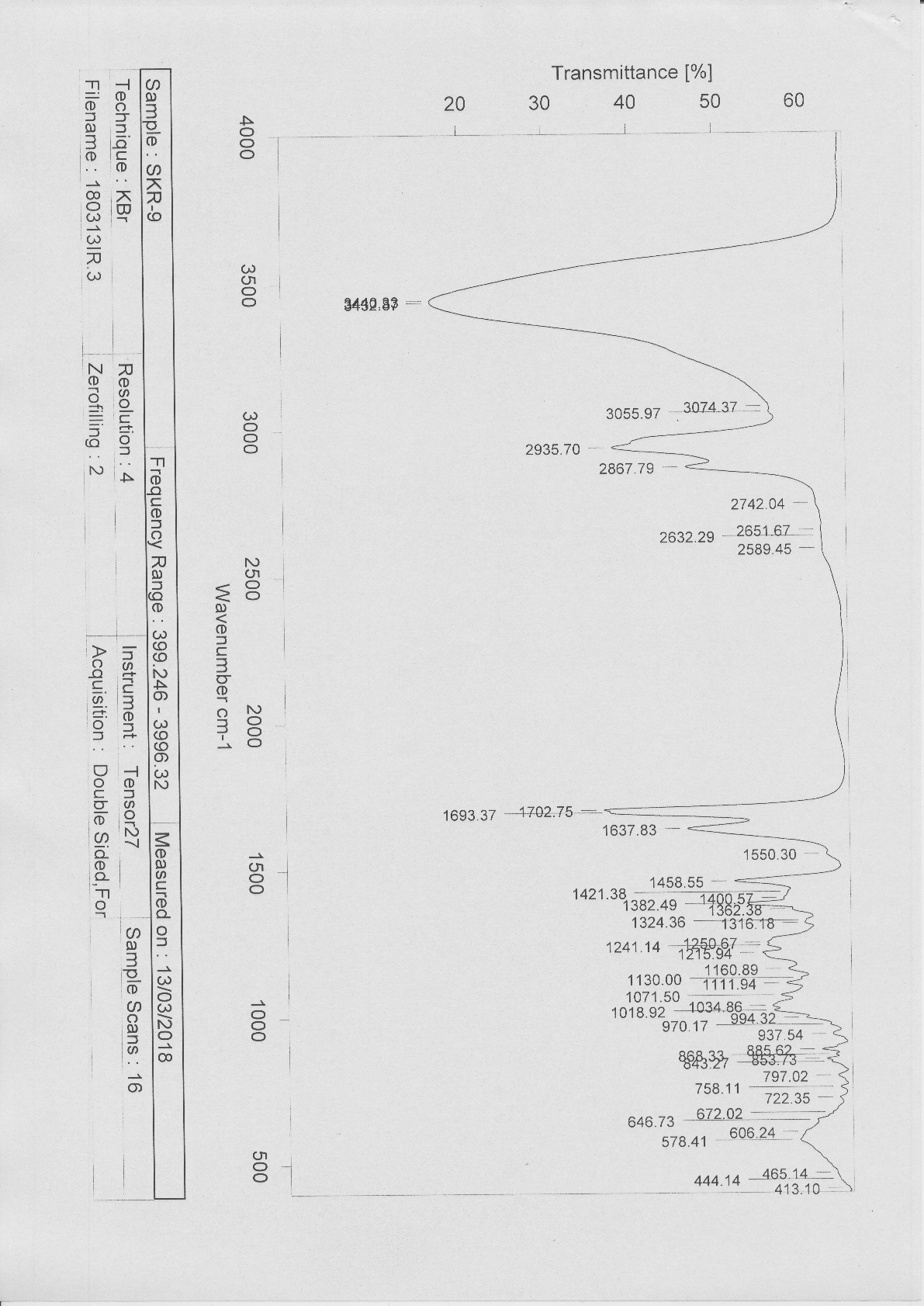


**Figure S21**. IR spectrum of Kadcoccitane A (1).


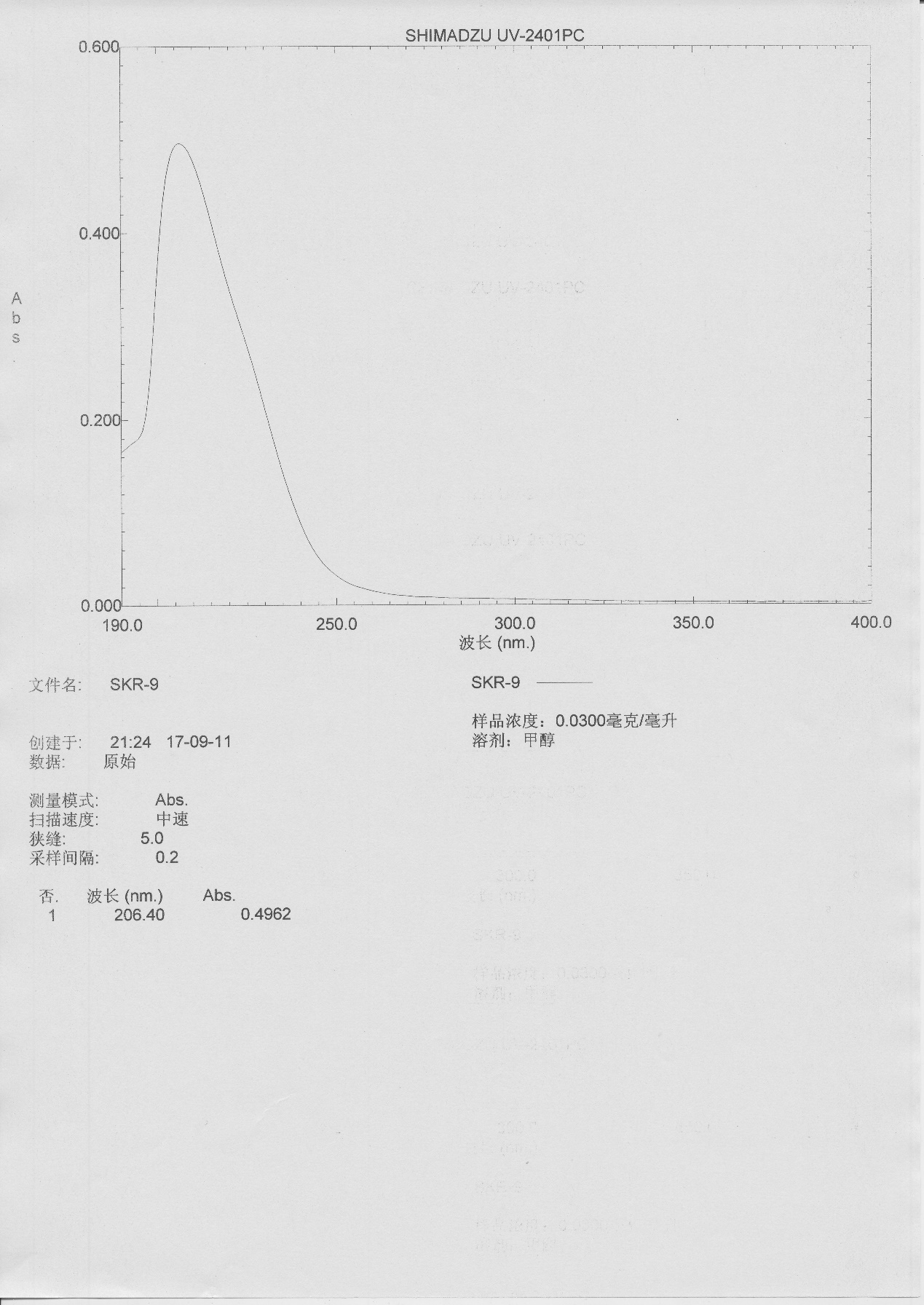


**Figure S22**. UV spectrum of Kadcoccitane A (1).


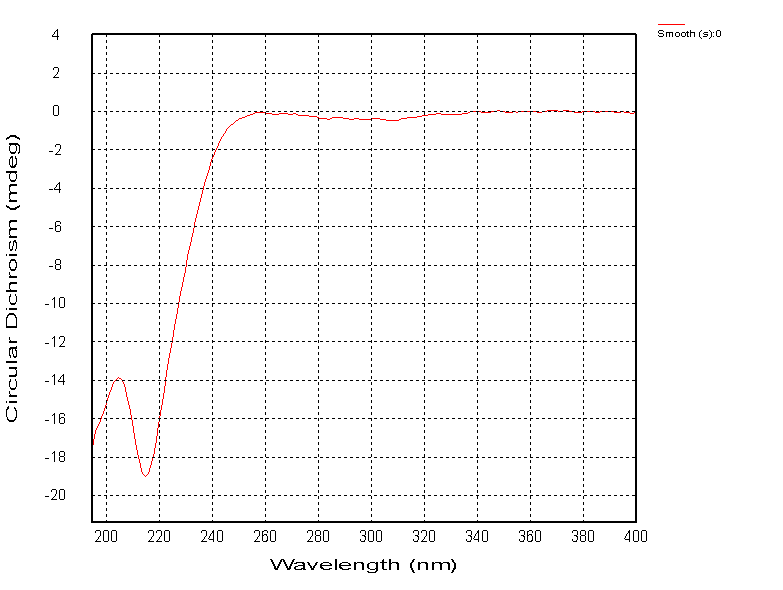


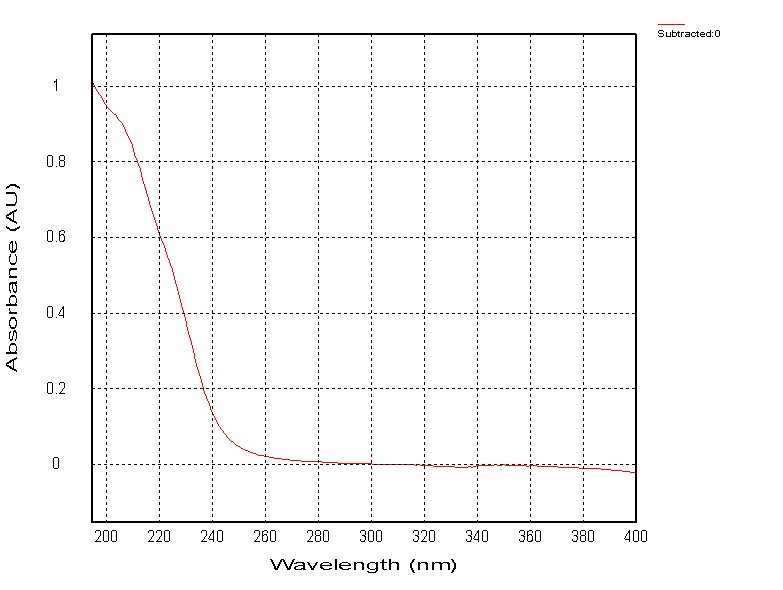


**Figure S23.** ECD (top) and UV (bottom) spectra of Kadcoccitane A (**1**)


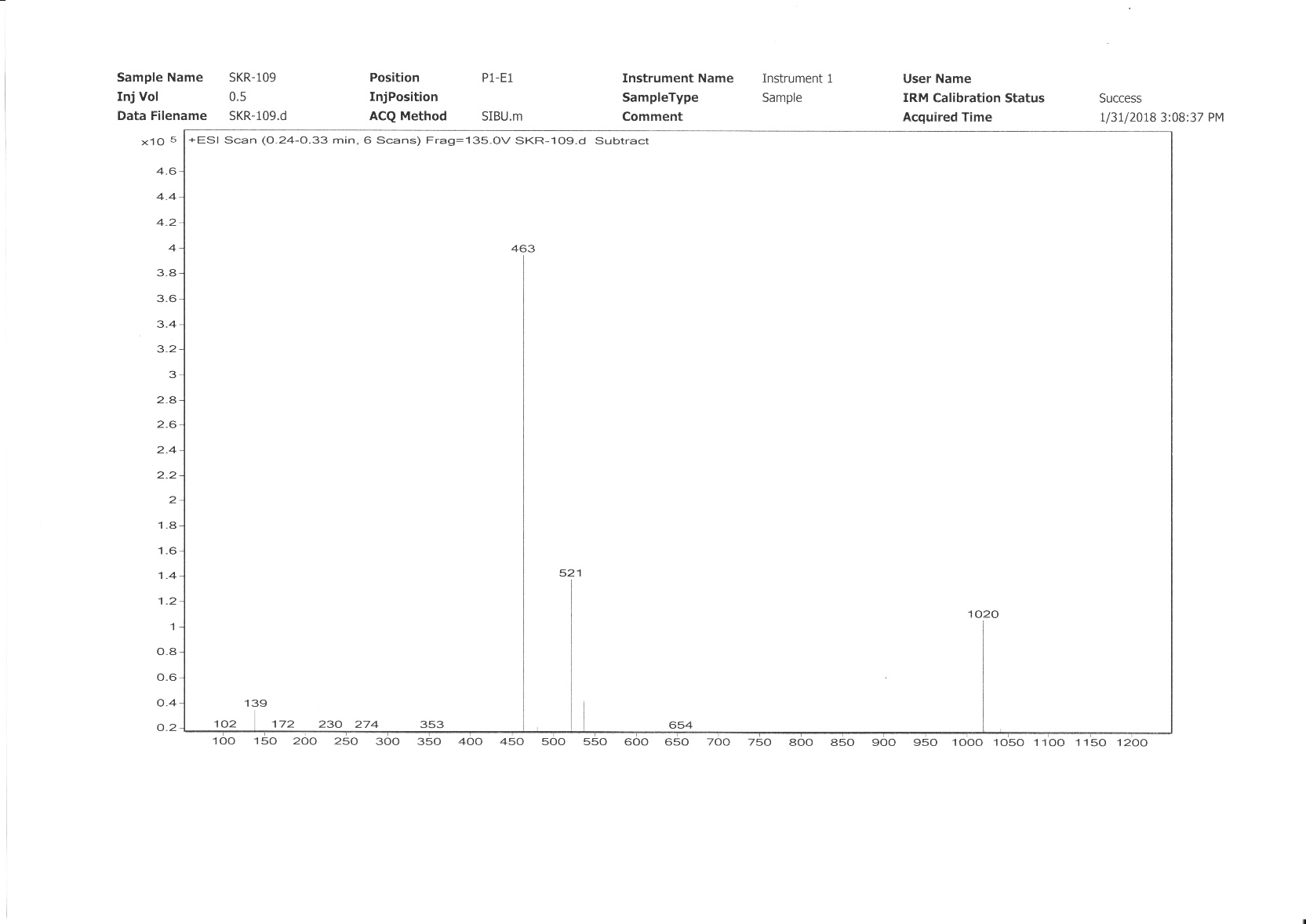


**Figure S24**. ESIMS spectrum of Kadcoccitane B (**2**).


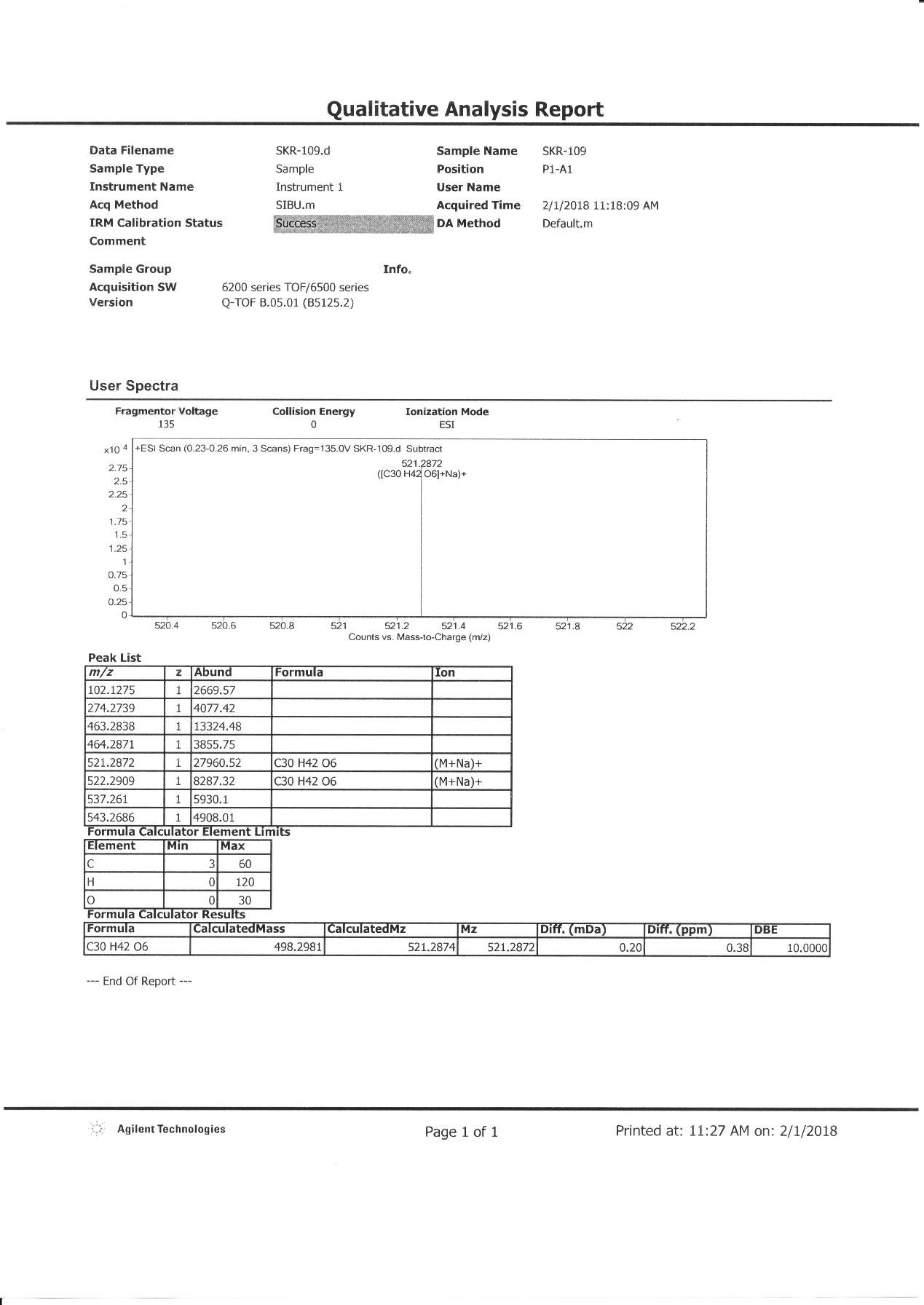


**Figure S25.** HRESIMS spectrum of Kadcoccitane B (**2**).


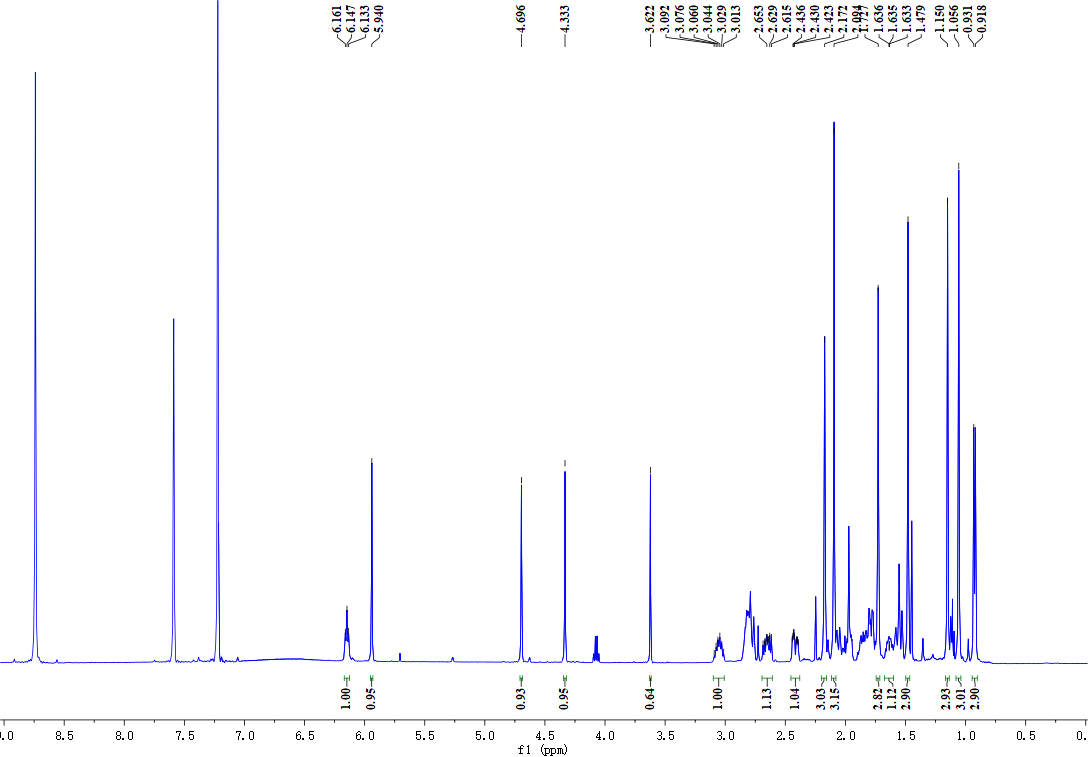


**Figure S26.** ^1^H spectrum of Kadcoccitane B (**2**) in pyridine-*d*_5_.


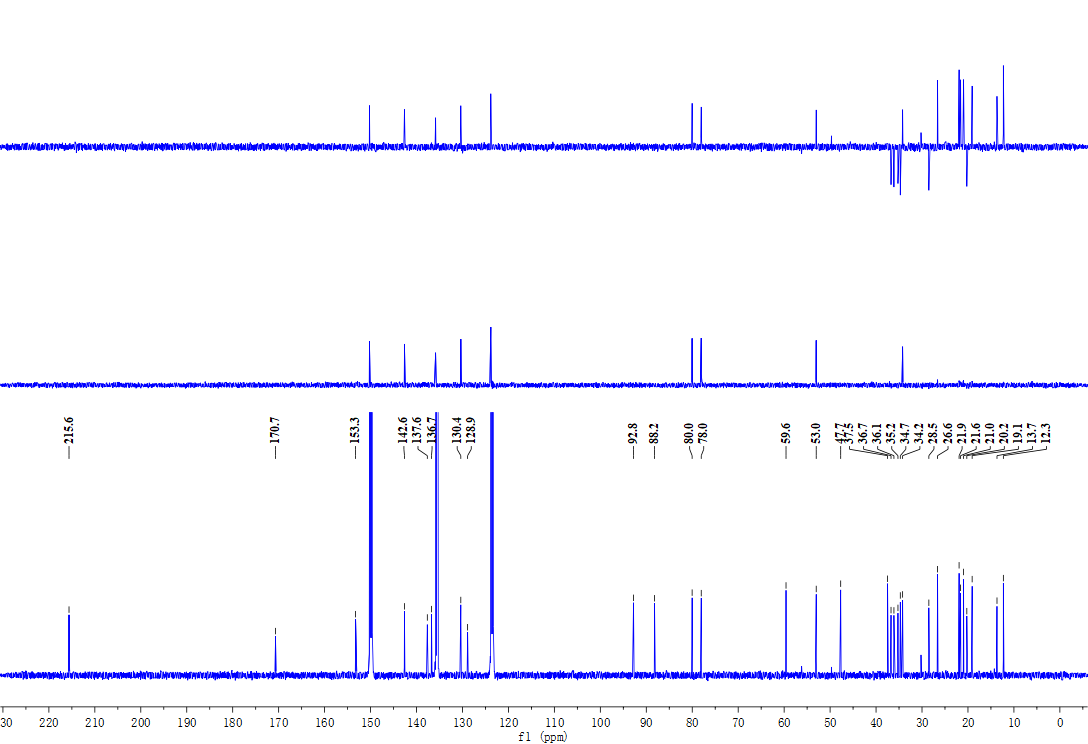


**Figure S27.** ^13^C and DEPT spectra of Kadcoccitane B (**2**) in pyridine-*d*_5_.


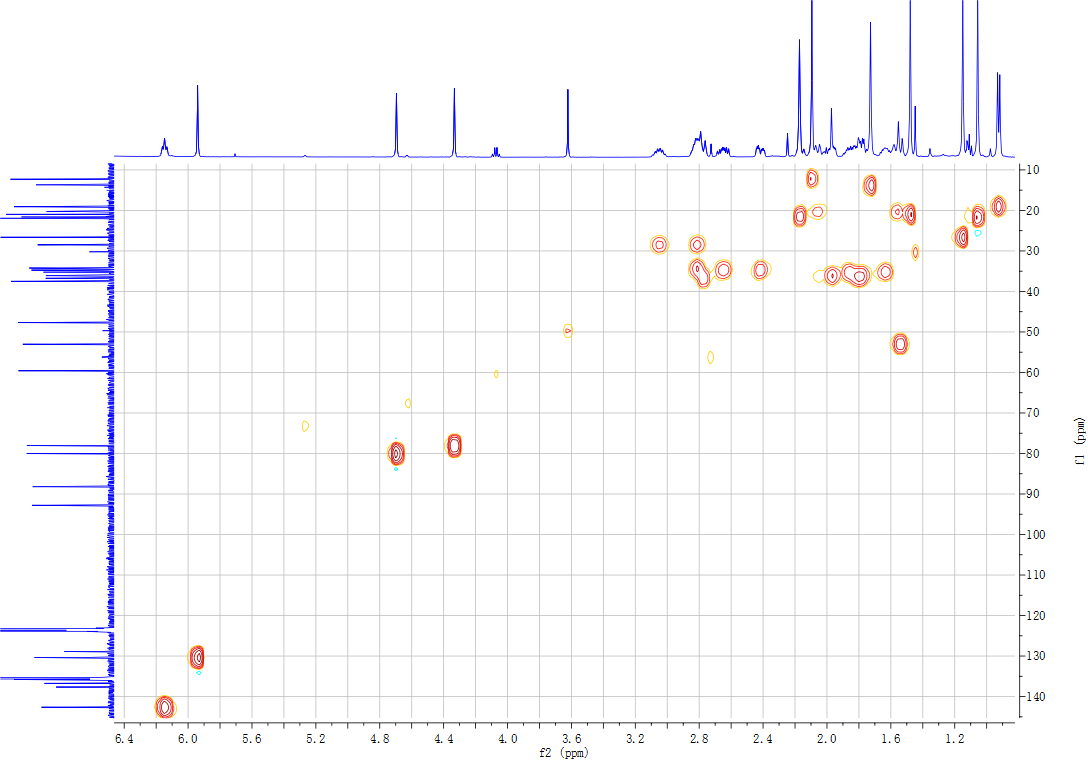


**Figure S28.** HSQC spectrum of Kadcoccitane B (**2**) in pyridine-*d*_5_.


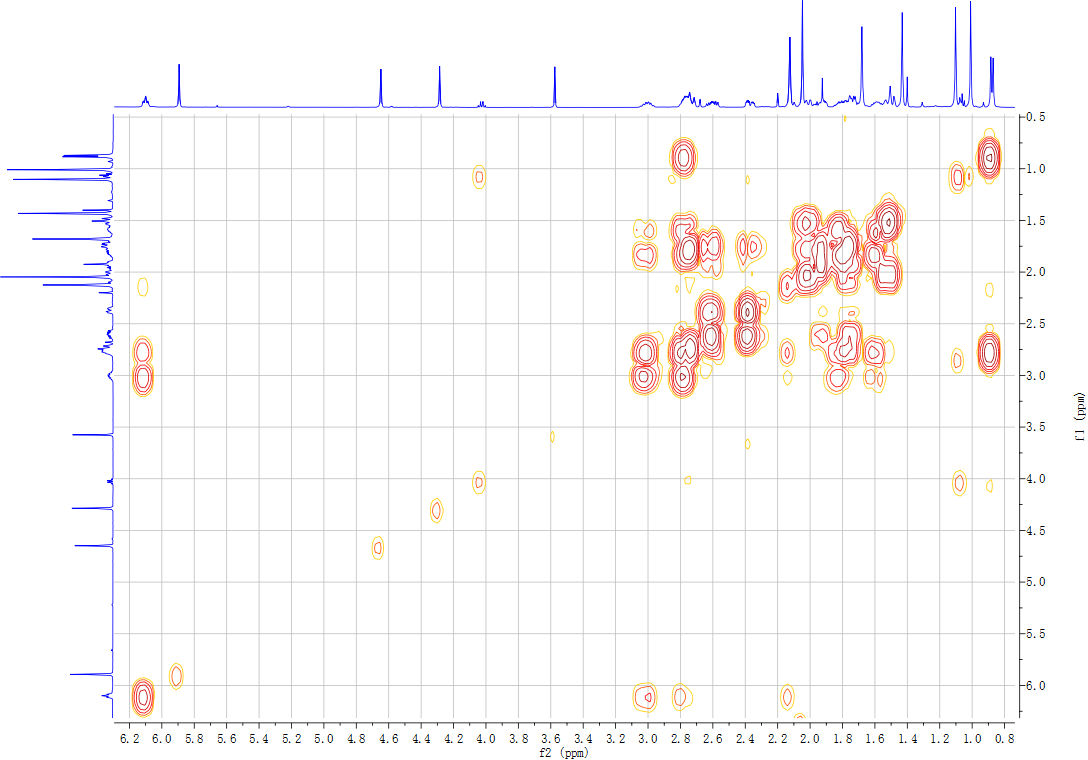


**Figure S29.** ^1^H-^1^H COSY spectrum of Kadcoccitane B (**2**) in pyridine-*d*_5_.


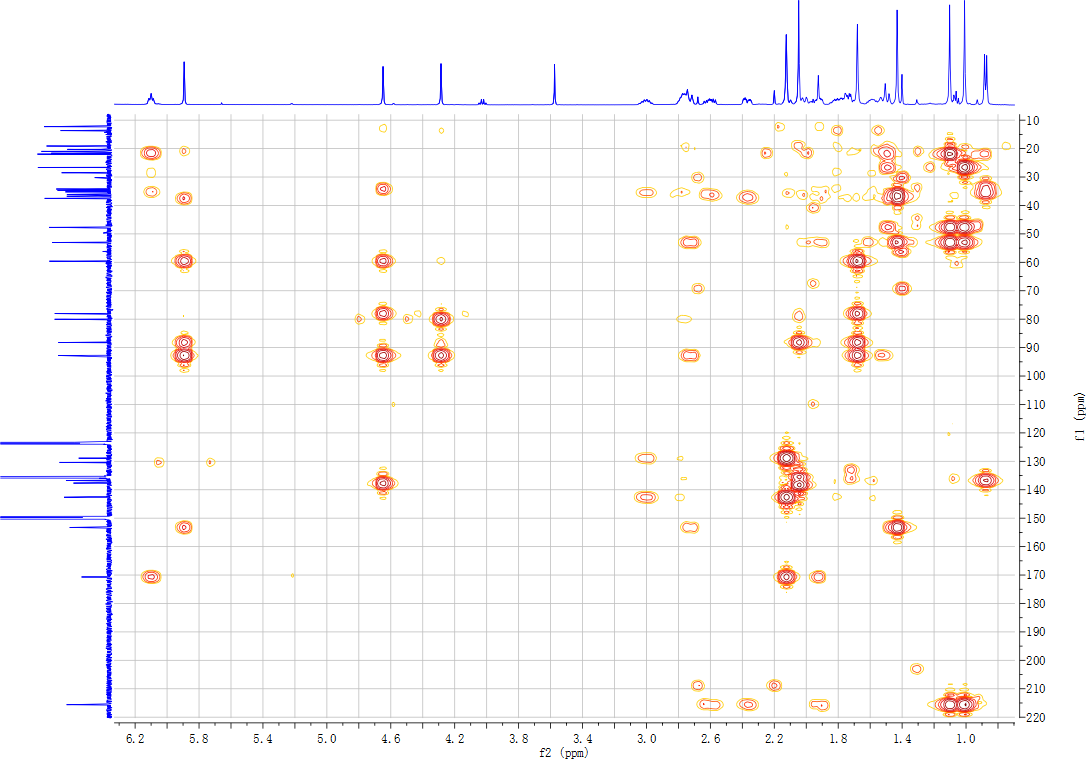


**Figure S30**. HMBC spectrum of Kadcoccitane B (**2**) in pyridine-*d*_5_.


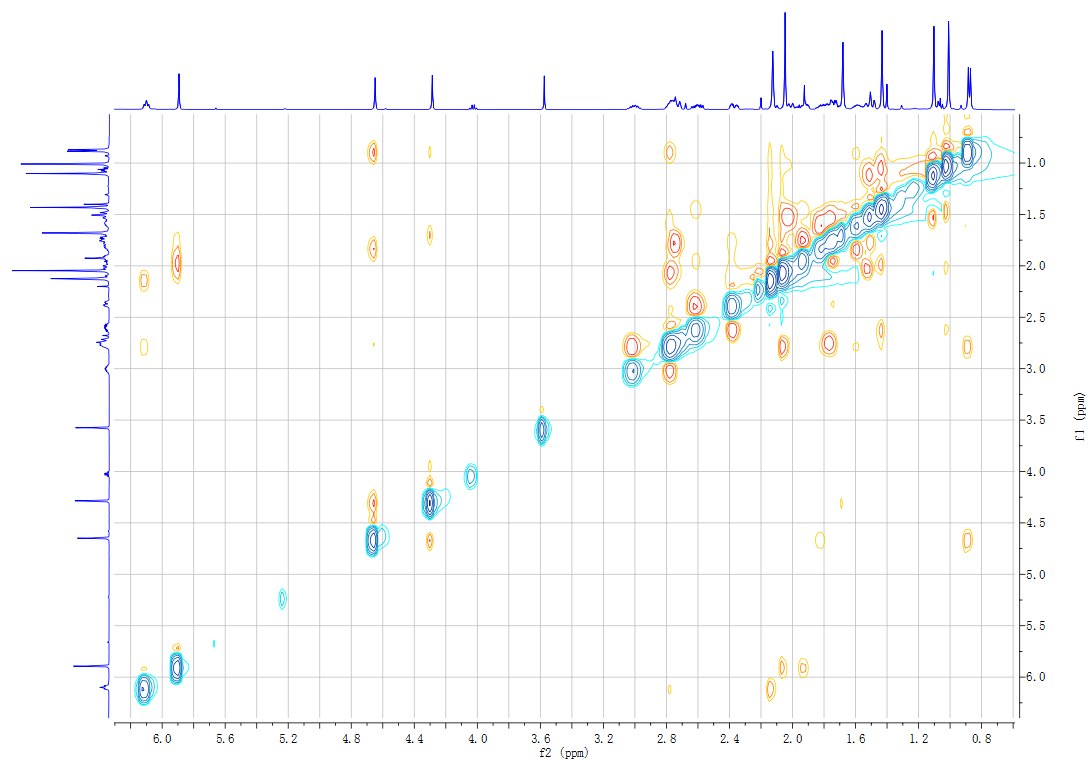


**Figure S31.** ROESY spectrum of Kadcoccitane B (**2**) in pyridine-*d*_5_.


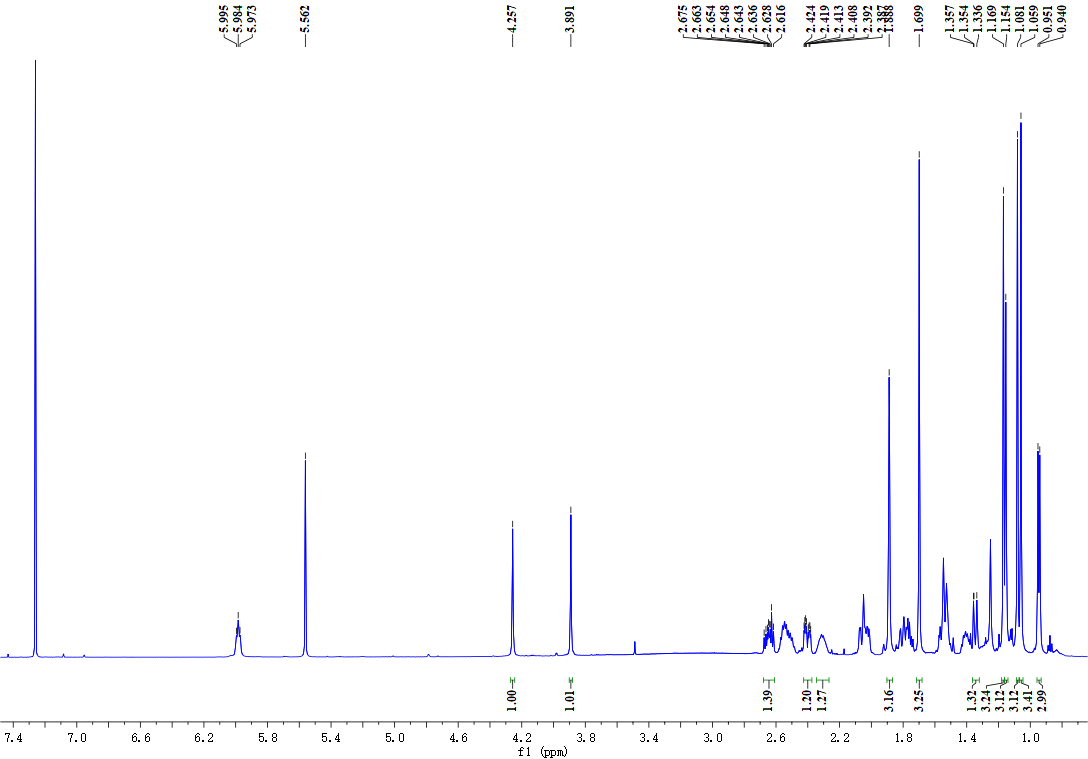


**Figure S32.** ^1^H spectrum of Kadcoccitane B (**2**) in CDCl_3_.


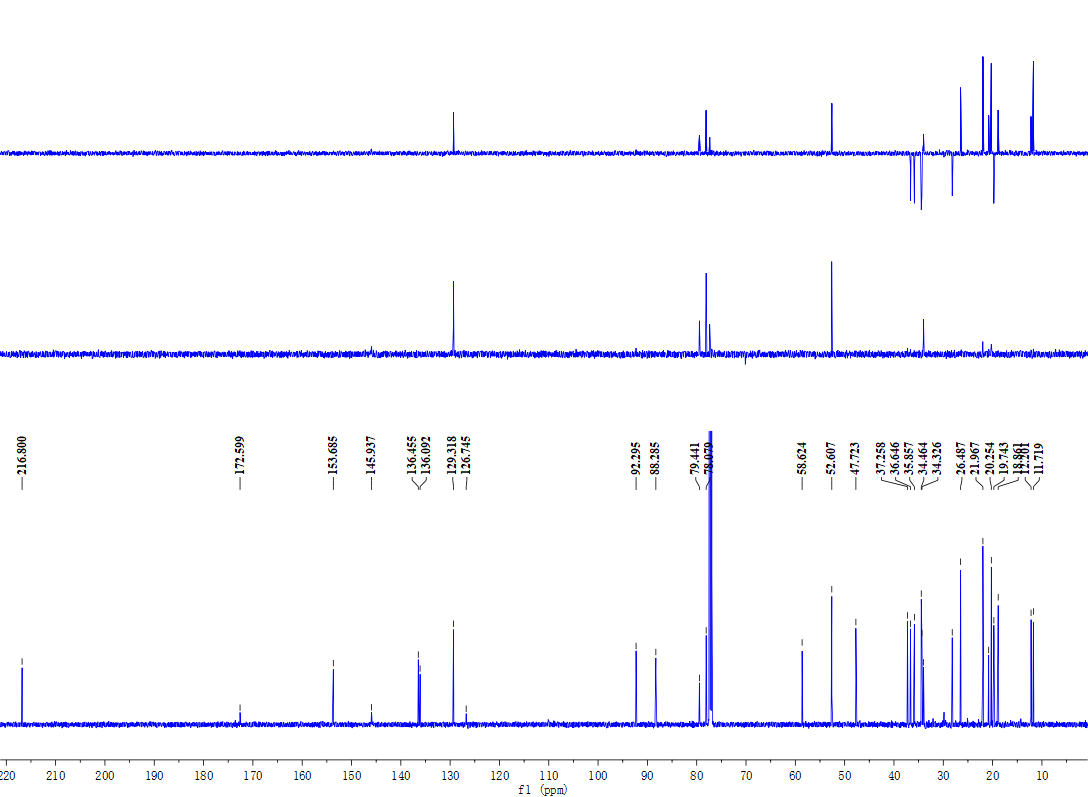


**Figure S33.** ^13^C and DEPT spectra of Kadcoccitane B (**2**) in CDCl_3_.


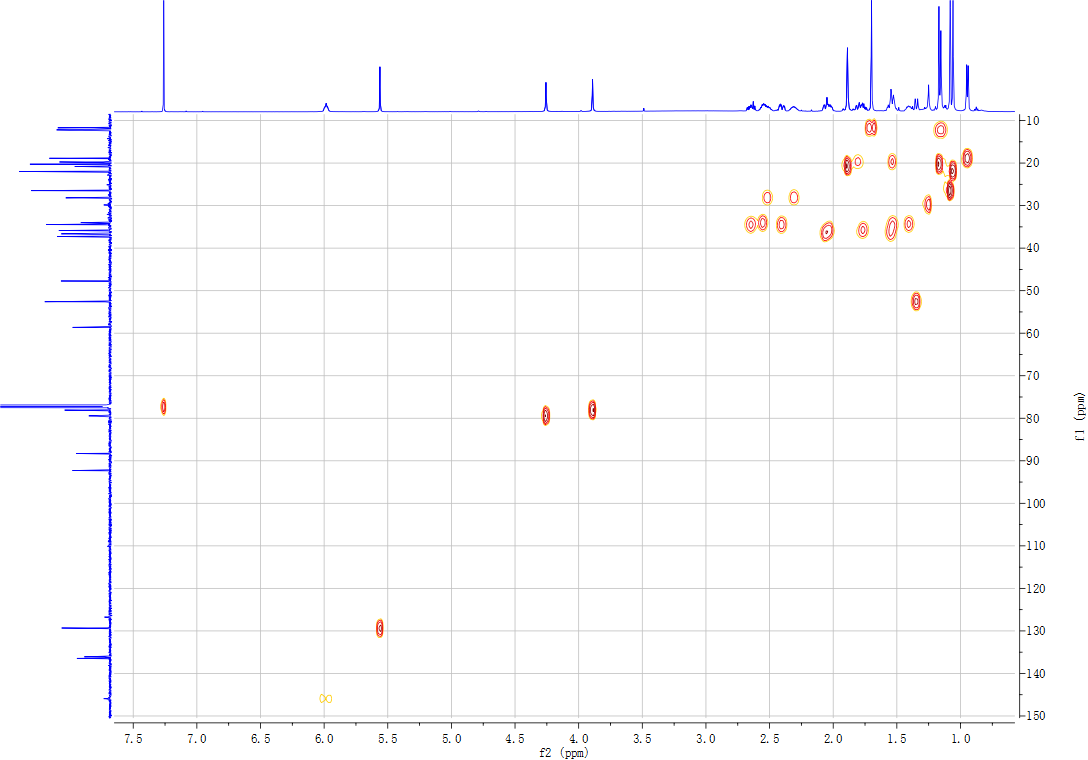


**Figure S34.** HSQC spectrum of Kadcoccitane B (**2**) in CDCl_3_.


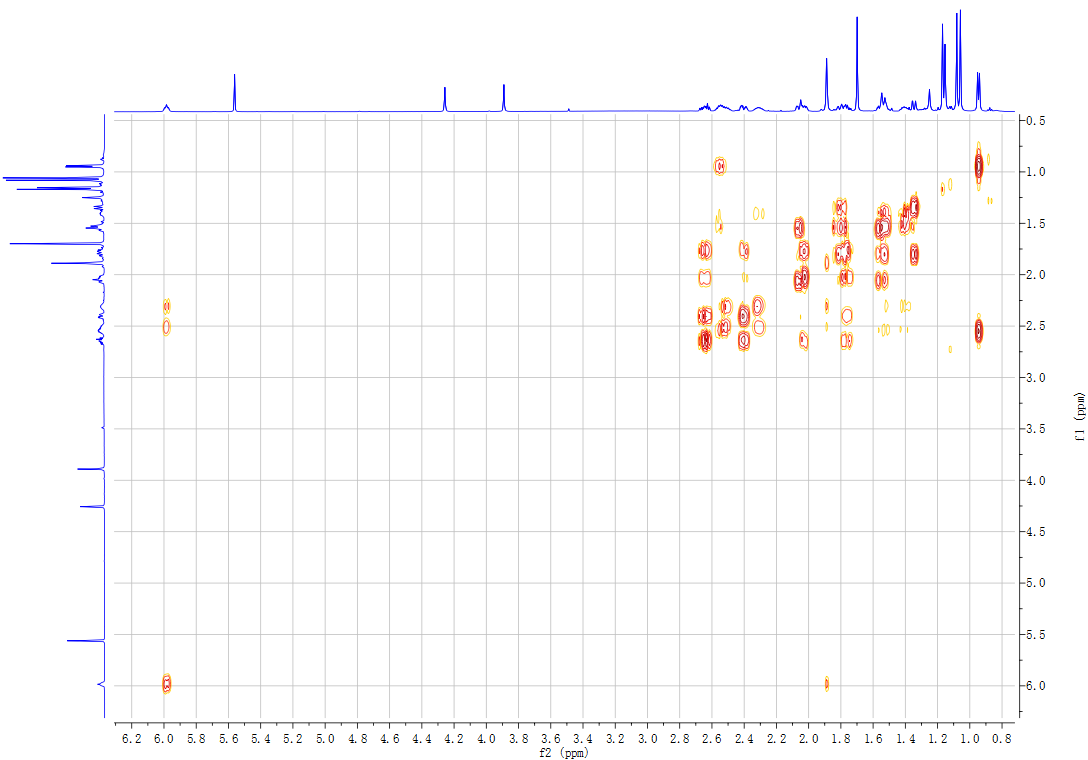


**Figure S35.** ^1^H-^1^H COSY spectrum of Kadcoccitane B (**2**) in CDCl_3_.


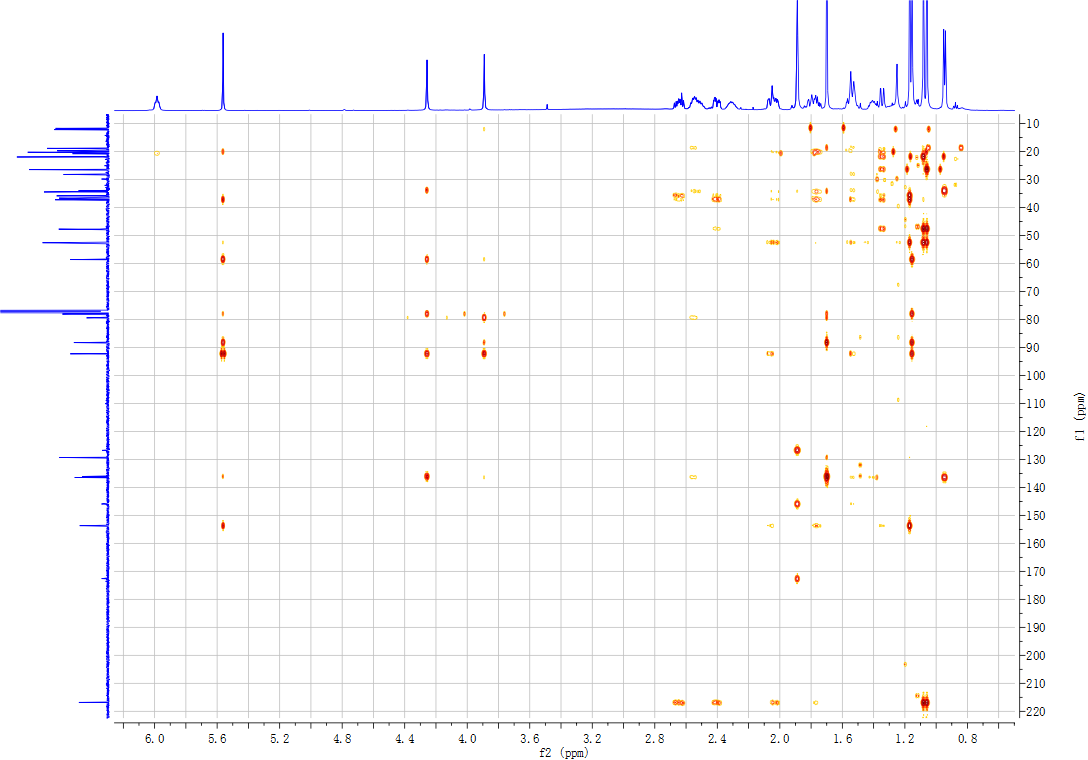


**Figure S36.** HMBC spectrum of Kadcoccitane B (**2**) in CDCl_3_.


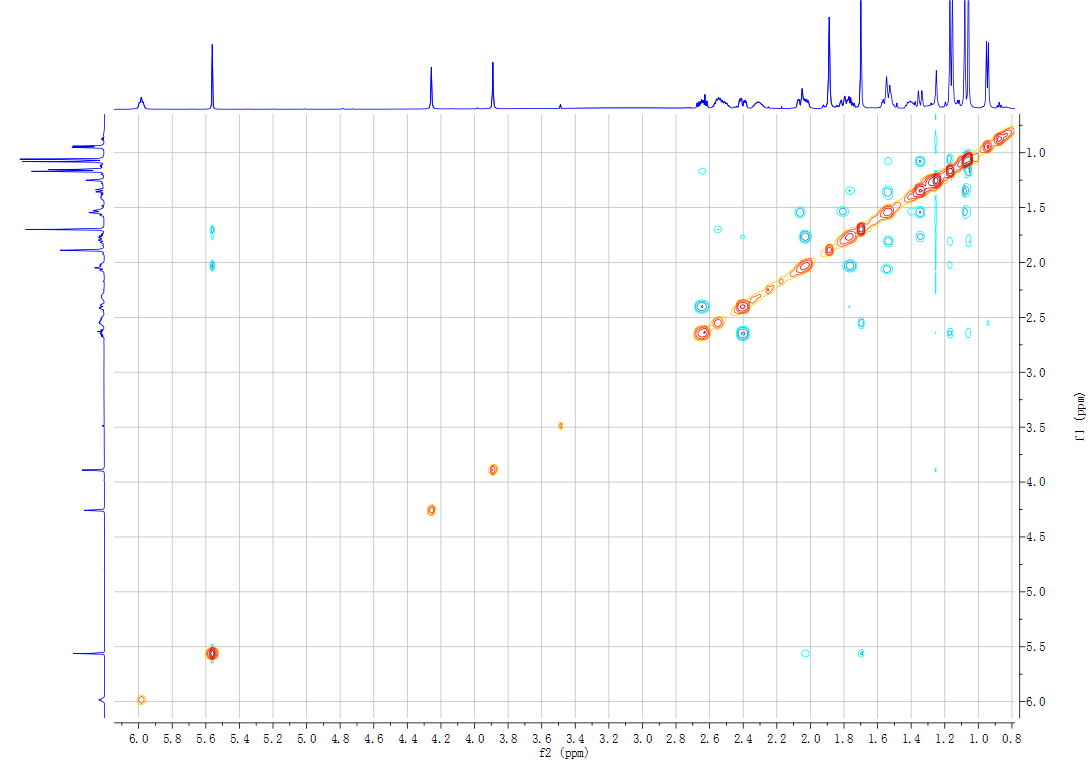


**Figure S37.** ROESY spectrum of Kadcoccitane B (**2**) in CDCl_3_.


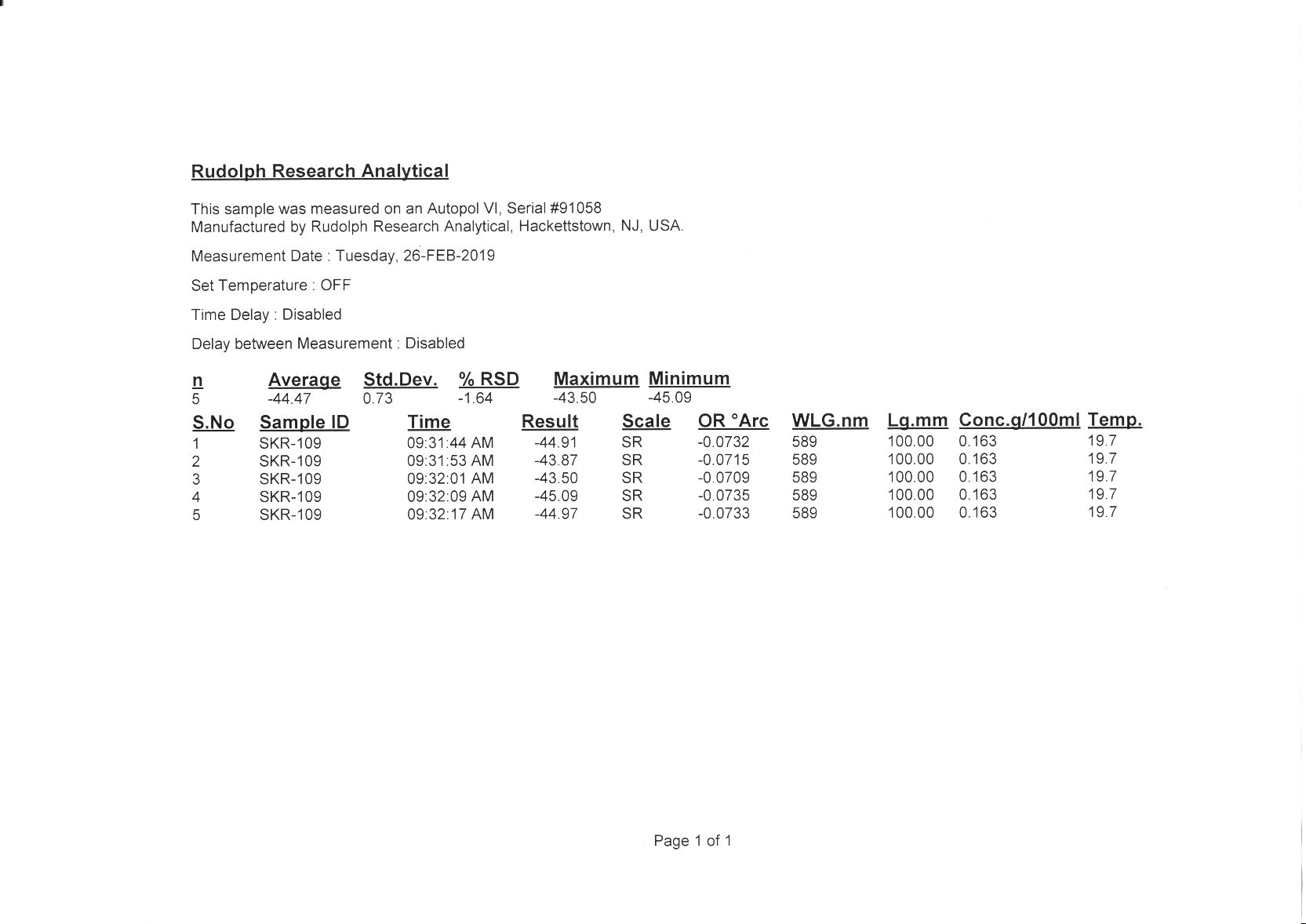


**Figure S38.** Optical rotation spectrum of Kadcoccitane B (**2**).


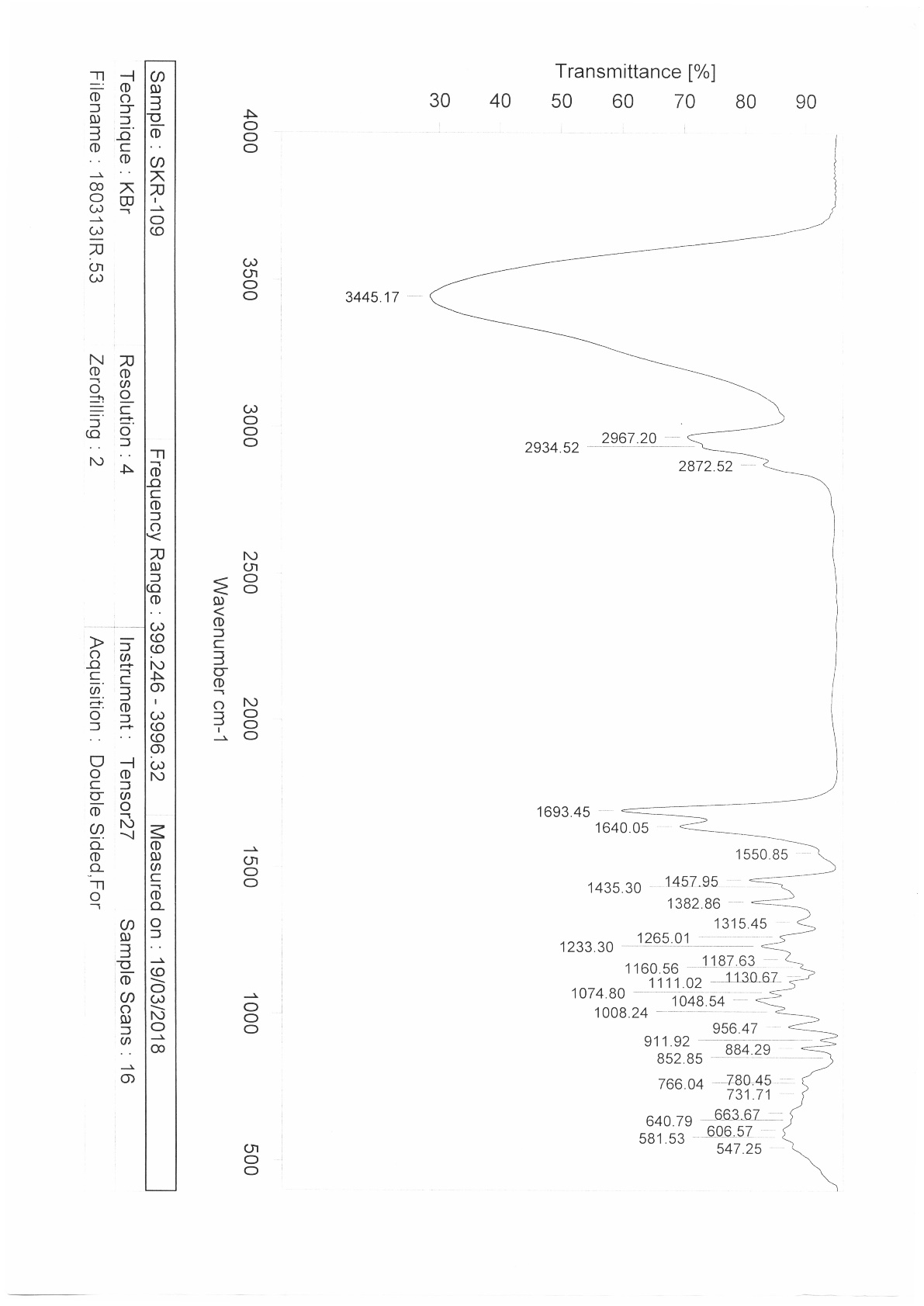


**Figure S39**. IR spectrum of Kadcoccitane B (**2**).


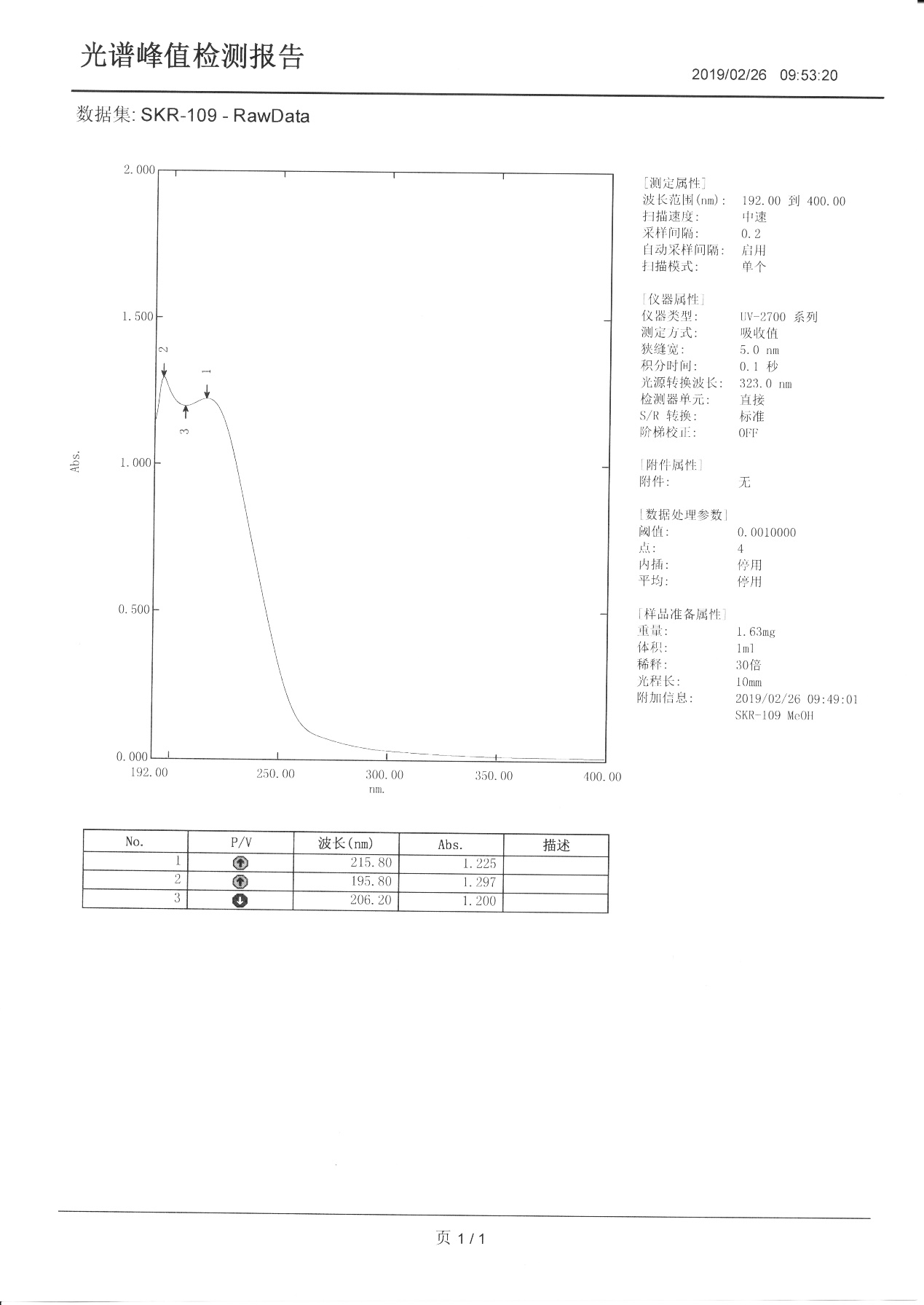


**Figure S40**. UV spectrum of Kadcoccitane B (**2**).


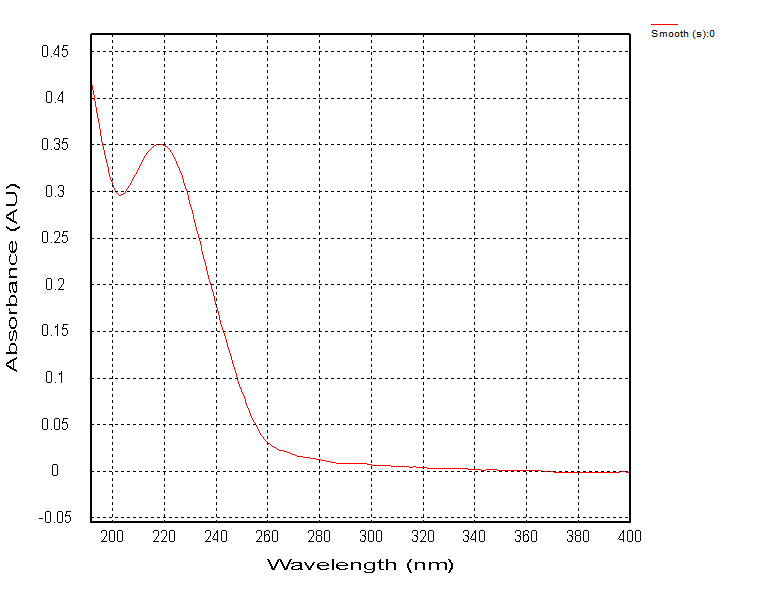


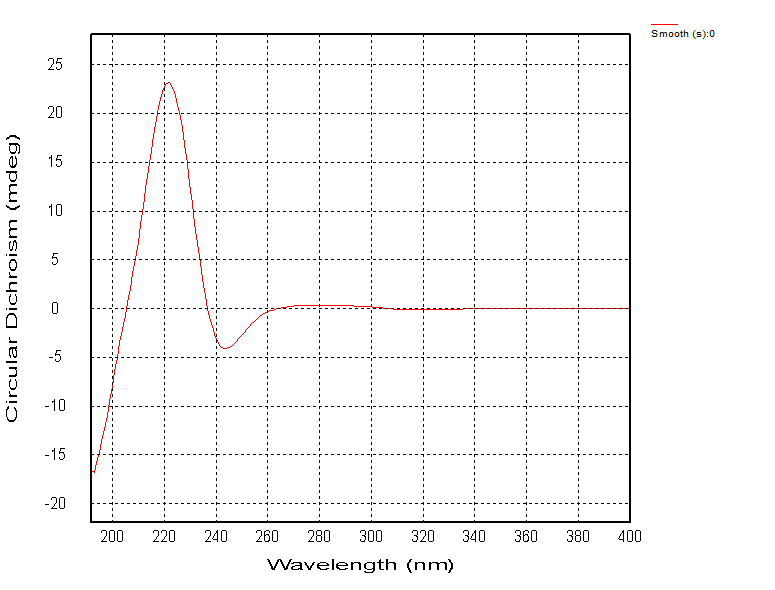


**Figure S41**. ECD (top) and UV (bottom) spectra of Kadcoccitane B (**2**).


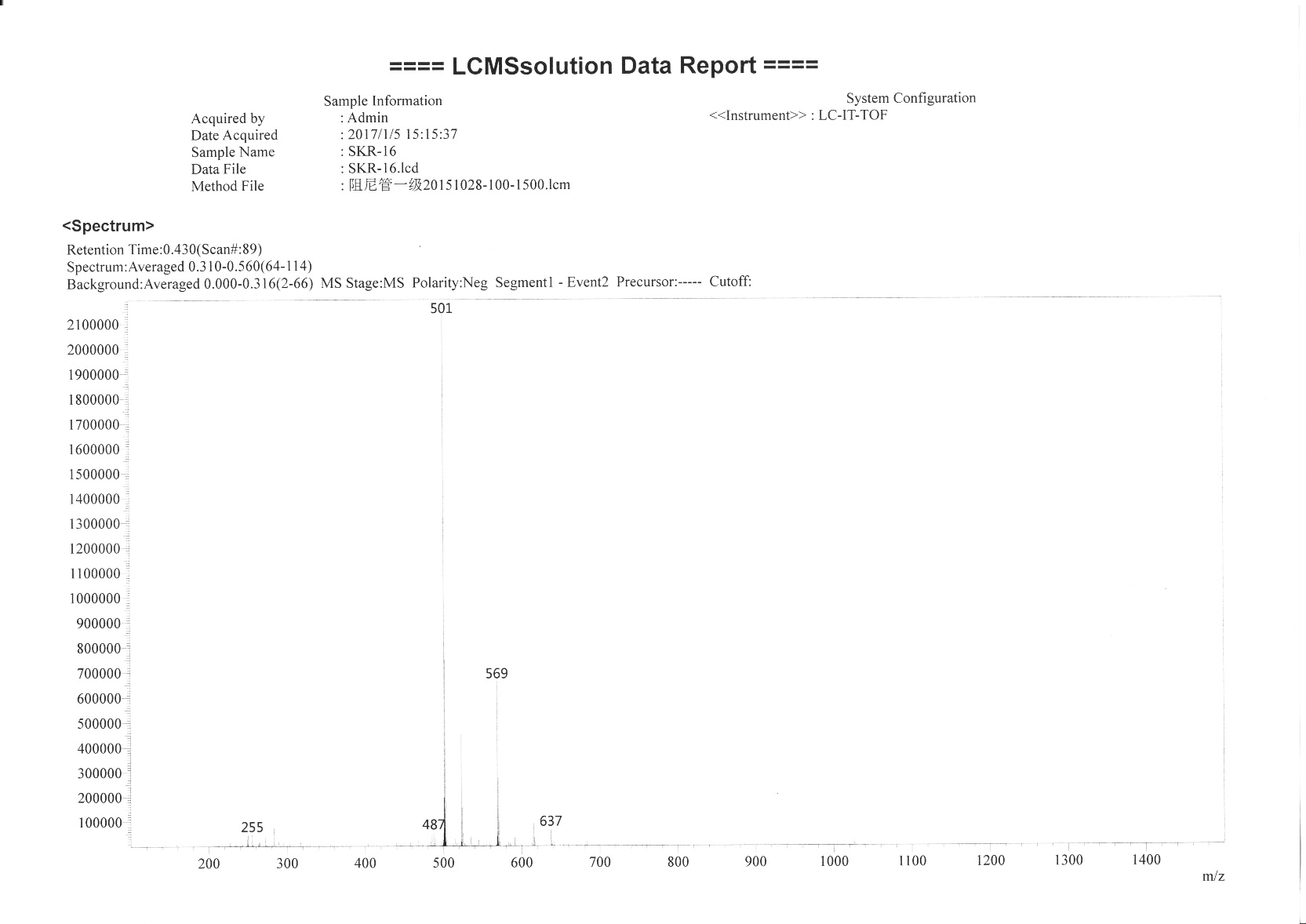


**Figure S42**. ESIMS spectrum of Kadcoccitane C (**3**).


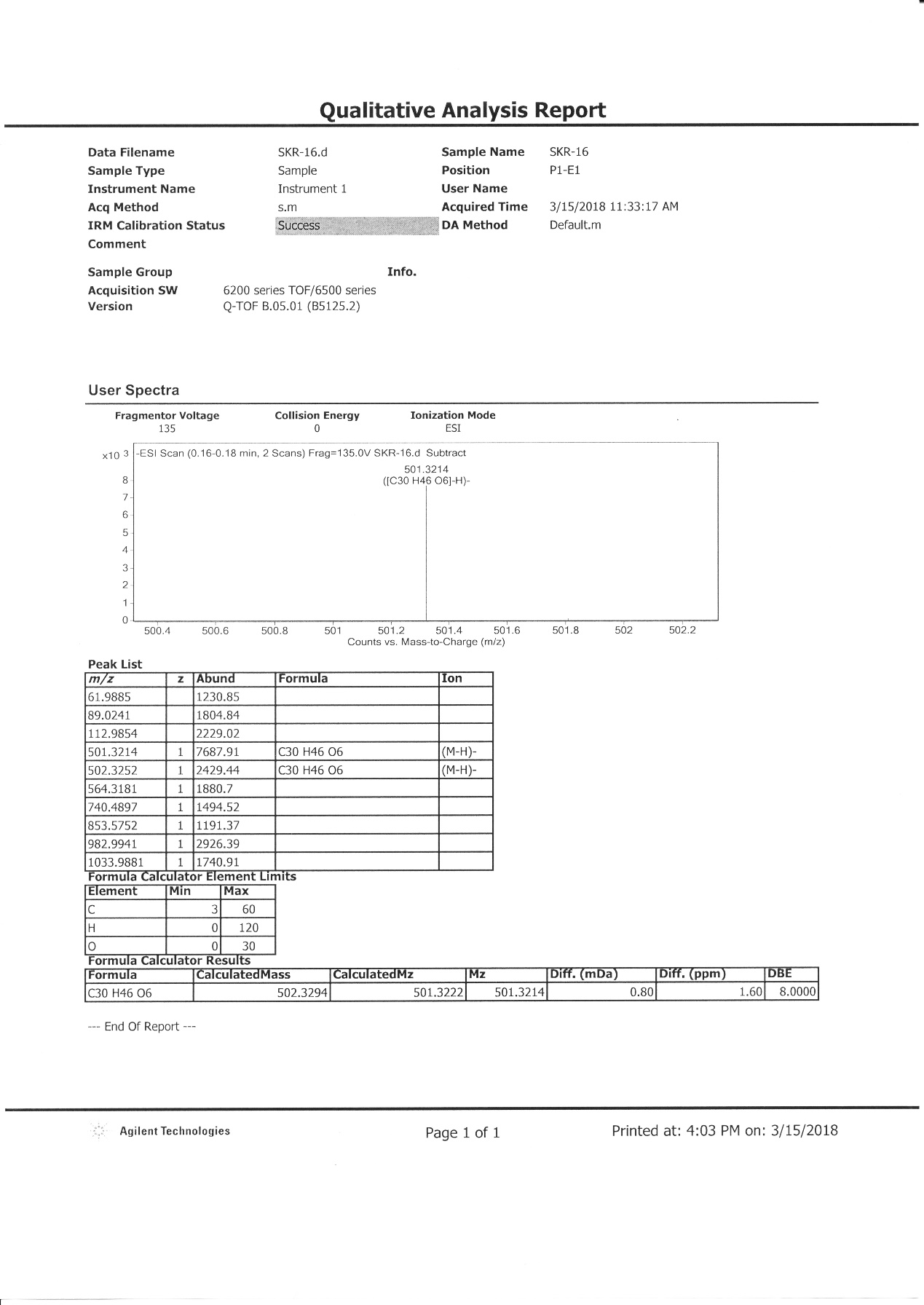


**Figure S43.** HRESIMS spectrum of Kadcoccitane C (**3**).


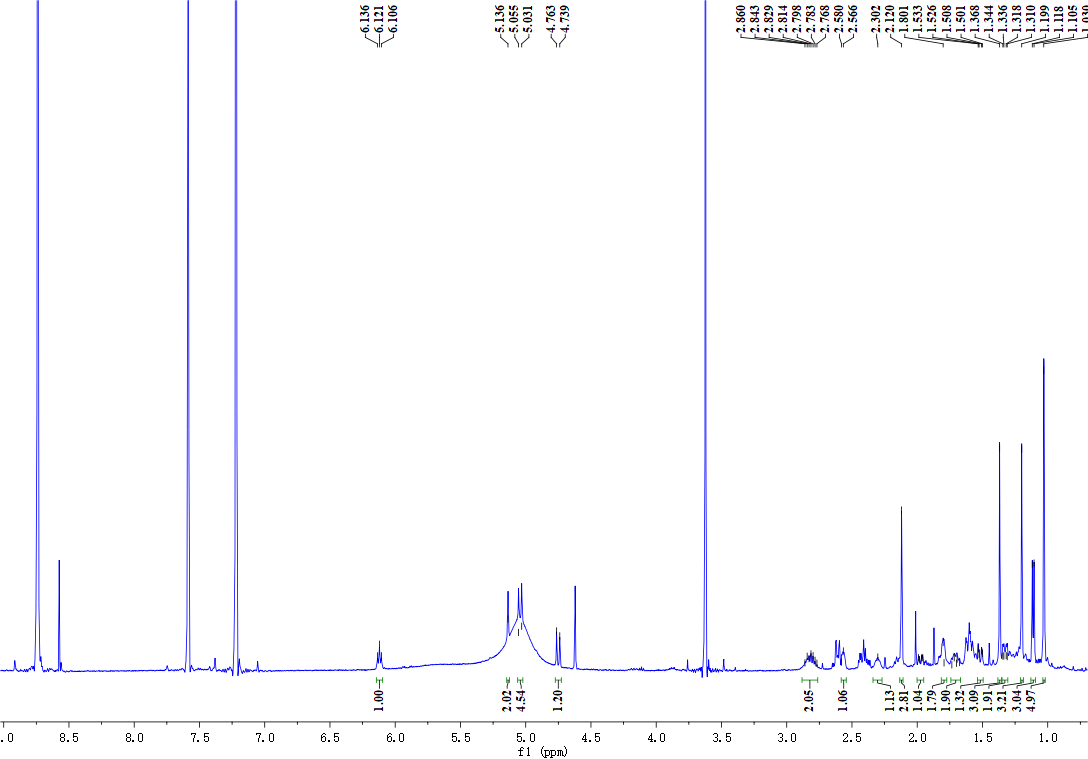


**Figure S44**. ^1^H spectrum of Kadcoccitane C (**3**) in pyridine-*d*_5_.


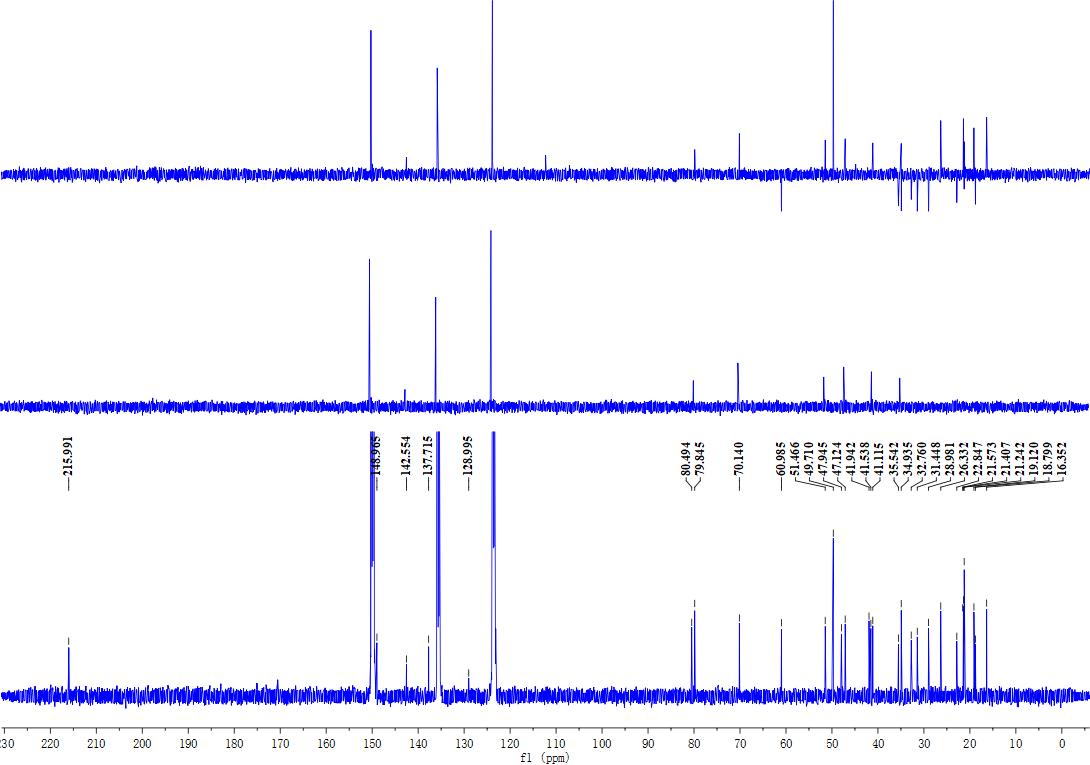


**Figure S45**. ^13^C spectrum of Kadcoccitane C (**3**) in pyridine-*d*_5_.


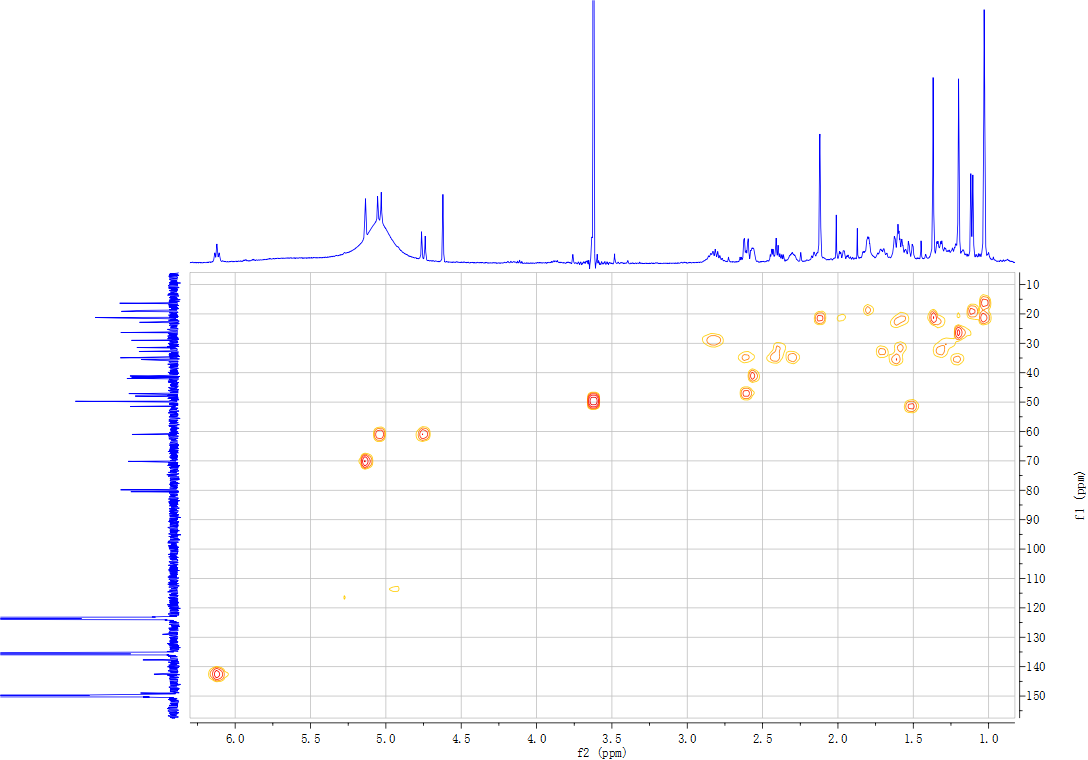


**Figure S46.** HSQC spectrum of Kadcoccitane C (**3**) in pyridine-*d*_5_.


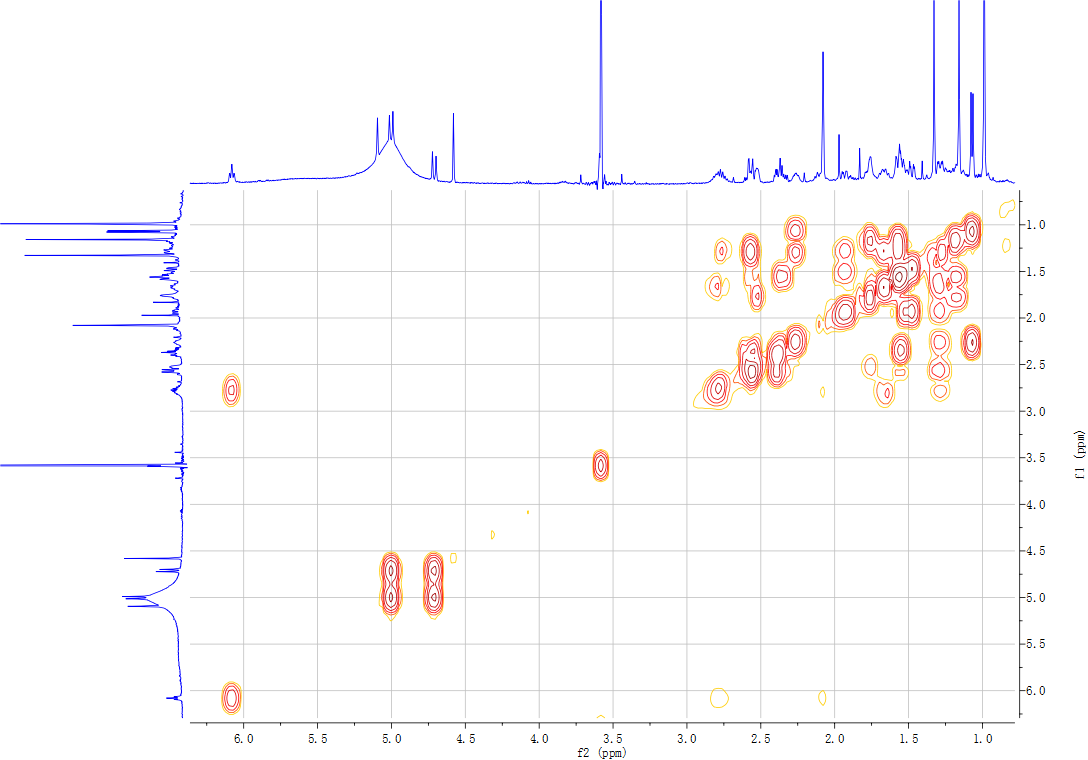


**Figure S47**. ^1^H-^1^H COSY spectrum of Kadcoccitane C (**3**) in pyridine-*d*_5_.


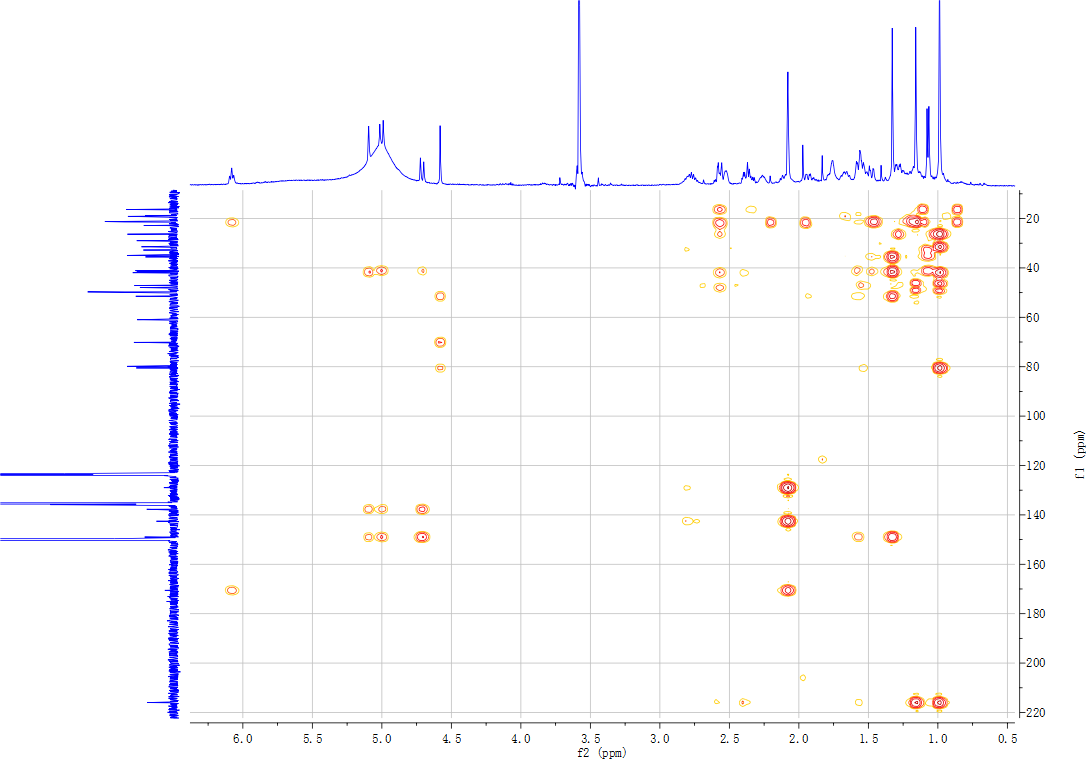


**Figure S48**. HMBC spectrum of Kadcoccitane C (**3**) in pyridine-*d*_5_.


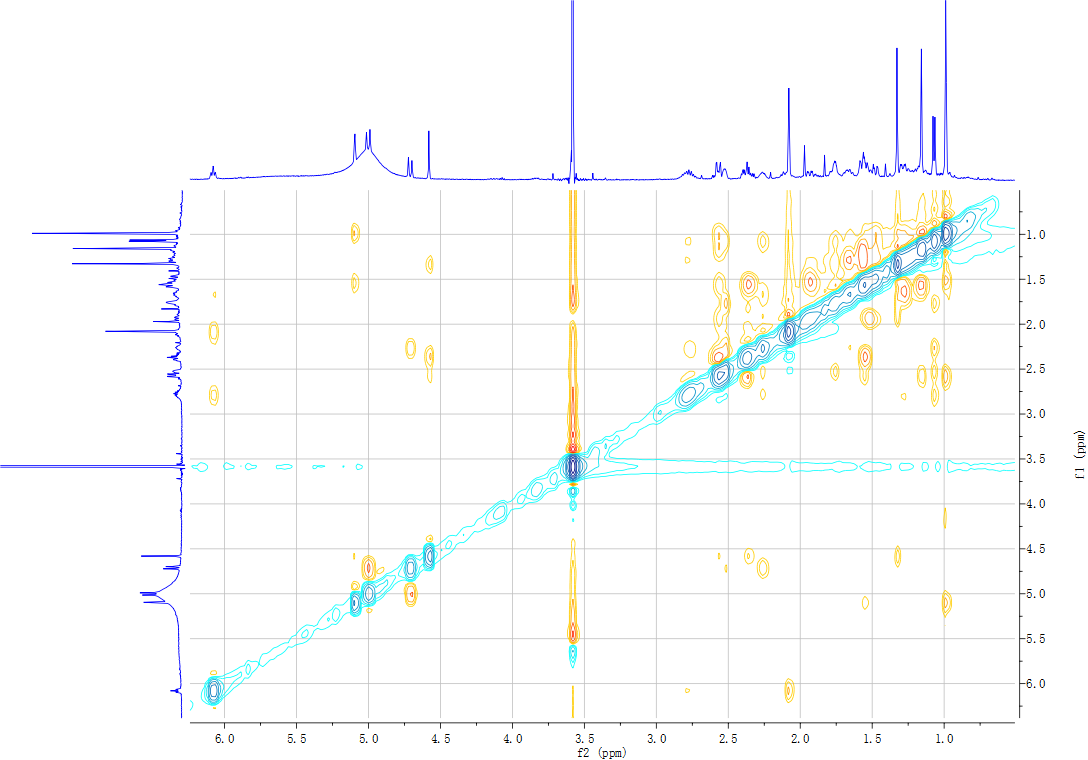


**Figure S49.** ROESY spectrum of Kadcoccitane C (**3**) in pyridine-*d*_5_.


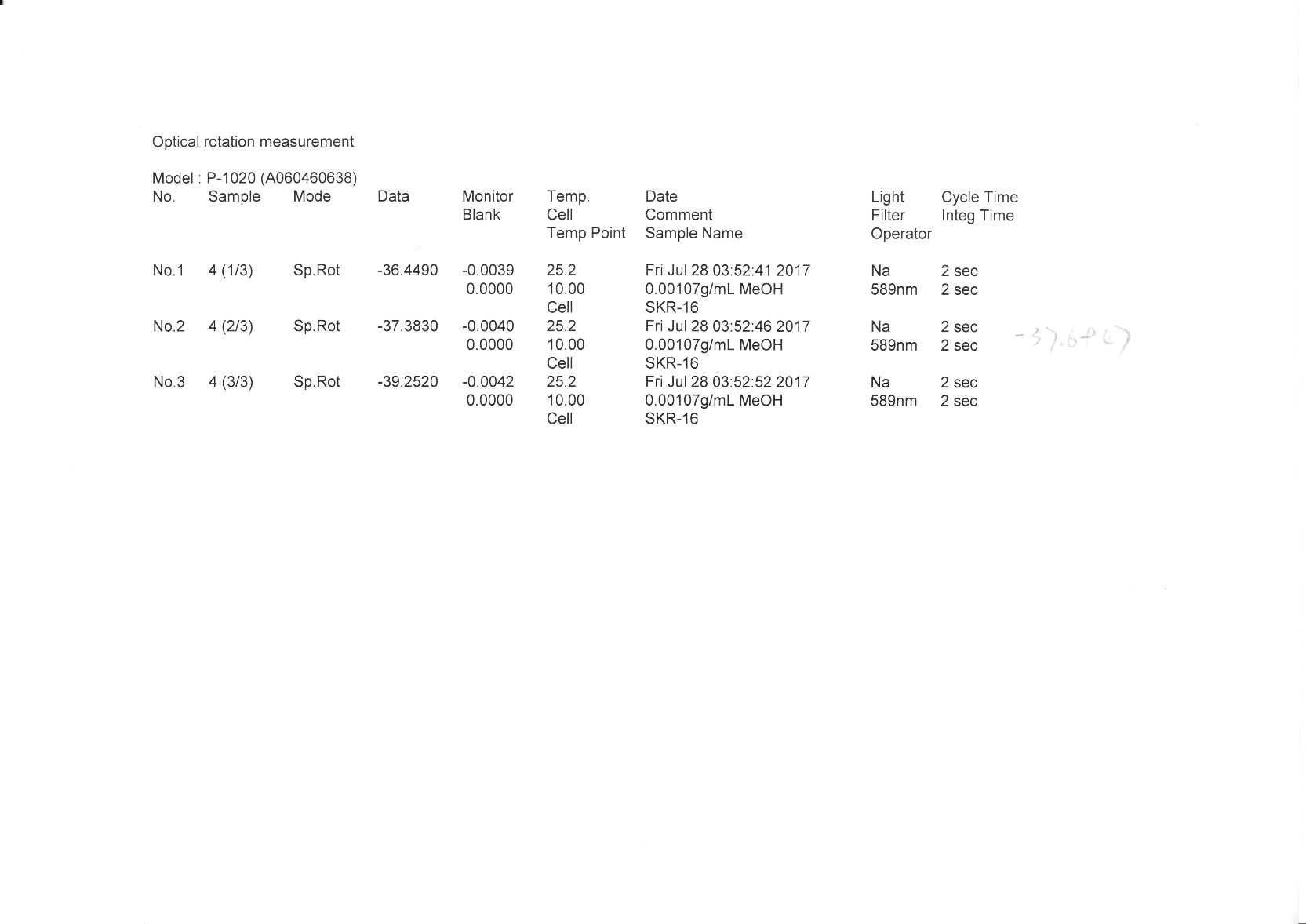


**Figure S50.** Optical rotation spectrum of Kadcoccitane C (**3**).


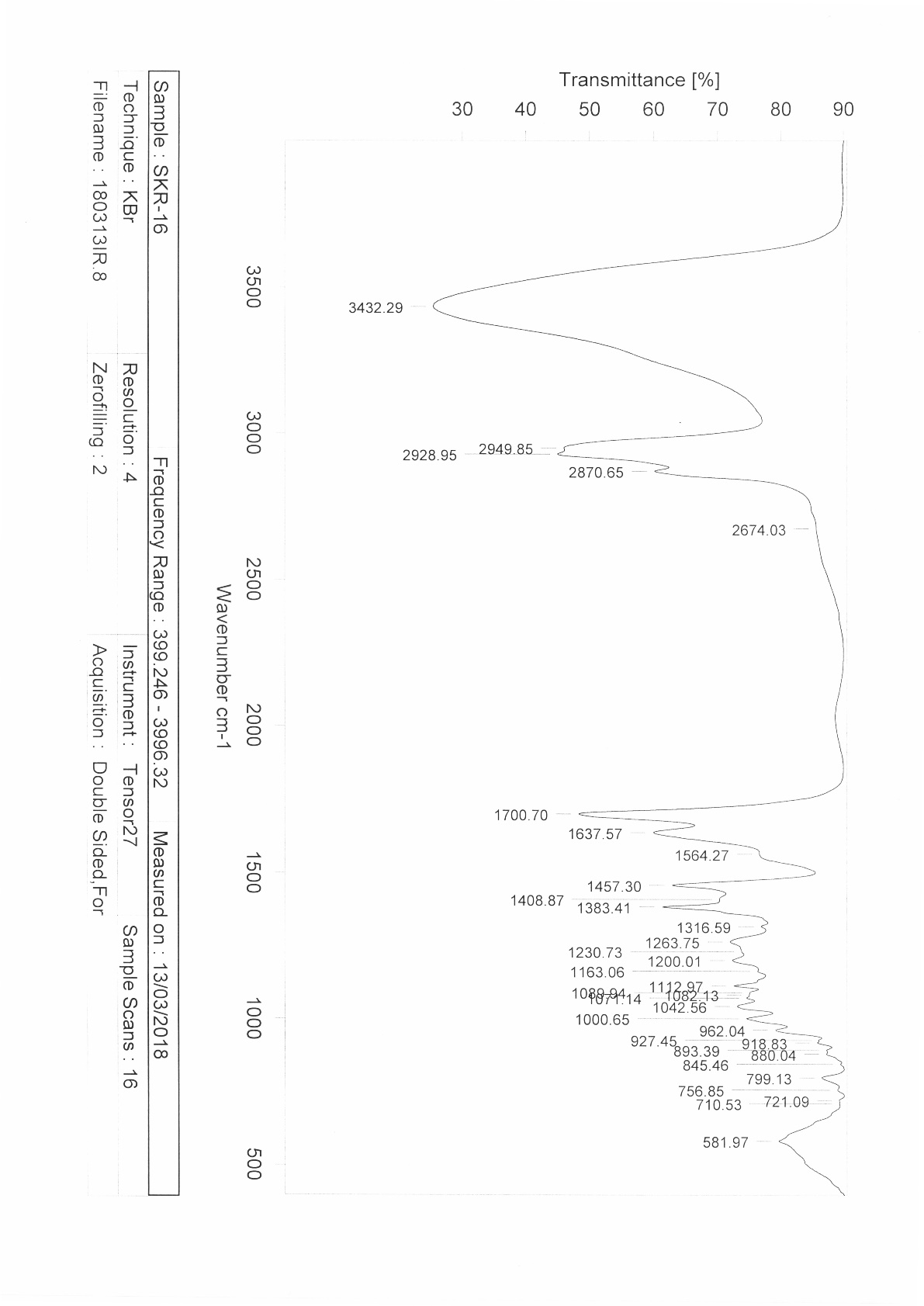


**Figure S51**. IR spectrum of Kadcoccitane C (**3**).


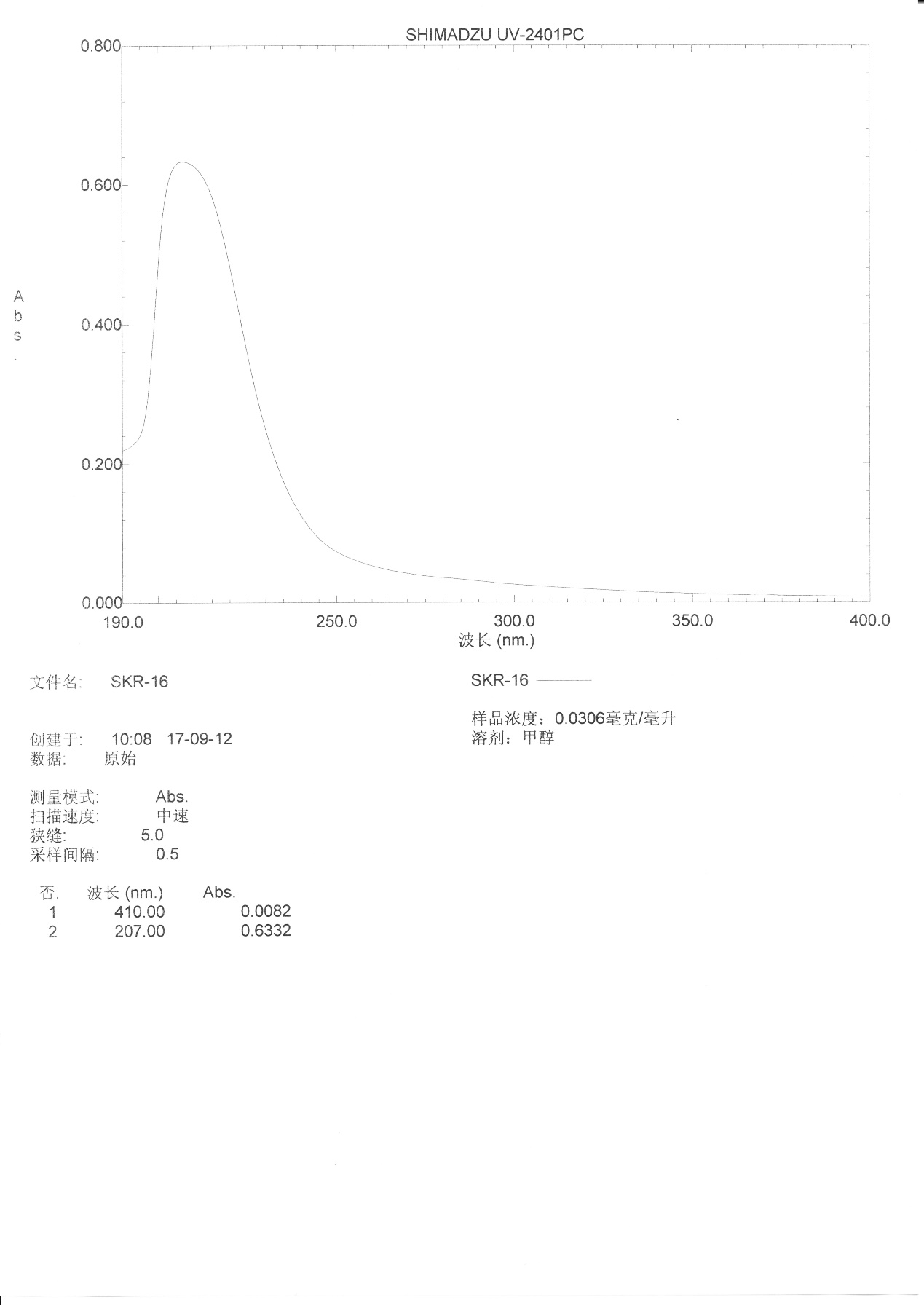


**Figure S52**. UV spectrum of Kadcoccitane C (**3**).


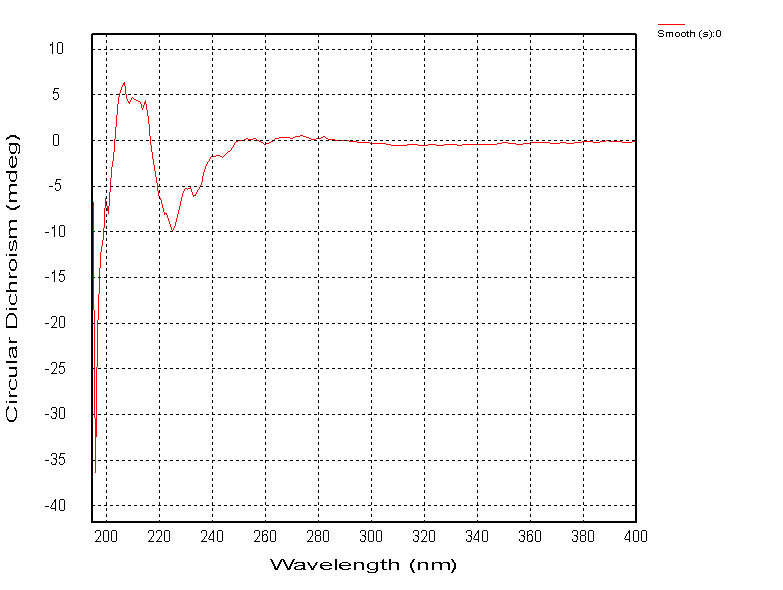


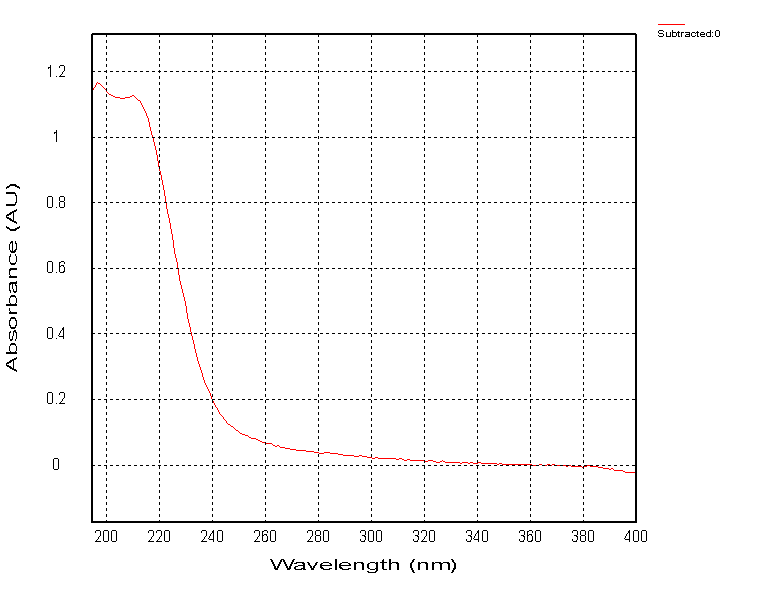


**Figure S53.** ECD (top) and UV (bottom) spectra of Kadcoccitane C (**3**).


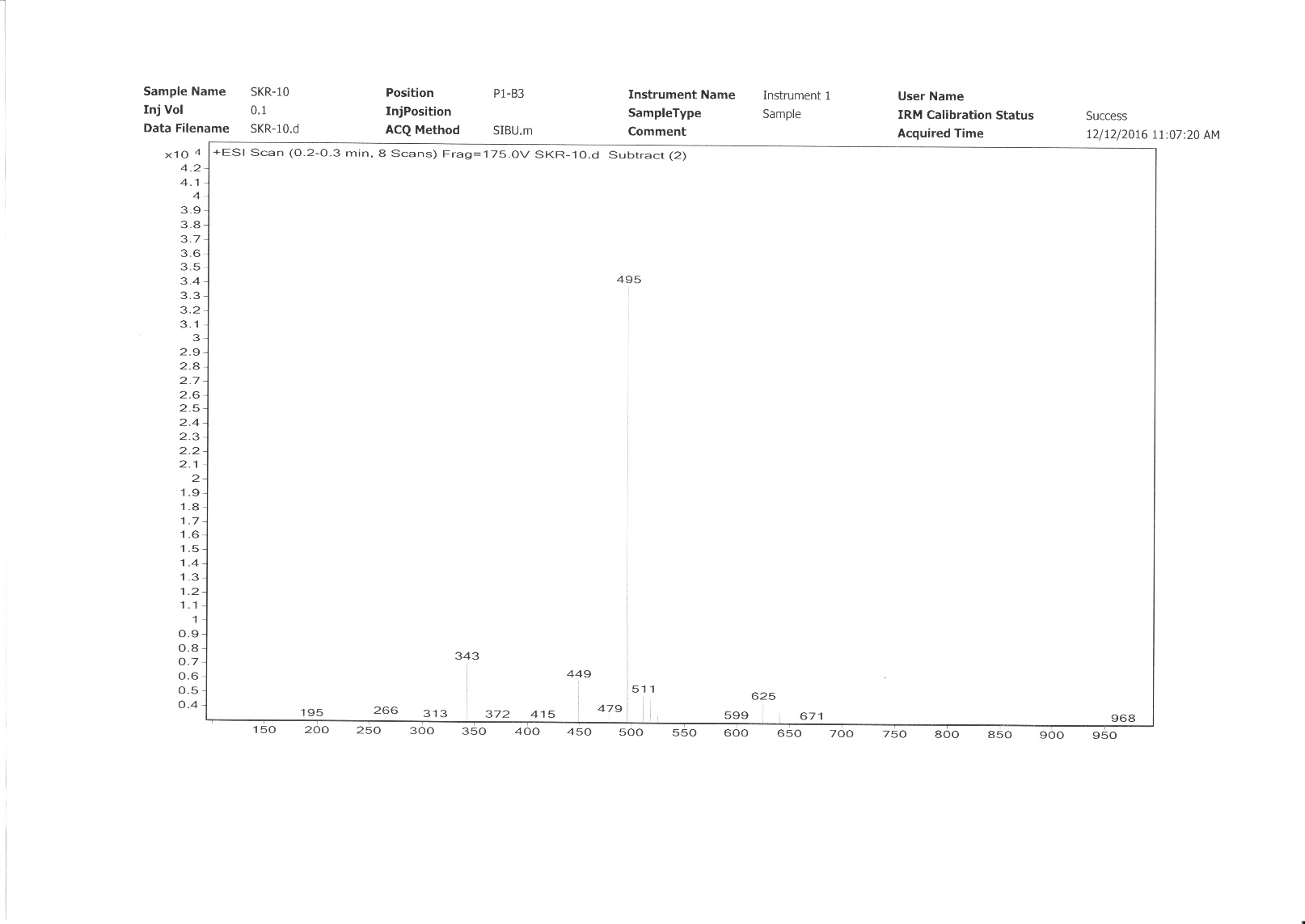


**Figure S54.** ESIMS spectrum of Kadcoccitane D (**4**).


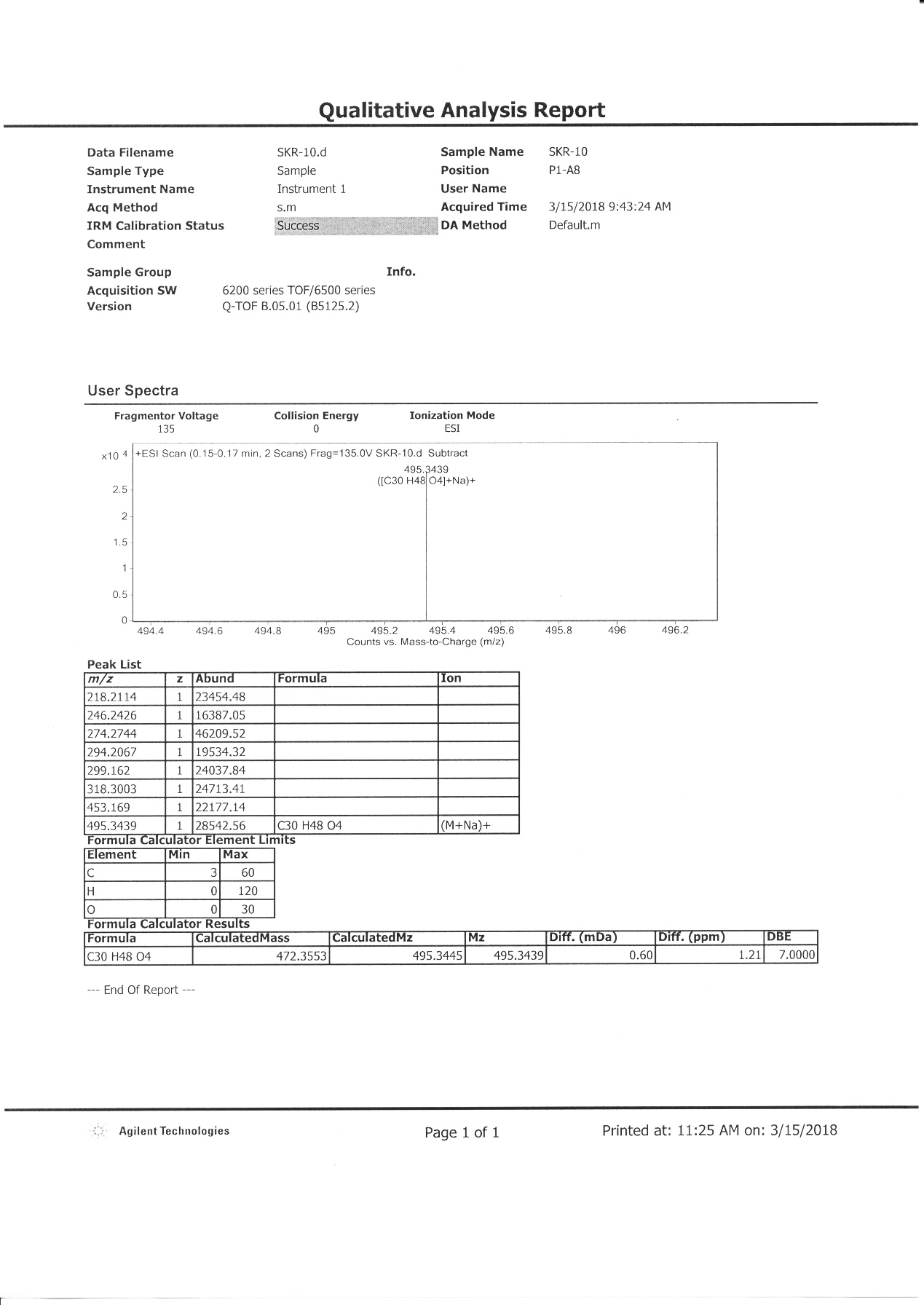


**Figure S55.** HRESIMS spectrum of Kadcoccitane D (**4**)


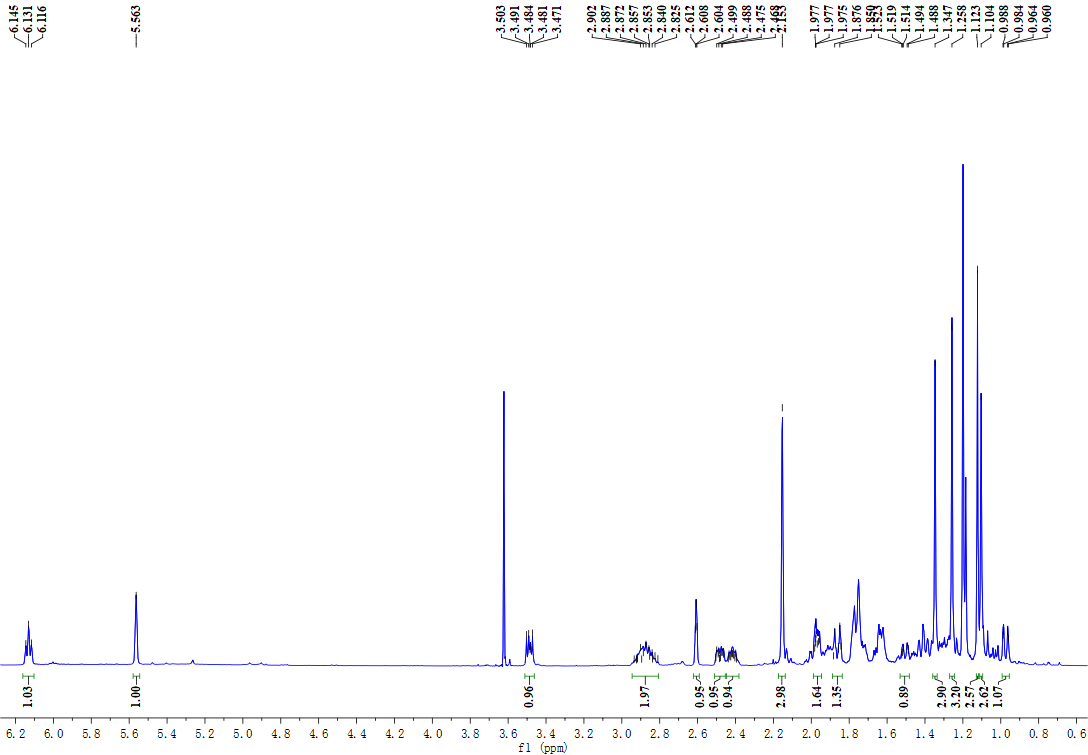


**Figure S56.** ^1^H spectrum of Kadcoccitane D (**4**) in pyridine-d5.


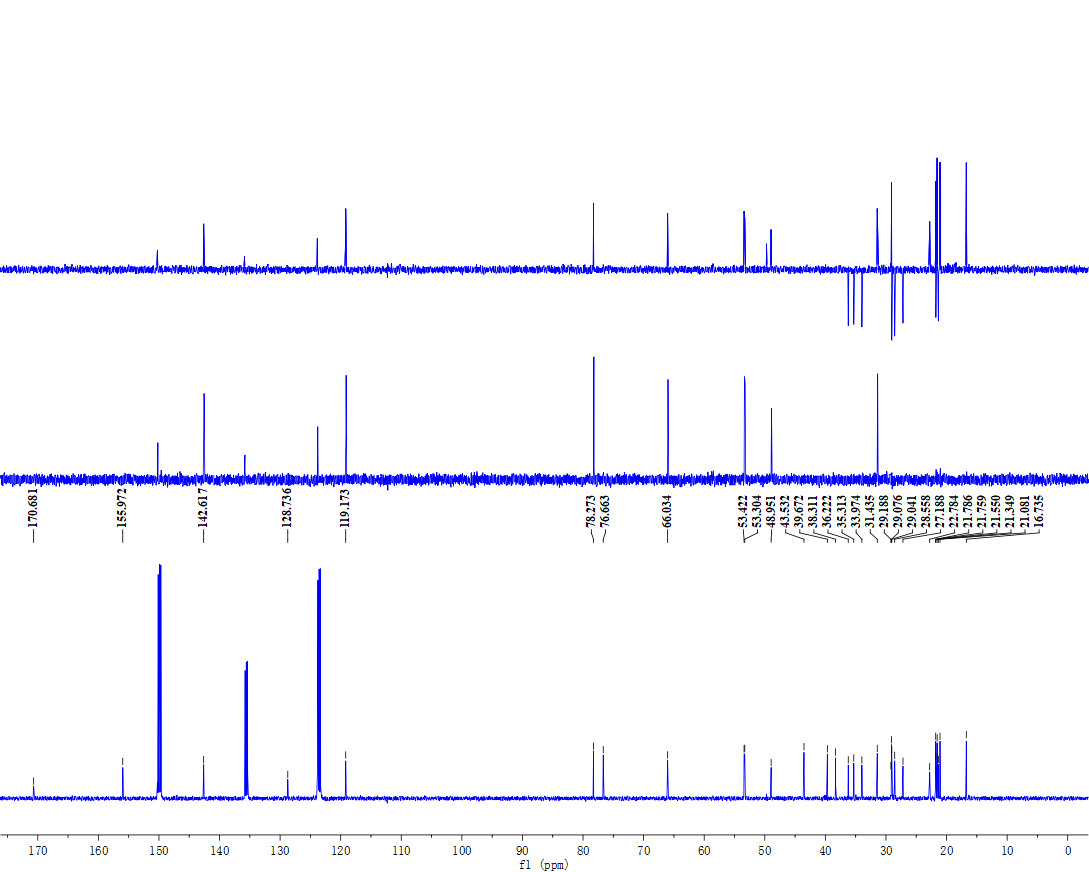


**Figure S57**. ^13^C spectrum of Kadcoccitane D (**4**) in pyridine-*d*_5_.


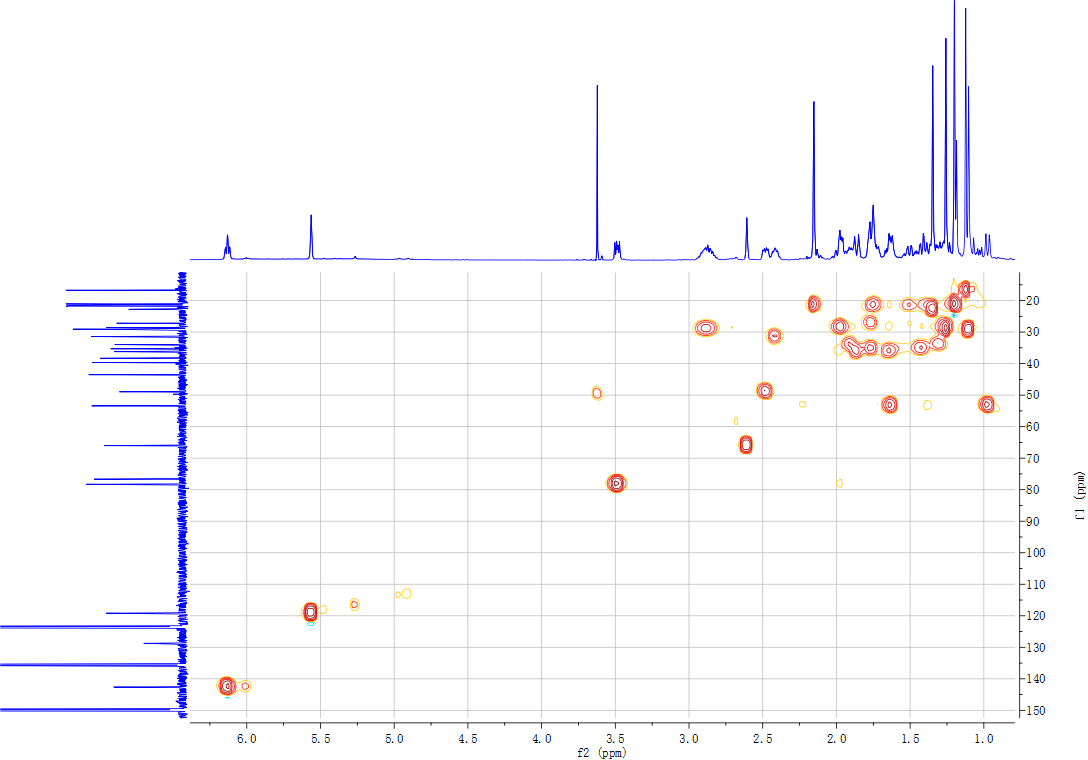


**Figure S58.** HSQC spectrum of Kadcoccitane D (**4**) in pyridine-*d*_5_.


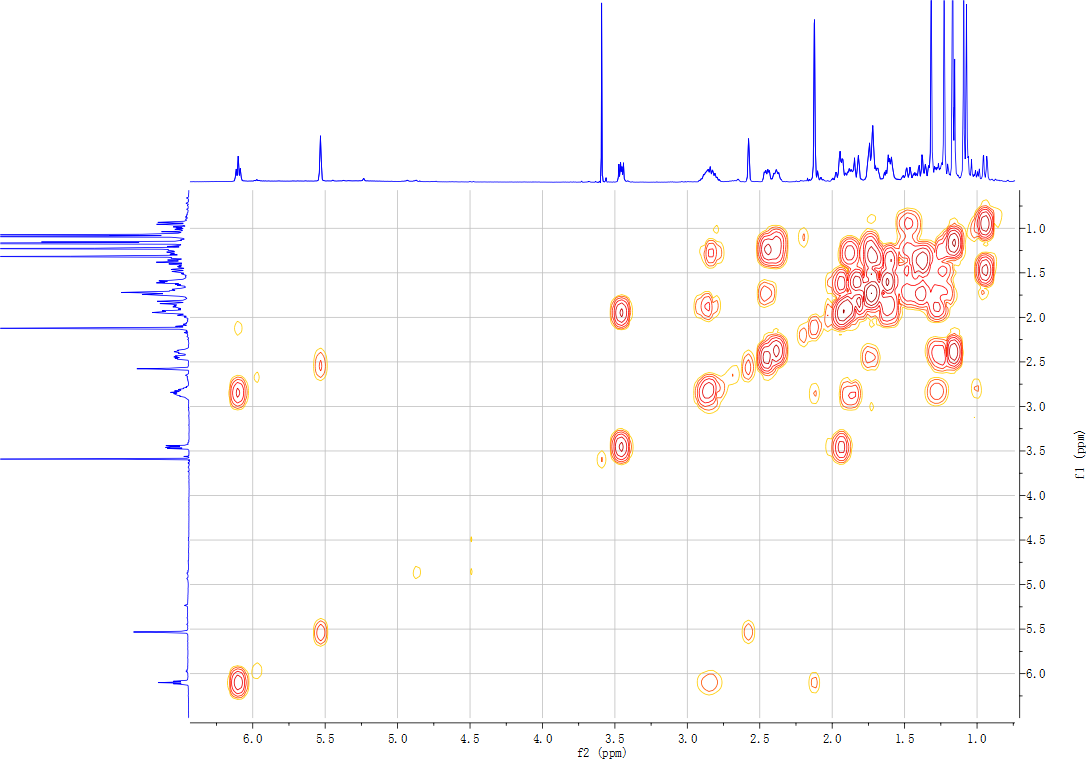


**Figure S59**. ^1^H-^1^H COSY spectrum of Kadcoccitane D (**4**) in pyridine-*d*_5_.


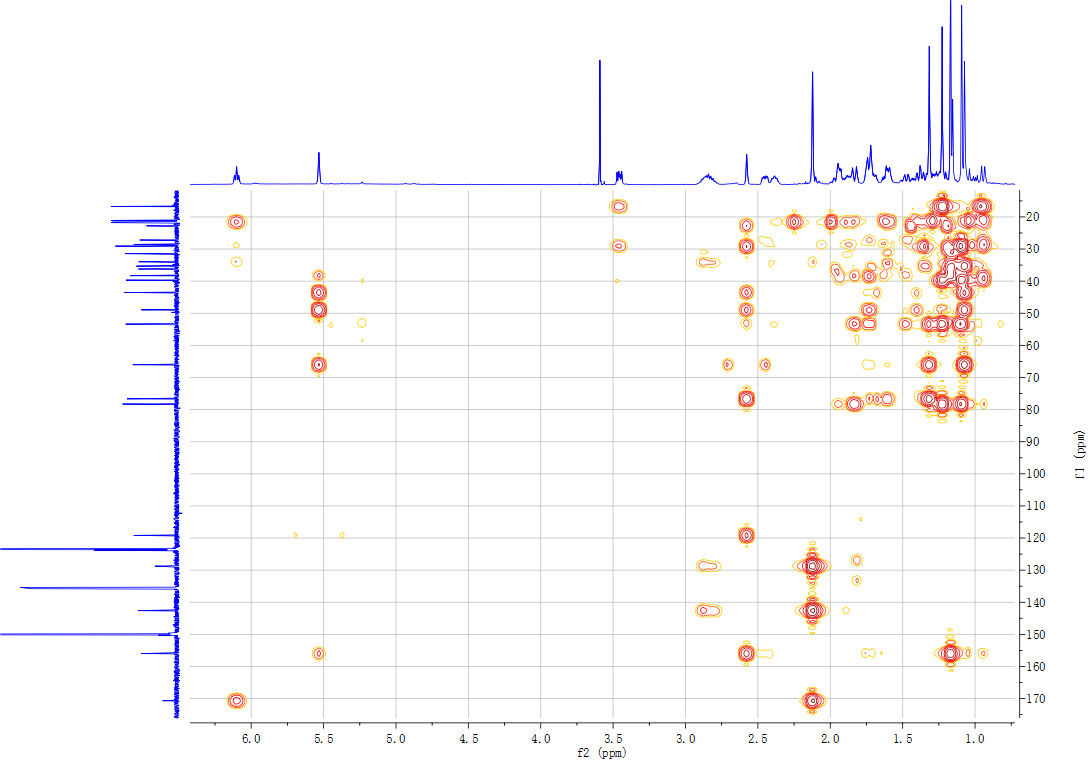


**Figure S60**. HMBC spectrum of Kadcoccitane D (**4**) in pyridine-*d*_5_.


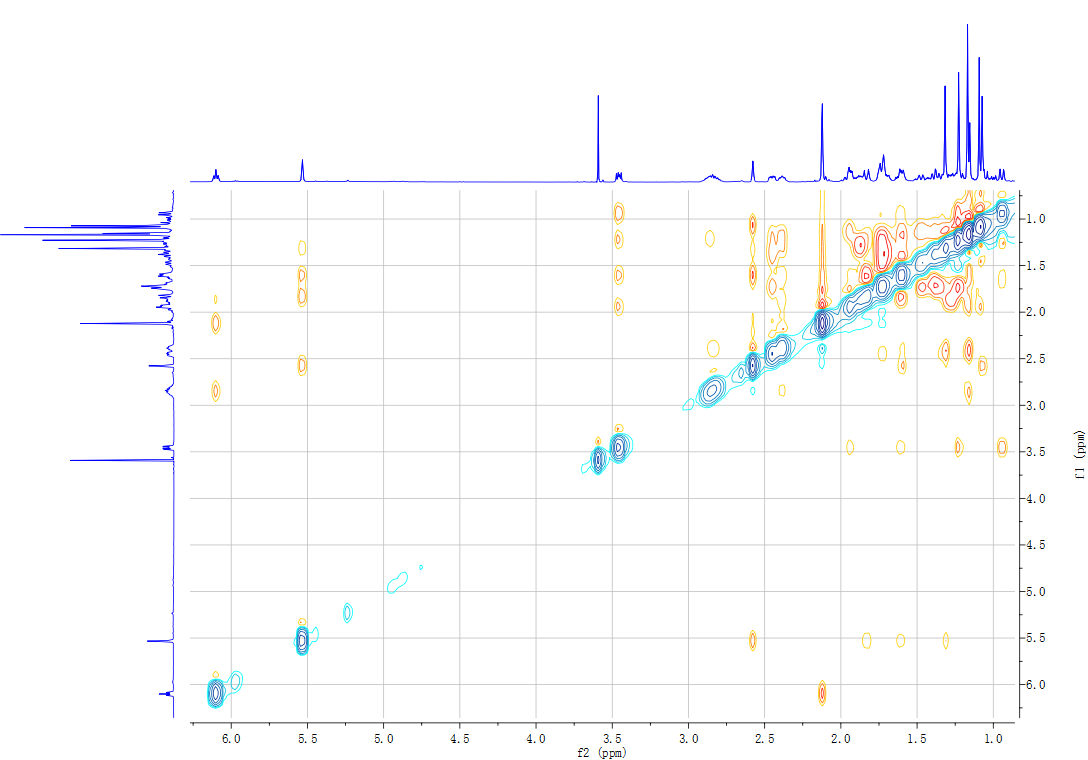


**Figure S61.** ROESY spectrum of Kadcoccitane D (**4**) in pyridine-*d*_5_.


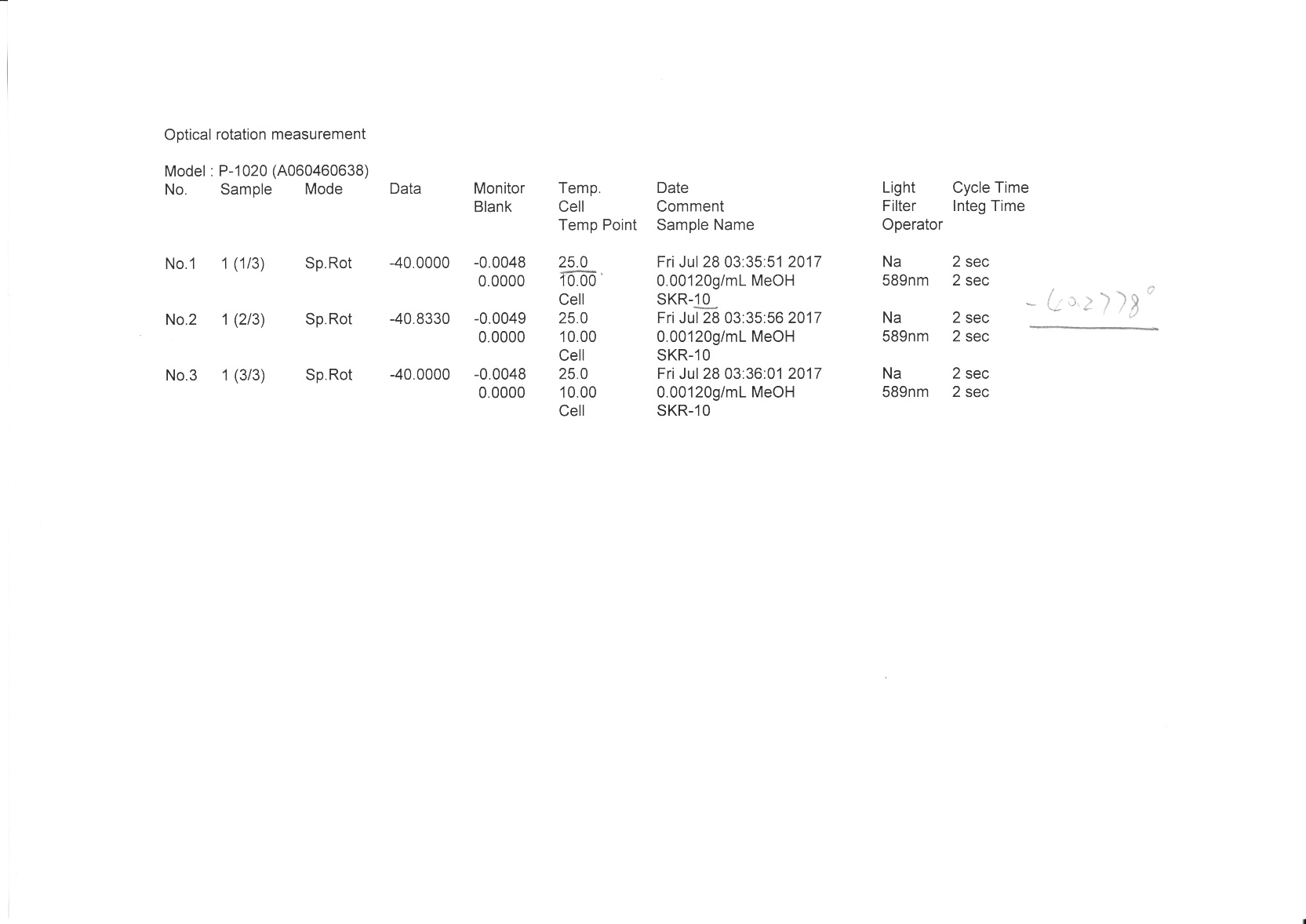


**Figure S62**. Optical rotation spectrum of Kadcoccitane D (**4**).


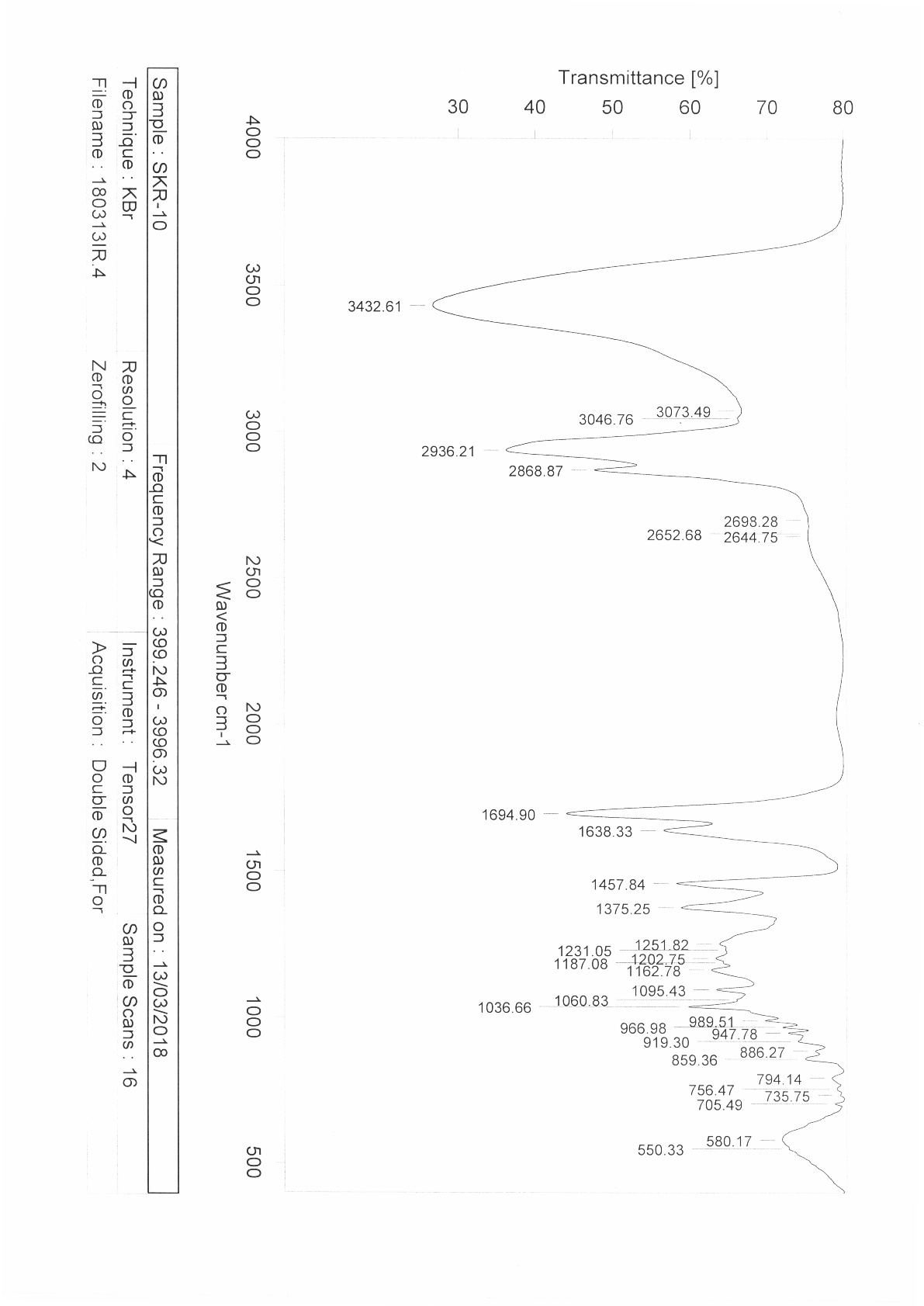


**Figure S63**. IR spectrum of Kadcoccitane D (**4**).


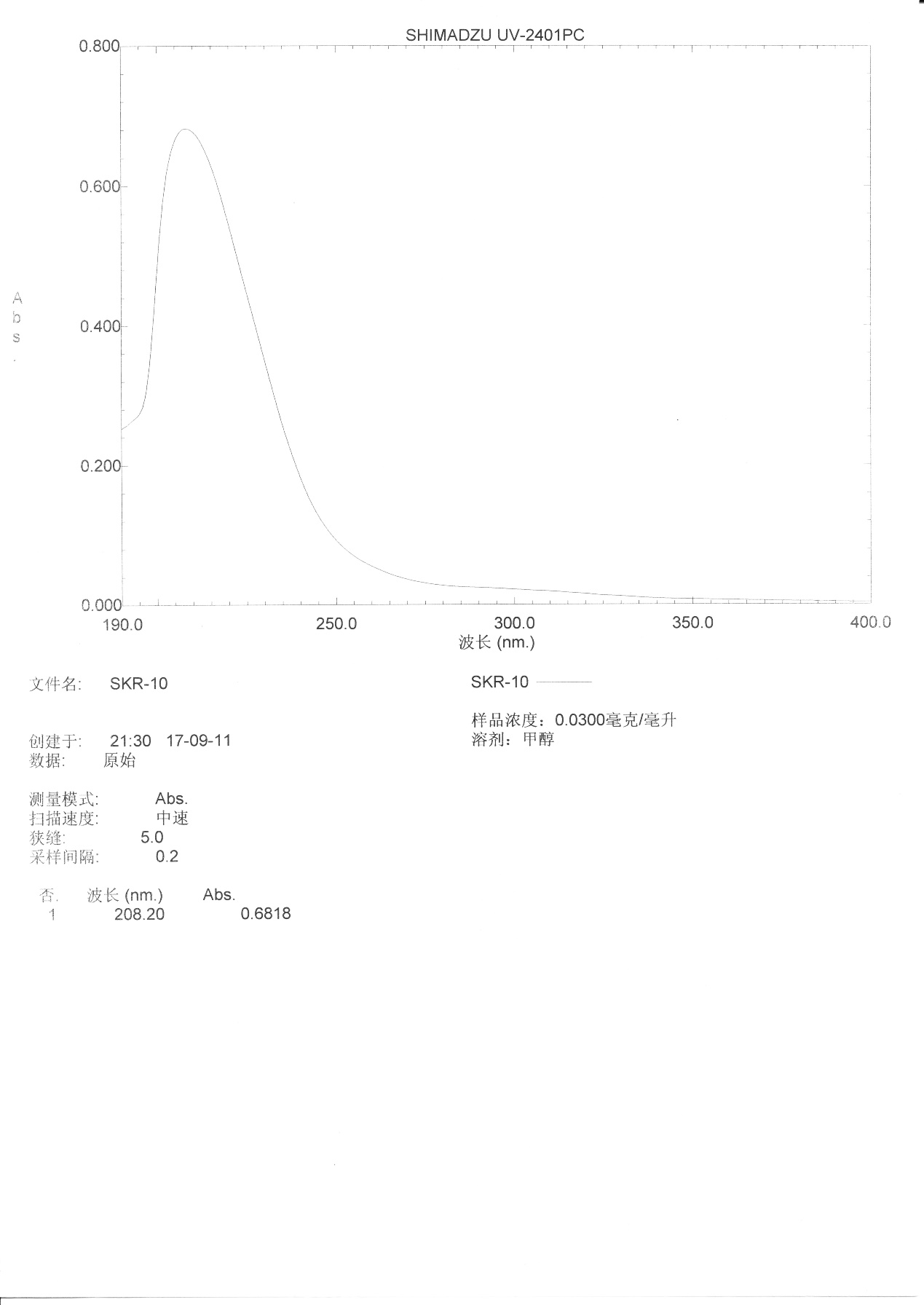


**Figure S64.** UV spectrum of Kadcoccitane D (**4**).


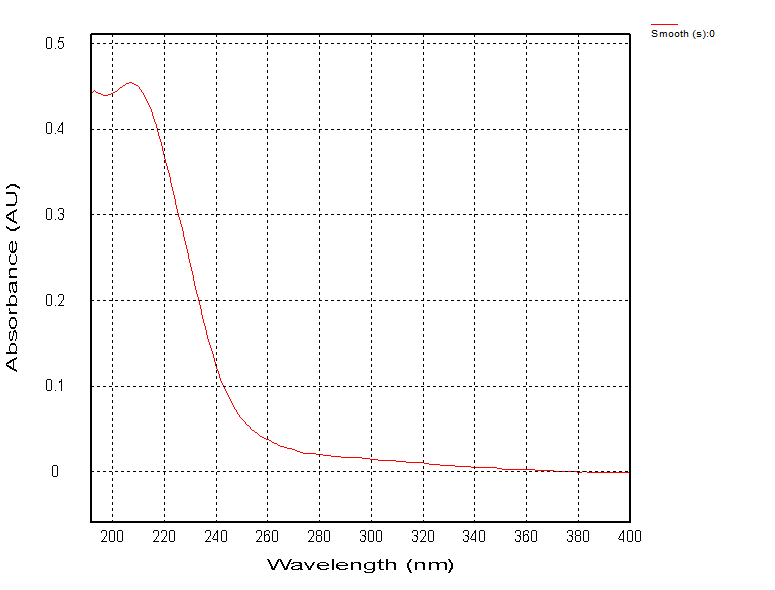


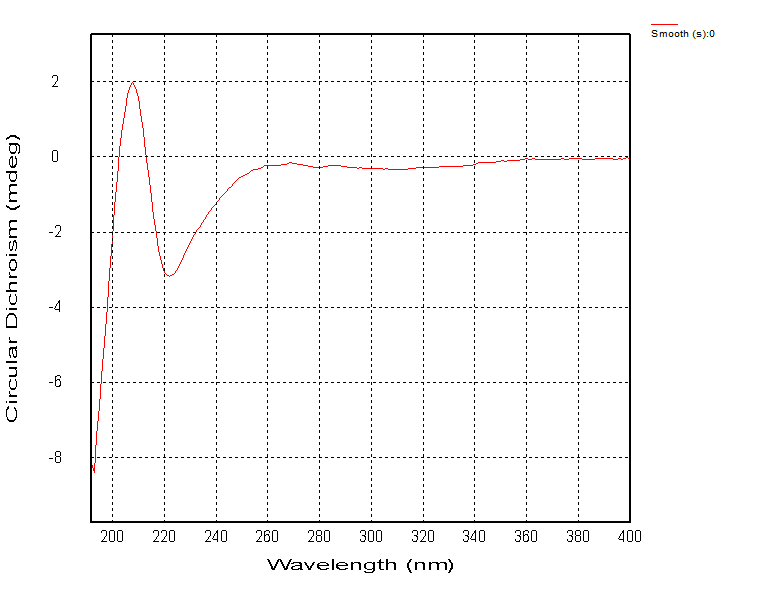


**Figure S65**. ECD (top) and UV (bottom) spectra of Kadcoccitane D (**4**).

1. **Recording curves of influences on the platelet aggregation of rabbits induced by colloid**


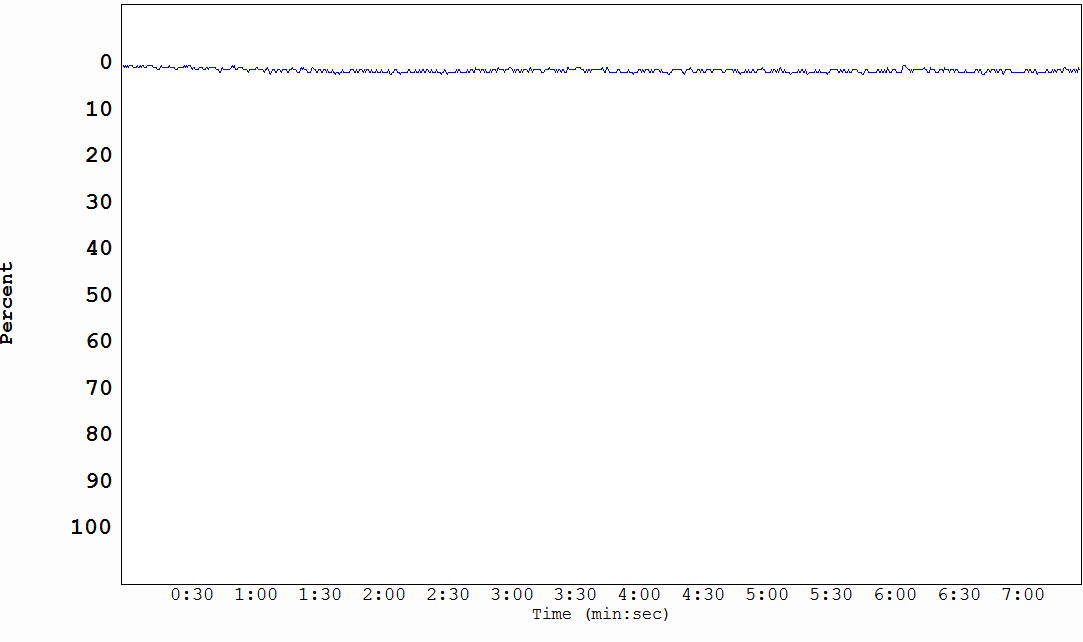


**Figure S66.** The curve of DMSO


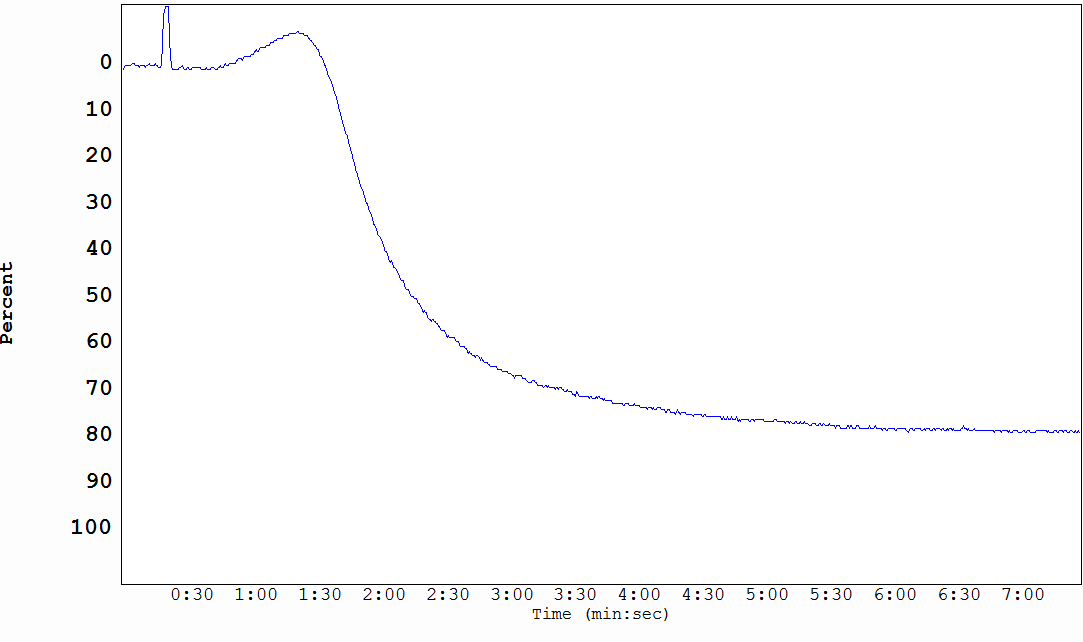


**Figure S67.** The curve of Con.


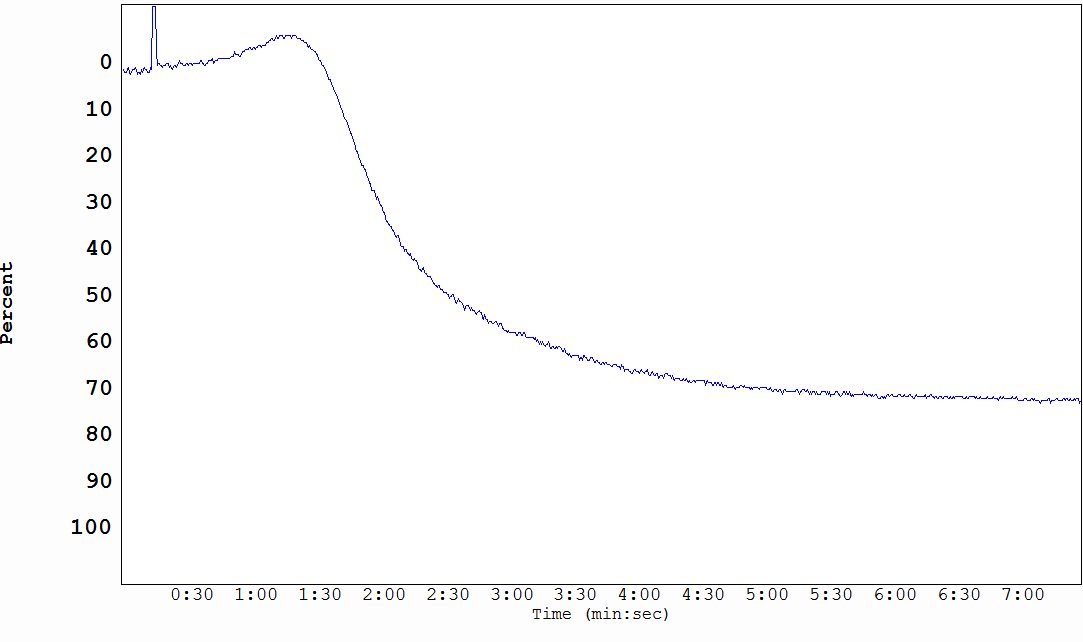


**Figure S68.** The curve of Kadcoccitane A (**1**)


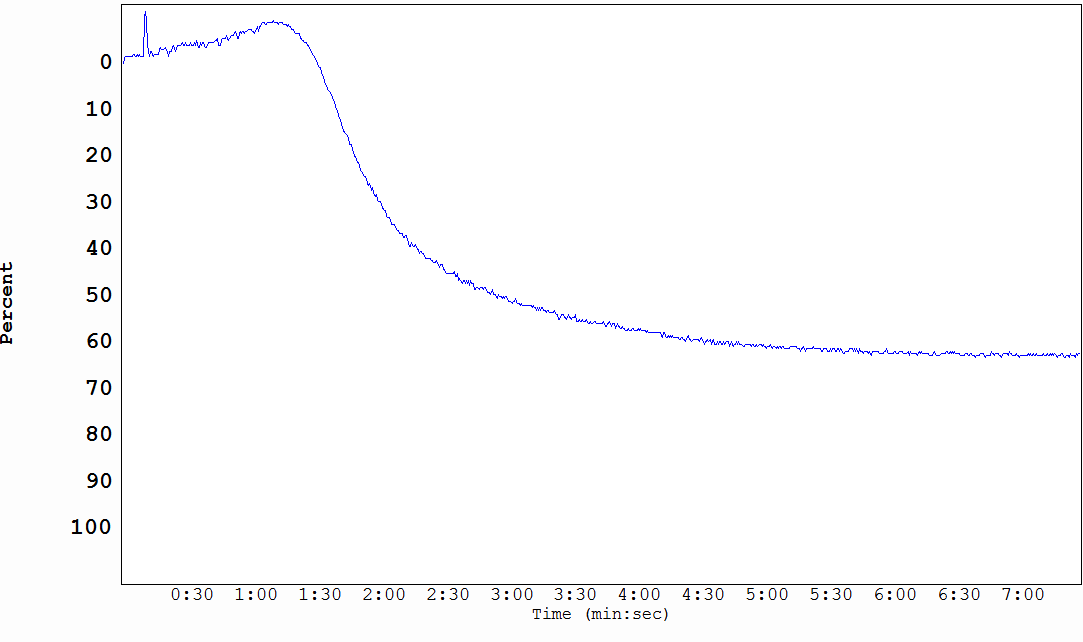


**Figure S69.** The curve of Kadcoccitane C (**3**)


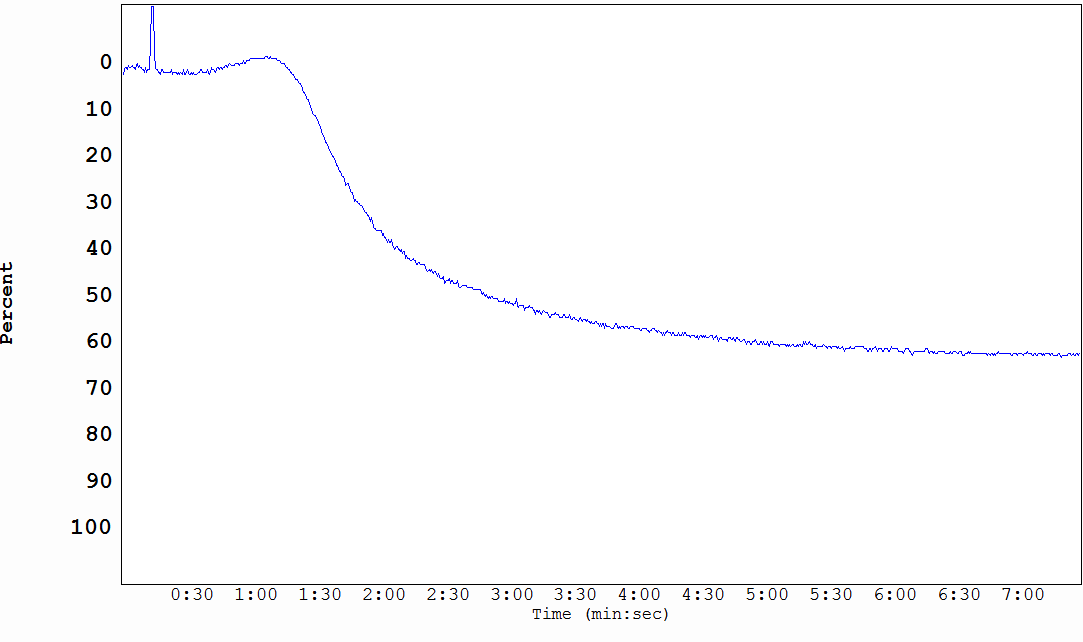


**Figure S70.** The curve of Kadcoccitane D (**4**)

1. **Computational data of 2**

**4.1 General computational data and results of 2**

**Figure S71.** Chemical structures of **2a** of **2b**.

**Table S2.** Experimental and calculated ^13^C-NMR chemical shifts of **2a** and **2b**.

| No. | **2**-*δ_exptl._* | **2a**-*δ_calcd._* | **2b**-*δ_calcd._* | No. | **2**-*δ_exptl._* | **2a**-*δ_calcd._* | **2b**-*δ_calcd._* |
| --- | --- | --- | --- | --- | --- | --- | --- |
| 1 | 35.9 | 37.1 | 37.8 | 16 | 79.4 | 80.0 | 79.9 |
| 2 | 34.5 | 37.3 | 36.6 | 17 | 136.5 | 134.8 | 134.4 |
| 3 | 216.8 | 212.1 | 214.0 | 18 | 11.7 | 14.4 | 14.2 |
| 4 | 47.7 | 50.7 | 49.3 | 19 | 20.3 | 22.1 | 24.2 |
| 5 | 52.6 | 54.5 | 52.4 | 20 | 34.0 | 37.3 | 36.6 |
| 6 | 19.7 | 21.4 | 22.8 | 21 | 18.9 | 20.7 | 21.8 |
| 7 | 36.6 | 39.1 | 37.8 | 22 | 34.3 | 39.1 | 34.3 |
| 8 | 92.3 | 90.2 | 90.4 | 23 | 28.2 | 33.0 | 32.8 |
| 9 | 153.7 | 155.8 | 153.0 | 24 | 145.9 | 147.6 | 148.6 |
| 10 | 37.3 | 41.0 | 41.6 | 25 | 126.7 | 127.4 | 126.0 |
| 11 | 129.3 | 125.6 | 128.1 | 26 | 172.6 | 163.3 | 162.3 |
| 12 | 88.3 | 87.5 | 86.7 | 27 | 20.8 | 22.6 | 23.4 |
| 13 | 136.1 | 139.5 | 139.3 | 28 | 12.2 | 14.0 | 13.9 |
| 14 | 58.6 | 58.2 | 58.1 | 29 | 22.0 | 23.1 | 23.7 |
| 15 | 78.1 | 78.1 | 77.7 | 30 | 26.5 | 26.3 | 28.1 |

**Table S3.** Experimental and calculated ^1^H NMR chemical shifts of **2a** and **2b**.

| No. | **2**-*δ_exptl._* | **2a**-*δ_calcd._* | **2b**-*δ_calcd._* | No. | **2**-*δ_exptl._* | **2a**-*δ_calcd._* | **2b**-*δ_calcd._* |
| --- | --- | --- | --- | --- | --- | --- | --- |
| 1a | 2.03 | 2.50 | 2.05 | 19 | 1.17 | 1.26 | 1.34 |
| 1b | 1.77 | 1.52 | 1.84 | 20 | 2.55 | 2.50 | 2.50 |
| 2a | 2.65 | 3.02 | 2.44 | 21 | 0.95 | 1.07 | 0.97 |
| 2b | 2.40 | 1.91 | 2.40 | 22a | 1.53 | 1.60 | 1.58 |
| 5 | 1.35 | 1.16 | 1.53 | 22b | 1.41 | 1.33 | 1.48 |
| 6a | 1.81 | 2.13 | 2.01 | 23a | 2.31 | 2.07 | 2.00 |
| 6b | 1.54 | 1.32 | 1.31 | 23b | 2.52 | 3.09 | 2.57 |
| 7a | 2.06 | 2.24 | 2.09 | 24 | 5.98 | 6.36 | 6.70 |
| 7b | 1.54 | 1.35 | 1.42 | 27 | 1.89 | 2.01 | 2.01 |
| 11 | 5.56 | 5.86 | 5.82 | 28 | 1.15 | 1.11 | 1.12 |
| 15 | 3.89 | 3.53 | 3.78 | 29 | 1.06 | 1.02 | 0.96 |
| 16 | 4.26 | 4.02 | 4.14 | 30 | 1.08 | 0.94 | 0.94 |
| 18 | 1.70 | 1.94 | 1.78 |  |  |  |  |


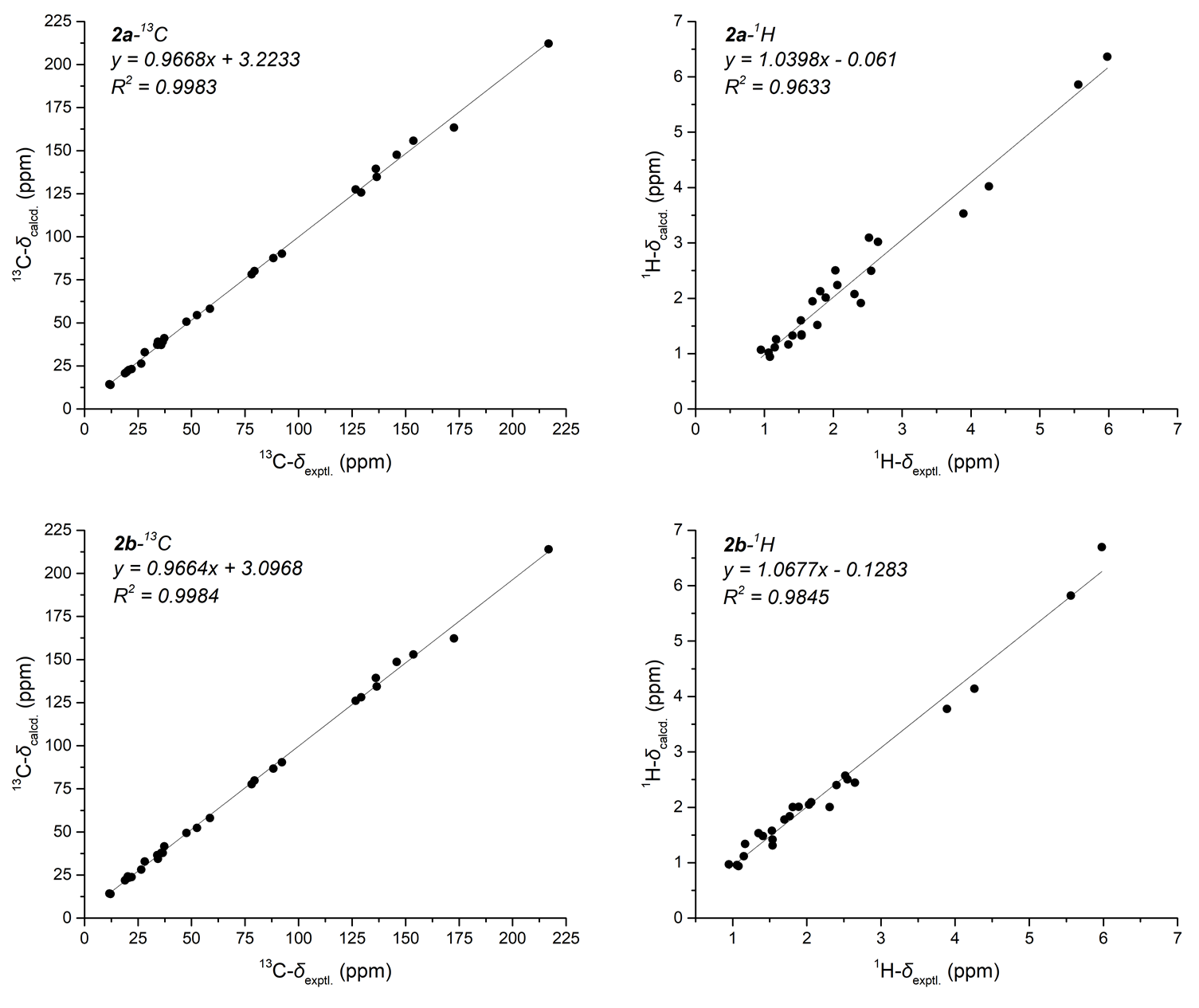


**Figure S72.** Linear regression analysis between the experimental and calculated NMR chemical shifts of **2a** and **2b**.

**Table S4.** The results of the DP4+ analysis


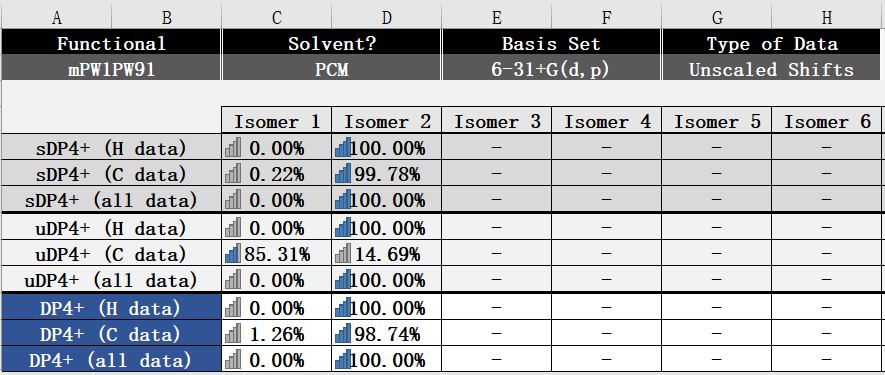


**Table S5.** The calculated spin-spin coupling constants of ^3^*J*_H-15/H-16_, ^3^*J*_H-15/C-8_, ^3^*J*_H-16/C-8_ in **2b** (*J* in Hz).

| Conformer | **2b**-1 | **2b**-2 | **2b**-3 | **2b**-4 | **2b**-5 | **2b**-6 | Averaged |
| --- | --- | --- | --- | --- | --- | --- | --- |
| Population | 49.58% | 18.40% | 15.78% | 9.93% | 5.05% | 1.26% |  |
| ^3^*J*_H-15/H-16_ | 0.4 | 0.6 | 0.4 | 0.5 | 0.6 | 0.6 | 0.5 |
| ^3^*J*_H-15/C-8_ | 5.5 | 5.2 | 5.5 | 5.4 | 5.1 | 5.2 | 5.4 |
| ^3^*J*_H-16/C-8_ | 7.0 | 7.2 | 7.0 | 6.6 | 7.2 | 6.6 | 7.0 |

Note: the calculation was run at B97-2/pcJ-1 level with IEFPCM solvent model in chloroform solvent using the M06-2X-D3/def2-SVP optimized geometries.

**4.2 Computational data of 2a**


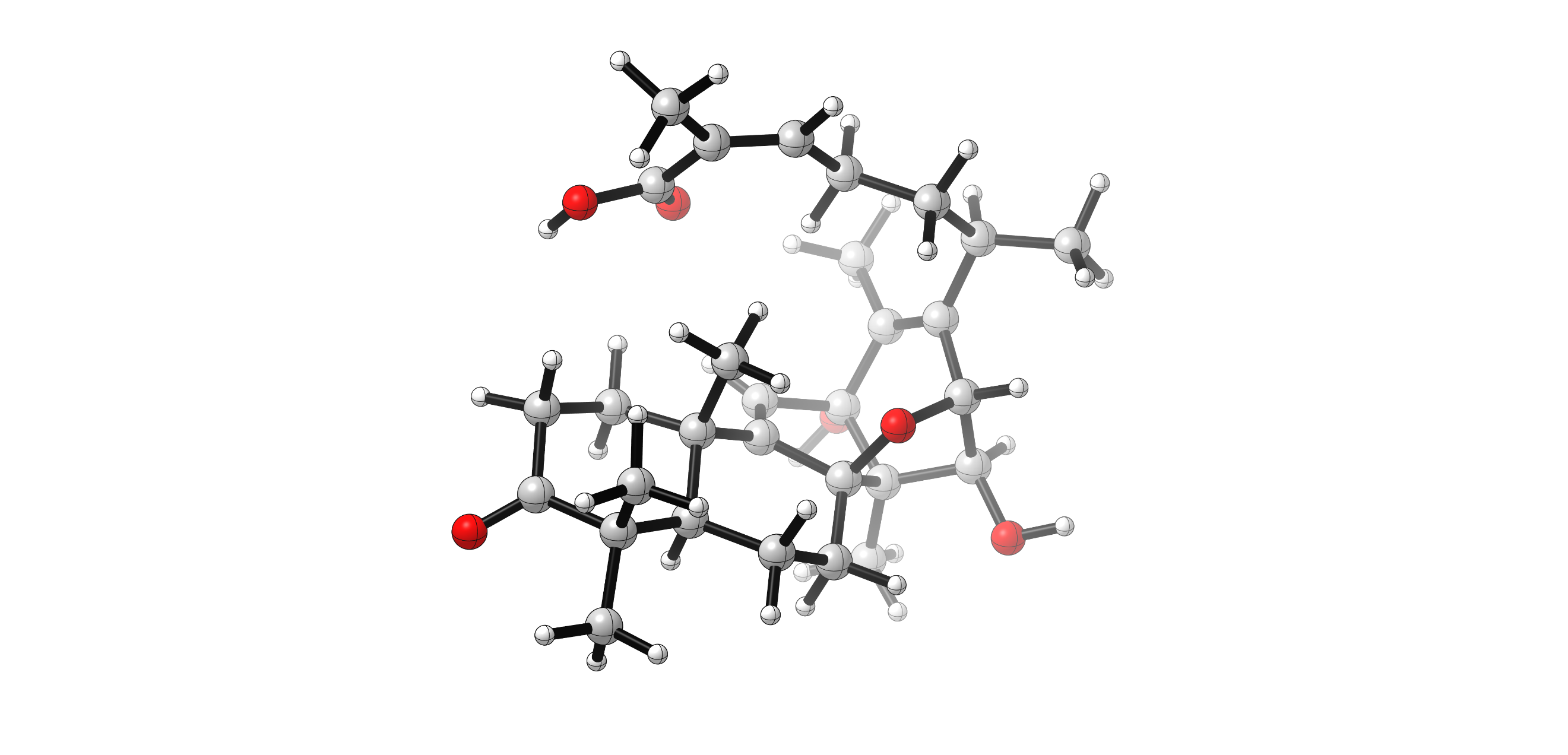

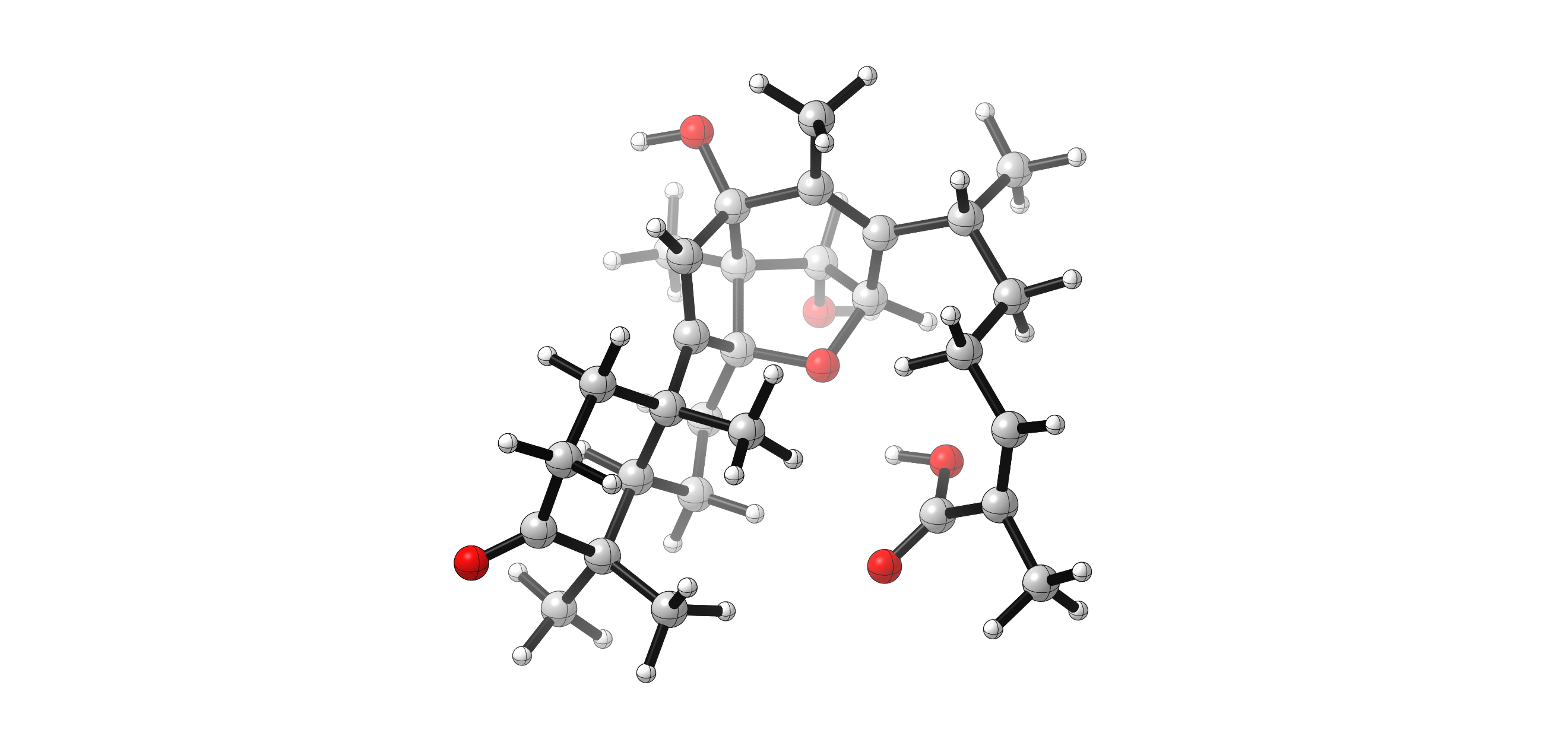


**Figure S73.** Optimized geometries of 2 dominant conformers of **2a** (**2a**-1 and **2a**-2, respectively) at the M06-2X-D3/def2-SVP level of theory in the gas phase

**Table S6.** Important thermodynamic parameters of the M06-2X-D3/def2-SVP optimized conformers of **2a** in the gas phase

| Conformers | E *^a^* (Hartree) | C *^b^* (Hartree) | G *^c^* (kcal/mol) |
| --- | --- | --- | --- |
| **2a**-1 | -1619.765968 | 0.615830 | -1016016.711620 |
| **2a**-2 | -1619.765858 | 0.616923 | -1016015.956662 |
| **2a**-3 | -1619.765977 | 0.617697 | -1016015.545650 |
| **2a**-4 | -1619.764829 | 0.618067 | -1016014.593073 |

*^a^* Electronic energy obtained at M06-2X-D3/def2-TZVP level of theory; *^b^* Thermal correction to Gibbs free energy obtained at M06-2X-D3/def2-SVP level of theory; *^C^* Gibbs free energy (E + C).

**Table S7.** Conformational analysis of the M06-2X-D3/def2-SVP optimized conformers of **2a** in the gas phase (T=298.15 K)

| Conformers | *Δ*G (kcal/mol) *^a^* | Population *^b^* |
| --- | --- | --- |
| **2a**-1 | 0.000000 | 69.11% |
| **2a**-2 | 0.754958 | 19.31% |
| **2a**-3 | 1.165970 | 9.65% |
| **2a**-4 | 2.118547 | 1.93% |

*^a^* The relative Gibbs free energy; *^b^* The Boltzmann distribution of each conformer.

**Table S8.** Optimized Z-matrixes of **2a**-1 in the gas phase (Å) at the M06-2X-D3/def2-SVP level of theory.

| C | 2.288480 | 0.101530 | 1.450500 | H | -0.249630 | 0.042140 | 2.541800 |
| --- | --- | --- | --- | --- | --- | --- | --- |
| C | 3.725910 | 0.494980 | 1.102740 | H | -3.970720 | -2.253360 | 0.053910 |
| C | 4.484040 | -0.657750 | 0.471180 | H | -3.088390 | -0.893950 | -2.119860 |
| C | 3.811510 | -1.369620 | -0.714990 | H | -2.216350 | 1.551510 | 2.517460 |
| C | 2.322340 | -1.650100 | -0.355590 | H | -3.808940 | 1.853740 | 1.798710 |
| C | 1.525560 | -2.342690 | -1.468430 | H | -3.603090 | 0.567990 | 3.020480 |
| C | 0.225790 | -2.924090 | -0.915150 | H | 0.999370 | 0.314580 | -1.773410 |
| C | -0.635710 | -1.843140 | -0.277660 | H | 2.072750 | 1.399500 | -0.849300 |
| C | 0.171490 | -0.963610 | 0.664890 | H | 0.357940 | 1.248050 | -0.427590 |
| C | 1.527260 | -0.449690 | 0.238460 | H | -4.232410 | 1.950550 | -0.083530 |
| C | -0.552490 | -0.631930 | 1.736820 | H | -5.447950 | 0.256010 | -1.567570 |
| C | -1.983840 | -1.135240 | 1.621740 | H | -5.330100 | 1.913820 | -2.201530 |
| C | -2.813310 | 0.046280 | 1.103110 | H | -4.276910 | 0.652210 | -2.853350 |
| C | -1.844450 | -2.306880 | 0.583910 | H | -1.954290 | 1.692580 | -2.095330 |
| C | -2.980230 | -2.291200 | -0.430350 | H | -3.085520 | 3.058760 | -2.085110 |
| C | -2.633510 | -0.974270 | -1.122900 | H | -2.442160 | 3.595460 | 0.272820 |
| C | -3.072690 | 0.163640 | -0.213580 | H | -1.369810 | 2.213350 | 0.329130 |
| C | -3.139900 | 1.065890 | 2.159430 | H | -0.932830 | 4.320410 | -1.866830 |
| C | 1.237800 | 0.689720 | -0.770400 | H | 1.890730 | 5.758500 | -0.507700 |
| C | -3.707150 | 1.378790 | -0.862170 | H | 1.089640 | 5.364090 | -2.054050 |
| C | -4.746430 | 1.022360 | -1.929950 | H | 2.420570 | 4.349970 | -1.429040 |
| C | -2.614970 | 2.293040 | -1.443770 | H | -1.583970 | -4.449590 | 0.542790 |
| C | -1.782650 | 2.966720 | -0.354090 | H | -2.547290 | -3.851250 | 1.914440 |
| C | -0.695610 | 3.832620 | -0.912320 | H | -0.770490 | -3.648030 | 1.914890 |
| C | 0.532730 | 4.078450 | -0.422850 | H | 5.066770 | -0.424410 | -2.210730 |
| C | 0.976650 | 3.461890 | 0.858940 | H | 3.631270 | 0.544660 | -1.821050 |
| C | 1.534660 | 4.937360 | -1.146540 | H | 3.469150 | -0.915560 | -2.818220 |
| C | -1.673100 | -3.644950 | 1.282170 | H | 4.226050 | -3.155720 | -1.898100 |
| C | 3.997540 | -0.478980 | -1.958040 | H | 4.385440 | -3.395950 | -0.135100 |
| C | 4.552290 | -2.687510 | -0.959920 | H | 5.631390 | -2.500660 | -1.020080 |
| H | 1.739430 | 0.960070 | 1.867800 | H | -1.909060 | -2.097360 | 3.301450 |
| H | 2.304580 | -0.673380 | 2.234920 | H | -3.508280 | -3.354580 | -1.966940 |
| H | 3.728220 | 1.339980 | 0.395340 | H | 2.532160 | 3.110780 | 1.853450 |
| H | 4.294300 | 0.804450 | 1.990420 | O | -2.549000 | -1.538230 | 2.846960 |
| H | 2.376070 | -2.379460 | 0.476540 | O | 2.314840 | 3.522480 | 1.002720 |
| H | 1.291040 | -1.639130 | -2.281630 | O | -1.222780 | -1.042900 | -1.321240 |
| H | 2.120680 | -3.152900 | -1.911720 | O | 0.279370 | 2.948620 | 1.698740 |
| H | -0.365380 | -3.428240 | -1.690920 | O | -2.848270 | -3.412370 | -1.268300 |
| H | 0.479520 | -3.669440 | -0.144880 | O | 5.570640 | -0.986340 | 0.878150 |

**Table S9.** Optimized Z-matrixes of **2a**-2 in the gas phase (Å) at the M06-2X-D3/def2-SVP level of theory.

| C | -2.610680 | -1.244340 | -1.955570 | H | -0.139080 | -2.504080 | -2.257410 |
| --- | --- | --- | --- | --- | --- | --- | --- |
| C | -3.941320 | -0.541040 | -2.234960 | H | 3.259930 | -2.250830 | 1.442210 |
| C | -4.808020 | -0.420010 | -0.993140 | H | 2.794790 | 0.428340 | 1.466470 |
| C | -4.140160 | 0.159110 | 0.264730 | H | 2.647010 | -2.967560 | -2.852340 |
| C | -2.774280 | -0.565410 | 0.455830 | H | 2.540690 | -1.239850 | -3.289620 |
| C | -2.017350 | -0.131460 | 1.716830 | H | 4.045830 | -1.904250 | -2.626220 |
| C | -0.920840 | -1.142640 | 2.046850 | H | -2.078850 | 1.393100 | -1.712280 |
| C | 0.078960 | -1.235570 | 0.905690 | H | -0.917200 | 1.354510 | -0.378560 |
| C | -0.616720 | -1.413190 | -0.441750 | H | -0.489910 | 0.638420 | -1.946590 |
| C | -1.843560 | -0.599290 | -0.792800 | H | 3.965140 | 0.311820 | -2.066780 |
| C | 0.091950 | -2.231360 | -1.224580 | H | 5.174100 | 0.225940 | 0.756220 |
| C | 1.419680 | -2.606250 | -0.589300 | H | 5.551190 | -0.892540 | -0.575850 |
| C | 2.466930 | -1.644150 | -1.163620 | H | 6.002090 | 0.831760 | -0.693970 |
| C | 1.131150 | -2.376690 | 0.939380 | H | 3.490690 | 2.133980 | 0.347360 |
| C | 2.314660 | -1.715360 | 1.634590 | H | 4.270680 | 2.550650 | -1.177420 |
| C | 2.272190 | -0.368800 | 0.916760 | H | 2.111750 | 2.077060 | -2.381720 |
| C | 2.837760 | -0.548990 | -0.477200 | H | 1.366070 | 1.619150 | -0.849420 |
| C | 2.950040 | -1.956370 | -2.559600 | H | 2.117410 | 4.441440 | -1.832220 |
| C | -1.309390 | 0.783070 | -1.227260 | H | 0.836790 | 6.108900 | 0.925990 |
| C | 3.869930 | 0.444610 | -0.978070 | H | 0.905080 | 6.243630 | -0.853060 |
| C | 5.229950 | 0.132310 | -0.340520 | H | -0.587620 | 5.685380 | -0.036480 |
| C | 3.490250 | 1.911760 | -0.731270 | H | 0.470840 | -3.470650 | 2.677110 |
| C | 2.118080 | 2.283450 | -1.297940 | H | 1.452210 | -4.423420 | 1.537590 |
| C | 1.743300 | 3.727710 | -1.088330 | H | -0.251250 | -4.041240 | 1.148000 |
| C | 0.965480 | 4.221320 | -0.112900 | H | -3.267680 | 2.114330 | 0.766990 |
| C | 0.369180 | 3.286800 | 0.894280 | H | -4.996390 | 2.152870 | 0.328260 |
| C | 0.511570 | 5.648740 | -0.019380 | H | -3.763940 | 1.996600 | -0.926810 |
| C | 0.669810 | -3.656320 | 1.614900 | H | -4.735840 | 0.456430 | 2.343110 |
| C | -4.027030 | 1.690770 | 0.092710 | H | -5.054130 | -1.190260 | 1.724190 |
| C | -5.051180 | -0.120930 | 1.463800 | H | -6.081280 | 0.161490 | 1.214070 |
| H | -1.992080 | -1.234440 | -2.866800 | H | 1.103390 | -4.509830 | -0.754990 |
| H | -2.801580 | -2.302060 | -1.709550 | H | 2.765210 | -1.162080 | 3.440510 |
| H | -3.757740 | 0.474410 | -2.621080 | H | 0.816110 | 1.679350 | 1.778930 |
| H | -4.530100 | -1.074920 | -2.991700 | O | 1.850880 | -3.914390 | -0.880110 |
| H | -3.047960 | -1.626820 | 0.619060 | O | 1.269180 | 2.489610 | 1.468270 |
| H | -1.587620 | 0.875910 | 1.590630 | O | 0.876700 | -0.028600 | 0.923040 |
| H | -2.709910 | -0.069690 | 2.566560 | O | -0.809490 | 3.239550 | 1.147810 |
| H | -0.378130 | -0.890430 | 2.968250 | O | 2.034200 | -1.615990 | 3.008070 |
| H | -1.386940 | -2.130130 | 2.191710 | O | -5.965690 | -0.757590 | -1.009730 |

**4.3 Computational data of 2b**


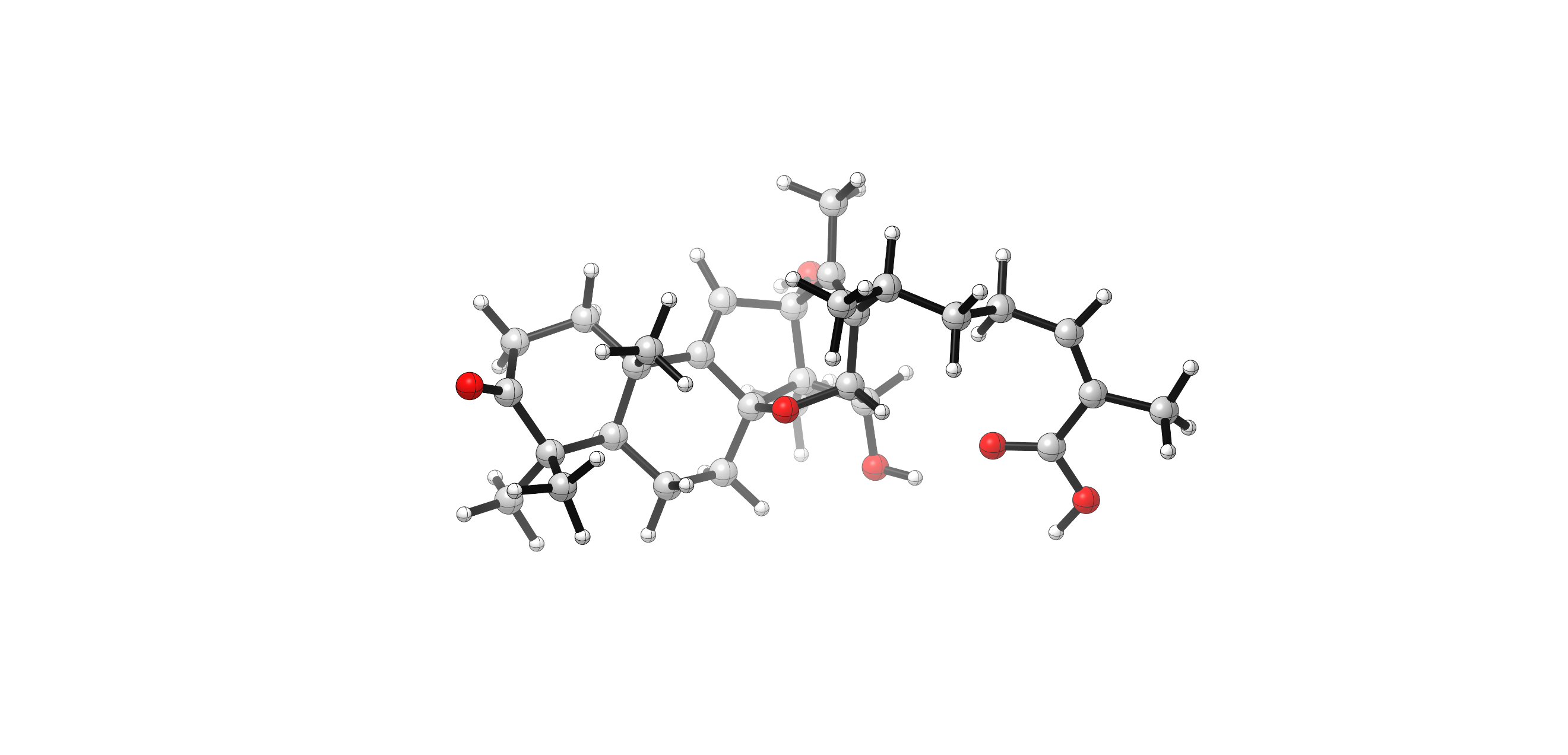

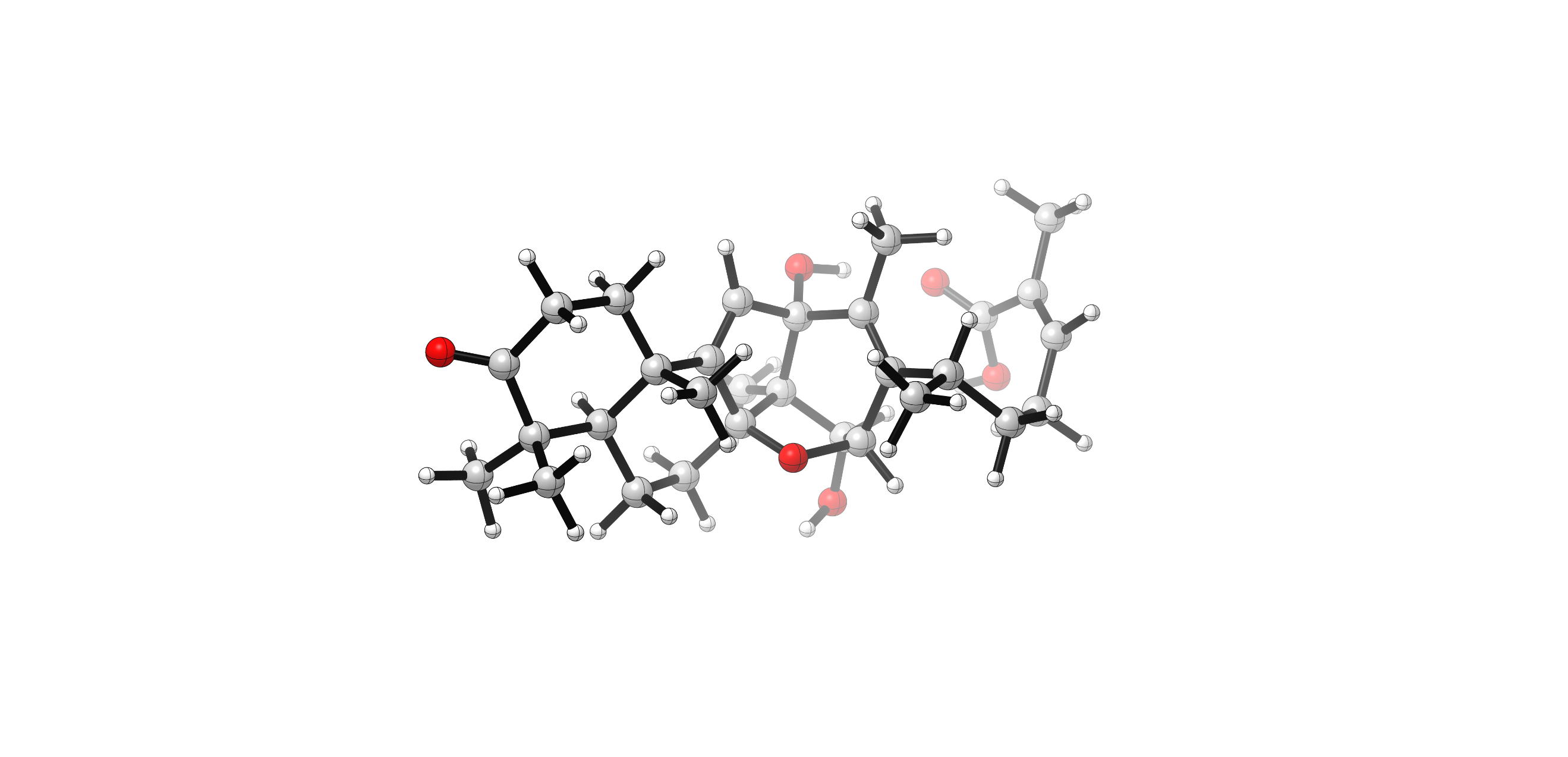


**Figure S74.** Optimized geometries of 2 dominant conformers of **2b** at the M06-2X-D3/def2-SVP level of theory in the gas phase

**Table S10.** Important thermodynamic parameters of the M06-2X-D3/def2-SVP optimized conformers of **2b**.

| Conformers | E *^a^* (Hartree) | C *^b^* (Hartree) | G *^c^* (kcal/mol) |
| --- | --- | --- | --- |
| **2b**-1 | -1619.769262 | 0.616901 | -1016018.106722 |
| **2b**-2 | -1619.769486 | 0.618060 | -1016017.519690 |
| **2b**-3 | -1619.769167 | 0.617886 | -1016017.428978 |
| **2b**-4 | -1619.770538 | 0.619694 | -1016017.154723 |
| **2b**-5 | -1619.770030 | 0.619824 | -1016016.754334 |
| **2b**-6 | -1619.766329 | 0.617430 | -1016015.934242 |

*^a^* Electronic energy obtained at M06-2X-D3/def2-TZVP level of theory; *^b^* Thermal correction to Gibbs free energy obtained at M06-2X-D3/def2-SVP level of theory; *^C^* Gibbs free energy (E + C).

**Table S11.** Conformational analysis of the M06-2X-D3/def2-SVP optimized conformers of **2b** (T=298.15 K)

| Conformers | *Δ*G (kcal/mol) *^a^* | Population *^b^* |
| --- | --- | --- |
| **2b**-1 | 0.000000 | 49.58% |
| **2b**-2 | 0.587033 | 18.40% |
| **2b**-3 | 0.677744 | 15.78% |
| **2b**-4 | 0.951999 | 9.93% |
| **2b**-5 | 1.352388 | 5.05% |
| **2b**-6 | 2.172480 | 1.26% |

*^a^* The relative Gibbs free energy; *^b^* The Boltzmann distribution of each conformer.

**Table S12.** Optimized Z-matrixes of **2b**-1 in the gas phase (Å) at the M06-2X-D3/def2-SVP level of theory.

| C | -2.660500 | -2.411110 | 2.453910 | H | -2.738090 | 0.361490 | 2.109910 |
| --- | --- | --- | --- | --- | --- | --- | --- |
| C | -2.929650 | -3.888800 | 2.804020 | H | -0.347960 | 2.446790 | -1.761110 |
| C | -1.745670 | -4.789790 | 2.522050 | H | 1.804150 | 0.976760 | -1.028690 |
| C | -1.249390 | -4.780560 | 1.069770 | H | -0.205360 | 3.458220 | 2.608810 |
| C | -1.529380 | -3.391440 | 0.421270 | H | -1.700020 | 3.828990 | 1.703150 |
| C | -0.643330 | -3.121800 | -0.798850 | H | -1.668670 | 2.471460 | 2.824960 |
| C | -1.071260 | -1.851380 | -1.523790 | H | -0.014950 | -3.048040 | 2.858480 |
| C | -0.921820 | -0.661170 | -0.590710 | H | -0.172430 | -1.276580 | 2.872900 |
| C | -1.634490 | -0.901590 | 0.733050 | H | 0.688610 | -2.057810 | 1.537510 |
| C | -1.502100 | -2.224190 | 1.451940 | H | 1.602060 | 3.021260 | 2.042200 |
| C | -2.202920 | 0.226820 | 1.166280 | H | 3.515640 | 1.413560 | 2.363060 |
| C | -1.848690 | 1.411190 | 0.279360 | H | 1.930550 | 0.645620 | 2.661170 |
| C | -0.663330 | 2.118760 | 0.953010 | H | 2.790610 | 0.336530 | 1.140740 |
| C | -1.472030 | 0.706630 | -1.074770 | H | 3.660260 | 3.434010 | 0.866810 |
| C | -0.242450 | 1.350040 | -1.699670 | H | 3.256880 | 2.310400 | -0.428230 |
| C | 0.778300 | 0.938130 | -0.641210 | H | 1.341260 | 3.724980 | -1.124940 |
| C | 0.595100 | 1.837580 | 0.567650 | H | 1.687930 | 4.866680 | 0.189380 |
| C | -1.062890 | 3.020860 | 2.087220 | H | 3.627800 | 5.740070 | -0.898380 |
| C | -0.164080 | -2.163050 | 2.222090 | H | 4.928420 | 6.245280 | -2.690530 |
| C | 1.862140 | 2.305760 | 1.248970 | H | 4.137690 | 5.896340 | -4.253730 |
| C | 2.568030 | 1.107510 | 1.894560 | H | 5.416810 | 4.832470 | -3.665990 |
| C | 2.817150 | 3.027550 | 0.283920 | H | -2.368260 | 0.135880 | -2.949520 |
| C | 2.145130 | 4.152970 | -0.514120 | H | -3.011430 | 1.643680 | -2.258760 |
| C | 3.134550 | 4.882140 | -1.371000 | H | -3.494270 | 0.066620 | -1.564380 |
| C | 3.534830 | 4.610120 | -2.627640 | H | 0.895520 | -4.453220 | 1.426730 |
| C | 2.958580 | 3.466740 | -3.387080 | H | 0.525790 | -5.444460 | -0.002650 |
| C | 4.561890 | 5.445300 | -3.345460 | H | 0.336750 | -6.124070 | 1.636290 |
| C | -2.661420 | 0.630800 | -2.015720 | H | -1.819950 | -5.876780 | -0.723390 |
| C | 0.218200 | -5.218660 | 1.027470 | H | -3.163930 | -5.656430 | 0.419970 |
| C | -2.084440 | -5.855630 | 0.344560 | H | -1.883530 | -6.848870 | 0.770860 |
| H | -2.433430 | -1.845440 | 3.369850 | H | -3.692530 | 1.892940 | -0.070850 |
| H | -3.575240 | -1.971380 | 2.030560 | H | 0.832400 | 1.196630 | -3.263420 |
| H | -3.222760 | -4.026280 | 3.851570 | H | 2.933200 | 2.708120 | -5.101780 |
| H | -3.753780 | -4.263140 | 2.175310 | O | -2.880950 | 2.361560 | 0.153490 |
| H | -2.571640 | -3.417580 | 0.048490 | O | 3.330950 | 3.480440 | -4.671910 |
| H | 0.408700 | -3.015830 | -0.492450 | O | 0.481090 | -0.431870 | -0.375210 |
| H | -0.690340 | -3.981770 | -1.485000 | O | 2.241640 | 2.592820 | -2.950750 |
| H | -0.471350 | -1.662900 | -2.424310 | O | 0.014080 | 0.784250 | -2.954320 |
| H | -2.127250 | -1.942840 | -1.825640 | O | -1.264760 | -5.508260 | 3.362730 |

**Table S13.** Optimized Z-matrixes of **2b**-2 in the gas phase (Å) at the M06-2X-D3/def2-SVP level of theory.

| C | -0.316680 | -2.452220 | 2.845430 | H | -2.105440 | -0.781450 | 1.563140 |
| --- | --- | --- | --- | --- | --- | --- | --- |
| C | 0.442450 | -3.285870 | 3.879570 | H | -0.864730 | 1.244910 | -2.893880 |
| C | 1.110230 | -4.493570 | 3.250700 | H | 1.545290 | 1.749050 | -1.811310 |
| C | 1.978160 | -4.245510 | 2.004060 | H | -2.289810 | 3.291820 | 0.696050 |
| C | 1.230100 | -3.272610 | 1.045010 | H | -3.344360 | 1.857560 | 0.606170 |
| C | 2.021930 | -2.900970 | -0.213880 | H | -2.091450 | 1.959680 | 1.856720 |
| C | 1.081060 | -2.330250 | -1.273150 | H | 2.275670 | -0.627020 | 1.390280 |
| C | 0.369390 | -1.078230 | -0.771580 | H | 2.164980 | -1.270880 | 3.052050 |
| C | -0.229090 | -1.324110 | 0.608620 | H | 1.014430 | -0.049060 | 2.479270 |
| C | 0.583360 | -2.008420 | 1.686740 | H | -0.335760 | 3.803230 | 0.630290 |
| C | -1.440690 | -0.772660 | 0.697170 | H | 2.053780 | 3.819930 | 1.463310 |
| C | -1.767790 | 0.073950 | -0.518890 | H | 1.150460 | 2.357790 | 1.944320 |
| C | -1.357490 | 1.505230 | -0.123080 | H | 2.323130 | 2.273300 | 0.614970 |
| C | -0.827220 | -0.546490 | -1.617310 | H | 1.239460 | 5.172930 | -0.506510 |
| C | -0.148360 | 0.530630 | -2.468280 | H | 1.943690 | 3.832290 | -1.397810 |
| C | 0.721360 | 1.156120 | -1.388450 | H | 0.451190 | 5.223870 | -2.825490 |
| C | -0.147900 | 1.987120 | -0.465630 | H | -0.087040 | 3.548240 | -2.756200 |
| C | -2.320890 | 2.200130 | 0.804900 | H | -1.267230 | 5.799090 | -0.980490 |
| C | 1.584770 | -0.941800 | 2.182040 | H | -3.546540 | 5.744740 | -0.517170 |
| C | 0.452680 | 3.262470 | 0.087020 | H | -4.247340 | 4.101010 | -0.630140 |
| C | 1.559640 | 2.911070 | 1.087620 | H | -4.516680 | 5.264720 | -1.936120 |
| C | 0.992020 | 4.214830 | -0.993920 | H | -0.891190 | -2.022020 | -3.188250 |
| C | 0.022210 | 4.459790 | -2.155970 | H | -2.412230 | -1.121630 | -2.957340 |
| C | -1.311020 | 4.939660 | -1.663040 | H | -1.970700 | -2.381200 | -1.794990 |
| C | -2.529120 | 4.417570 | -1.872620 | H | 3.282450 | -2.866760 | 3.158380 |
| C | -2.744880 | 3.208600 | -2.720130 | H | 3.972040 | -3.445890 | 1.627820 |
| C | -3.777690 | 4.916590 | -1.198690 | H | 3.866580 | -4.535240 | 3.029980 |
| C | -1.564250 | -1.587610 | -2.438350 | H | 2.947860 | -5.507090 | 0.507210 |
| C | 3.348230 | -3.731090 | 2.487660 | H | 1.264830 | -5.978100 | 0.879670 |
| C | 2.201650 | -5.592680 | 1.308440 | H | 2.558630 | -6.330070 | 2.037610 |
| H | -0.762970 | -1.571560 | 3.332900 | H | -3.368640 | 0.697810 | -1.429370 |
| H | -1.150590 | -3.047260 | 2.437740 | H | 1.408890 | -0.373940 | -3.156190 |
| H | 1.224140 | -2.674560 | 4.359260 | H | -2.278400 | 2.375860 | -4.332560 |
| H | -0.219230 | -3.655340 | 4.673240 | O | -3.131070 | -0.018520 | -0.820350 |
| H | 0.361400 | -3.862330 | 0.693400 | O | -2.069160 | 3.204770 | -3.871130 |
| H | 2.806920 | -2.164410 | 0.017790 | O | 1.321020 | 0.011110 | -0.766070 |
| H | 2.525870 | -3.787160 | -0.623330 | O | -3.496000 | 2.308320 | -2.423510 |
| H | 1.621410 | -2.115600 | -2.207340 | O | 0.607270 | 0.011010 | -3.531600 |
| H | 0.320820 | -3.090310 | -1.509330 | O | 0.978160 | -5.595300 | 3.722930 |

**Table S14.** Key transitions, oscillator strengths, and rotatory strengths in the ECD spectrum of conformer **2b**-1 at the CAM-B3LYP-SCRF/def2-SVP//M06-2X-D3/def2-SVP level of theory in MeOH with IEFPCM solvent model.

| *Num^a^* | *exited state^b^* | *CI Coefficient* | *ΔE (eV)^c^* | *λ (nm)^d^* | *f^e^* | *R_len_^f^* | *R_vel_^g^* |
| --- | --- | --- | --- | --- | --- | --- | --- |
| 1 | 133 ->138 | 0.63127 | 4.4624 | 277.84 | 0.0004 | 14.3309 | 9.6576 |
| 2 | 127 ->136 | 0.31154 | 5.3176 | 233.16 | 0.0188 | 9.8548 | 27.0838 |
|  | 128 ->136 | 0.54594 |  |  |  |  |  |
| 3 | 135 ->136 | 0.29011 | 5.5422 | 223.71 | 0.2041 | 178.7823 | 178.0985 |
|  | 135 ->137 | 0.56999 |  |  |  |  |  |
| 4 | 131 ->136 | 0.44276 | 5.7267 | 216.5 | 0.1808 | -180.8899 | -197.677 |
|  | 135 ->136 | -0.32605 |  |  |  |  |  |
|  | 135 ->137 | 0.33519 |  |  |  |  |  |
| 5 | 134 ->137 | 0.63689 | 5.7663 | 215.01 | 0.0238 | 51.7552 | 51.2013 |
| 6 | 131 ->136 | 0.44788 | 6.028 | 205.68 | 0.0587 | 43.6595 | 43.9816 |
|  | 135 ->136 | 0.5095 |  |  |  |  |  |
| 7 | 132 ->137 | 0.54461 | 6.2721 | 197.68 | 0.0621 | 1.9446 | -0.946 |
|  | 133 ->137 | 0.23306 |  |  |  |  |  |
| 8 | 132 ->136 | -0.24612 | 6.3998 | 193.73 | 0.001 | 0.9152 | 2.0487 |
|  | 134 ->136 | 0.61103 |  |  |  |  |  |
| 9 | 129 ->137 | 0.34393 | 6.769 | 183.17 | 0.0198 | -45.2821 | -44.7983 |
|  | 130 ->137 | 0.2903 |  |  |  |  |  |
|  | 134 ->139 | 0.33664 |  |  |  |  |  |
| 10 | 130 ->136 | -0.37802 | 6.8732 | 180.39 | 0.005 | -6.7366 | -6.3597 |
|  | 132 ->136 | 0.4176 |  |  |  |  |  |
|  | 134 ->136 | 0.26397 |  |  |  |  |  |
| 11 | 129 ->137 | 0.44981 | 6.9554 | 178.26 | 0.0131 | -8.218 | -9.147 |
|  | 134 ->139 | -0.2312 |  |  |  |  |  |
|  | 135 ->139 | 0.24771 |  |  |  |  |  |
| 12 | 134 ->138 | 0.31609 | 6.9788 | 177.66 | 0.0255 | -2.3689 | -1.1864 |
|  | 135 ->138 | 0.51851 |  |  |  |  |  |
| 13 | 130 ->136 | 0.34578 | 7.1161 | 174.23 | 0.0137 | -16.9819 | -17.0564 |
|  | 132 ->136 | 0.27686 |  |  |  |  |  |
|  | 133 ->136 | 0.33508 |  |  |  |  |  |
| 14 | 130 ->137 | 0.25743 | 7.1481 | 173.45 | 0.1547 | -24.5708 | -26.0572 |
|  | 135 ->139 | 0.51249 |  |  |  |  |  |
| 15 | 133 ->137 | 0.41368 | 7.2435 | 171.17 | 0.0348 | 34.6634 | 35.1703 |
|  | 134 ->139 | -0.26056 |  |  |  |  |  |
|  | 135 ->139 | -0.25195 |  |  |  |  |  |
| 16 | 130 ->137 | -0.32742 | 7.3011 | 169.82 | 0.0695 | 19.5112 | 18.221 |
|  | 134 ->139 | 0.34542 |  |  |  |  |  |
|  | 135 ->139 | 0.23886 |  |  |  |  |  |
| 17 | 119 ->136 | 0.25129 | 7.3216 | 169.34 | 0.002 | -10.7588 | -9.5233 |
|  | 121 ->136 | 0.31595 |  |  |  |  |  |
|  | 130 ->136 | -0.23952 |  |  |  |  |  |
|  | 133 ->136 | -0.26919 |  |  |  |  |  |
| 18 | 134 ->138 | 0.49619 | 7.3344 | 169.04 | 0.0046 | -9.615 | -10.1764 |
|  | 135 ->138 | -0.28077 |  |  |  |  |  |
| 19 | 122 ->136 | 0.28693 | 7.3636 | 168.37 | 0.0106 | -12.2226 | -12.8095 |
| 20 | 127 ->137 | 0.43132 | 7.4179 | 167.14 | 0.0591 | -42.3817 | -42.7471 |
|  | 128 ->137 | -0.28942 |  |  |  |  |  |
| 21 | 130 ->136 | -0.29759 | 7.5044 | 165.22 | 0.0055 | -7.132 | -6.8563 |
|  | 132 ->136 | -0.28227 |  |  |  |  |  |
|  | 133 ->136 | 0.4462 |  |  |  |  |  |
| 22 | 124 ->138 | -0.24775 | 7.5455 | 164.32 | 0.0258 | -32.171 | -30.0788 |
|  | 125 ->138 | 0.24661 |  |  |  |  |  |
|  | 132 ->138 | 0.32911 |  |  |  |  |  |
| 23 | 135 ->140 | 0.39327 | 7.637 | 162.35 | 0.0331 | 37.4785 | 41.4202 |
|  | 135 ->141 | 0.43664 |  |  |  |  |  |
| 24 | 126 ->137 | -0.29151 | 7.676 | 161.52 | 0.0393 | 55.54 | 56.1315 |
|  | 132 ->139 | 0.42028 |  |  |  |  |  |
| 25 | 123 ->138 | -0.22749 | 7.7014 | 160.99 | 0.0179 | -31.4458 | -29.0786 |
|  | 129 ->138 | 0.26391 |  |  |  |  |  |
| 26 | 120 ->136 | -0.23345 | 7.7438 | 160.11 | 0.061 | 17.0942 | 18.6906 |
|  | 129 ->136 | 0.40528 |  |  |  |  |  |
| 27 | 126 ->137 | 0.38365 | 7.7725 | 159.52 | 0.0216 | -14.5148 | -17.9779 |
| 28 | 120 ->136 | 0.23863 | 7.8264 | 158.42 | 0.0486 | 23.9988 | 26.0865 |
|  | 129 ->136 | 0.43734 |  |  |  |  |  |
| 29 | 124 ->138 | 0.37429 | 7.9172 | 156.6 | 0.0237 | -3.2267 | -9.7608 |
|  | 126 ->138 | 0.25579 |  |  |  |  |  |
|  | 133 ->140 | -0.25271 |  |  |  |  |  |
| 30 | 127 ->136 | -0.26512 | 7.961 | 155.74 | 0.0109 | 6.5072 | 7.9748 |
|  | 131 ->137 | 0.44182 |  |  |  |  |  |

*^a^*Number of the excited states. *^b^*only excited states with contribution over 10% were listed. *^c^*Excitation energy. *^d^*Wavelength. *^e^*Oscillator strength. *^f^*Rotatory strength in length form (10^-40^ cgs.). *^g^*Rotatory strength in velocity form (10-40 cgs.).
